# GC/MS-based ^13^C metabolic flux analysis resolves the parallel and cyclic photomixotrophic metabolism of *Synechocystis* sp. PCC 6803 and selected deletion mutants including the Entner-Doudoroff and phosphoketolase pathways

**DOI:** 10.1186/s12934-022-01790-9

**Published:** 2022-04-22

**Authors:** Dennis Schulze, Michael Kohlstedt, Judith Becker, Edern Cahoreau, Lindsay Peyriga, Alexander Makowka, Sarah Hildebrandt, Kirstin Gutekunst, Jean-Charles Portais, Christoph Wittmann

**Affiliations:** 1 Institute of Systems Biotechnology, Saarland University, Saarbrücken, Germany; 2 Toulouse Biotechnology Institute, Université de Toulouse, CNRS, INRAE, INSA, Toulouse, France; 3 MetaboHUB-MetaToul, National Infrastructure of Metabolomics & Fluxomics, Toulouse, France; 4 RESTORE, Université de Toulouse, Inserm U1031, CNRS 5070, UPS, EFS, Toulouse, France; 5 Institute of Botany, Christian-Albrecht University, Kiel, Germany; 6 Molecular Plant Physiology, Bioenergetics in Photoautotrophs, University of Kassel, Kassel, Germany

**Keywords:** ^13^C metabolic flux analysis, Cyanobacteria, GC–MS, NMR, Glycolytic shunt, Oxidative pentose phosphate pathway, Calvin-Benson-Bassham cycle, Entner-Doudoroff pathway, Phosphoketolase pathway, TCA cycle, photomixotrophic growth, Glucose, CO_2_

## Abstract

**Background:**

Cyanobacteria receive huge interest as green catalysts. While exploiting energy from sunlight, they co-utilize sugar and CO_2_. This photomixotrophic mode enables fast growth and high cell densities, opening perspectives for sustainable biomanufacturing. The model cyanobacterium *Synechocystis* sp. PCC 6803 possesses a complex architecture of glycolytic routes for glucose breakdown that are intertwined with the CO_2_-fixing Calvin-Benson-Bassham (CBB) cycle. To date, the contribution of these pathways to photomixotrophic metabolism has remained unclear.

**Results:**

Here, we developed a comprehensive approach for ^13^C metabolic flux analysis of *Synechocystis* sp. PCC 6803 during steady state photomixotrophic growth. Under these conditions, the Entner-Doudoroff (ED) and phosphoketolase (PK) pathways were found inactive but the microbe used the phosphoglucoisomerase (PGI) (63.1%) and the oxidative pentose phosphate pathway (OPP) shunts (9.3%) to fuel the CBB cycle. Mutants that lacked the ED pathway, the PK pathway, or phosphofructokinases were not affected in growth under metabolic steady-state. An ED pathway-deficient mutant (*Δeda*) exhibited an enhanced CBB cycle flux and increased glycogen formation, while the OPP shunt was almost inactive (1.3%). Under fluctuating light, *∆eda* showed a growth defect, different to wild type and the other deletion strains.

**Conclusions:**

The developed approach, based on parallel ^13^C tracer studies with GC–MS analysis of amino acids, sugars, and sugar derivatives, optionally adding NMR data from amino acids, is valuable to study fluxes in photomixotrophic microbes to detail. In photomixotrophic cells, PGI and OPP form glycolytic shunts that merge at switch points and result in synergistic fueling of the CBB cycle for maximized CO_2_ fixation. However, redirected fluxes in an ED shunt-deficient mutant and the impossibility to delete this shunt in a GAPDH2 knockout mutant, indicate that either minor fluxes (below the resolution limit of ^13^C flux analysis) might exist that could provide catalytic amounts of regulatory intermediates or alternatively, that EDA possesses additional so far unknown functions. These ideas require further experiments.

**Supplementary Information:**

The online version contains supplementary material available at 10.1186/s12934-022-01790-9.

## Background

^13^C metabolic flux analysis is a powerful approach to analyze metabolic networks in living cells and quantify the use of intracellular pathways, i.e., in vivo reaction rates (fluxes) [1, 2]. Notably, ^13^C metabolic flux studies have greatly contributed to understanding the lifestyle of microbes, including prominent biotechnological members such as *Escherichia coli* [3, 4], *Corynebacterium glutamicum* [5, 6], *Bacillus subtilis* [7, 8]*, Pseudomonas putida* [9, 10] as well as lactic and acetic acid bacteria [11, 12]. State-of-art ^13^C metabolic flux analysis grows the cells of interest on specific ^13^C tracer substrates, whose cellular metabolite ^13^C-labelling patterns are then analyzed as input for a software-based flux calculation [1, 2]. For the generation of informative ^13^C patterns, different types of mass spectrometry (MS) [13–16] and nuclear magnetic resonance spectroscopy (NMR) [17–19] are applied, alone or in combination [20, 21]. Parallel isotope cultures on different ^13^C tracers provide ^13^C data sets with enhanced information content which helps to tackle more complex scenarios [22–24].

A diverse group of photosynthetic bacteria that arouse substantial interest to be studied in their metabolic flux properties is the clade of cyanobacteria [25–27]. These microbes are regarded promising light-driven green catalysts to produce bioactive compounds [28–30], biodegradable polymers [31, 32], chemicals [33], biofuels [34], and hydrogen [35] Furthermore, cyanobacteria are suggested important for future terraforming of Mars [36]. The progress achieved and the huge potential ahead have continuously increased the interest in the different lifestyles of cyanobacteria [37]. This includes photoautotrophic fixation of CO_2_ under light [38] and also heterotrophic growth that takes place in the dark, although at much lower rates [39].

**Fig. 1:**
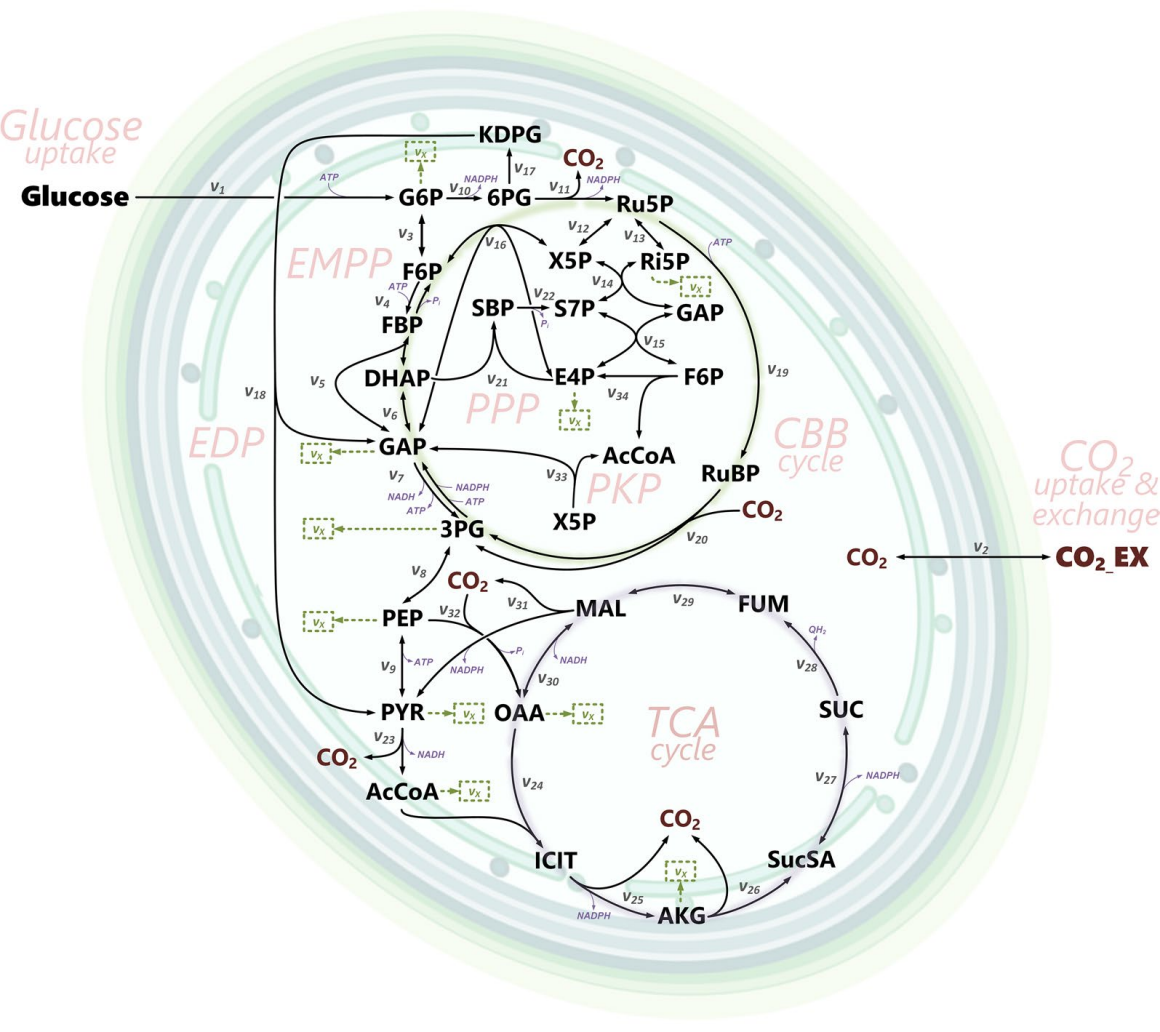
Carbon core metabolic network of *Synechocystis* 6803 for the co-utilization of glucose and CO_2_ under photomixotrophic conditions. The network comprises the Calvin-Bassham-Benson (CBB) cycle, the Emden-Meyerhof-Parnas (EMP) pathway (including the PGI shunt), the Entner-Doudoroff (ED) pathway (including the ED shunt), the oxidative pentose phosphate (OPP) pathway (including the OPP shunt), the phosphoketolase (PK) pathway, the TCA cycle, and anabolic routes. Abbreviations:, glucose 6-phosphate (G6P), fructose 6-phosphate (F6P), dihydroxyacetone phosphate (DHAP), glyceraldehyde 3-phosphate (GAP), 3-phosphoglycerate (3PG), phosphoenolpyruvate (PEP), pyruvate (PYR), acetyl-CoA (AcCoA), isocitrate (ICI), 2-oxoglutarate (2OG), succinate semialdehyde (SucA), succinate (SUC), fumarate (FUM), malate (MAL), oxaloacetate (OAA), 6-phosphogluconate (6PG), 2-keto-3-deoxy-6-phosphogluconate (KDPG), ribose 5-phosphate (Ri5P), ribulose 5-phosphate (Ru5P), xylose 5-phosphate (X5P), sedoheptulose 7-phosphate (S7P), erythrose 4-phosphate (E4P), extracellular carbon dioxide (CO2_EX), intracellular carbon dioxide (CO2). The reaction numbers refer to the biochemical network, used for flux estimation (Additional file 1: Table S9). Anabolic fluxes into biomass (X) are given as v_x_

While exploiting energy from sunlight, cyanobacteria are, notably, also able to efficiently co-utilize sugar and CO_2_ [34, 40, 41]. This photomixotrophic mode enables fast growth, opening the most promising perspectives for biomanufacturing [42]. *Synechocystis* sp. PCC 6803 (here *Synechocystis* 6803) is the major model bacterium of the cyanobacteria clade. For photomixotrophic glucose degradation, the microbe intertwines the Emden-Meyerhof-Parnas (EMP) pathway, the oxidative pentose phosphate (OPP) pathway, the Calvin-Benson-Bassham (CBB) cycle, the phosphoketolase (PK) pathway, the Entner-Doudoroff (ED) pathway, and an unusual TCA cycle into a highly redundant cyclic and parallelized architecture [43–45]. Without doubt, this network is much more complex than that of other bacteria (Fig. 1). To date, the contribution of the individual pathways to photomixotrophic growth is not known.

Interestingly, photomixotrophic growth yields are often higher than the sum of photoautotrophic and heterotrophic yields as has been shown for the cyanobacteria *Synechocystis* [43], *Spirulina* [46], and *Nostoc* [47] but also in the microalga *Chlorella vulgaris* [48]. This indicates that (uncompartmented) photomixotrophy is more than a simple addition of photoautotrophy (anabolism) and heterotrophy (catabolism) but rather a different metabolism. For further exploration of cyanobacteria, the investigation of their photomixotrophic metabolism appears relevant, as its quantitative understanding holds the key to optimized cell factories with streamlined pathways, as demonstrated for other microbes, used in industry [5, 22, 49].

Here, we present an approach for GC/MS-based ^13^C metabolic flux analysis to elucidate intracellular fluxes of photomixotrophic *Synechocystis* 6803 in full detail. Developed by computer-based experimental design, the approach determined fluxes through the EMP pathway, the OPP pathway, the ED pathway, the CBB cycle, the PK pathway, and the TCA cycle. Technically, it integrated parallel isotope experiments on [1-^13^C], [3-^13^C], [6-^13^C], and [^13^C_6_] glucose and 388 GC/MS-based mass isotopomers of proteinogenic amino acids, sugars and sugar derivatives from the cell wall [9], and sugars from glycogen and RNA [50]. The additional consideration of 168 positional ^13^C amino acid enrichments from 1 and 2D NMR analysis [51, 52] increased the precision of the estimated fluxes. Using the GC/MS-based approach, the wildtype and selected deletion mutants of *Synechocystis* 6803 were studied, providing in-depth insight into functional network operation of this green catalyst.

## Results

### Development of a suitable workflow for 13C isotope studies of *Synechocystis* 6803

**Fig. 2:**
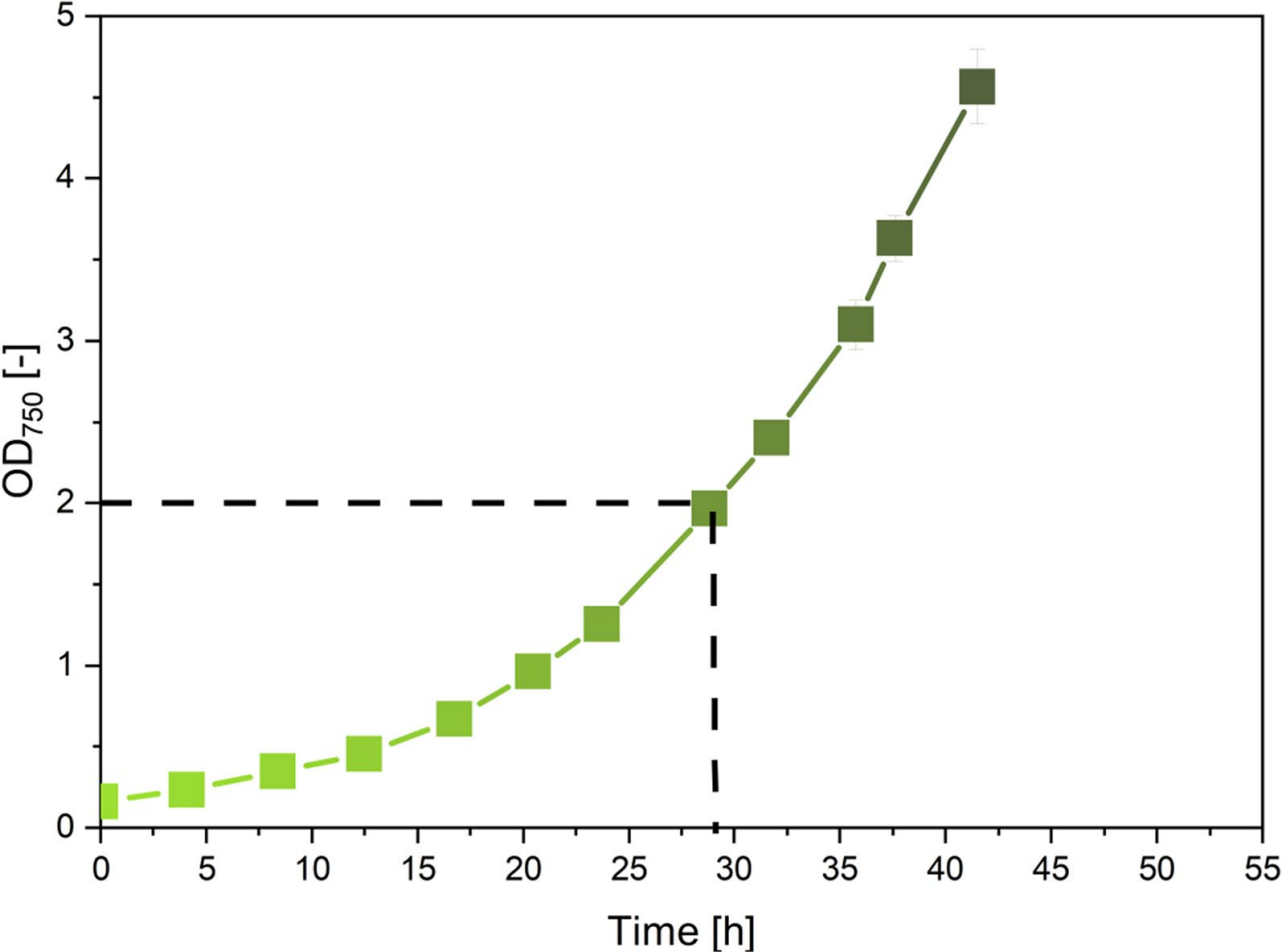
Photomixotrophic growth of *Synechocystis* 6803 on glucose with ambient CO_2_ levels and constant light (35 µE m^−2^ s^−1^). The dashed line indicates the cell concentration up to which the cells grew non-limited by light (Additional file 1: Fig. S1). n = 3

First, we developed a protocol to grow the cyanobacterium on ^13^C-labelled glucose under metabolic and isotopic steady-state and without interfering inoculum effects, important prerequisites for the chosen flux approach [53]. In short, cells were cultivated under constant (non-limiting) light on the corresponding ^13^C tracer in two steps: a ^13^C pre-culture (inoculated from non-labelled cells and grown from OD_750_ = 0.1 to OD_750_ =1.5) was inoculated into a subsequent ^13^C main culture (again grown from OD_750_ = 0.1 to OD_750_ =1.5) (Fig. 2). Doing so, the non-labelled cells, added initially, represented less than 0.5% of the cell amount harvested, important for the later ^13^C labelling analysis [54]. The common protocol of using just a one-step ^13^C tracer culture [9, 21, 22], inoculated (for negligible interference) with non-labelled cells at very low initial concentration (OD_750_ < 0.01) caused long lag phases and non-reproducible growth behavior and was therefore not further considered (data not shown). Using the developed two-step protocol, *Synechocystis* 6803 exhibited a constant specific growth rate, when supplied sufficiently with light (Additional file 1: Fig. S1) which proved metabolic steady state [53]. Moreover, the ^13^C labeling pattern of cellular metabolites did not change of time (Additional file 1: Fig. S2) which confirmed that the cultures were also in isotopic steady state [53]. Therefore, the setup was useful to investigate steady state photomixotrophic growth.

### Common strategies do not provide sufficient 13C labelling information to determine photomixotrophic fluxes of *Synechocystis* 6803 to full detail

The high complexity of the metabolic network (Fig. 1) posed a challenge on the flux estimation. We used computer-based experimental design to develop a suitable experimental strategy [55]. In short, we investigated different set-ups using Monte-Carlo analysis [9]. For each scenario, Monte-Carlo analysis yielded 100 (experimentally realistic) flux distributions. We then extracted statistical information from these data, e.g., standard deviations and confidence intervals, which indicated how precise individual flux values were determinable by the assumed strategy.

We first investigated three experimental approaches that had been previously applied for metabolic flux analysis of cyanobacteria, considering less detailed networks and using single isotope experiments and (mainly) GC/MS analysis of proteinogenic amino acids. Tested for different flux distributions, none of them allowed to determine all fluxes of the detailed network (Additional file 1: Fig. S3). As example, a mixture of [1-^13^C] and [^13^C_6_] glucose [56] resulted in generally larger errors and underestimated the EMP pathway flux. The use of [1, 2] ^13^C glucose [57] did not provide a reliable flux value for the EMP pathway and even failed to estimate the direction of flux through this route. A single tracer approach with [1-^13^C] glucose [58] was found unsuitable in assessing the PK, the PP, and the EMP pathway flux.

### Four parallel isotope experiments on different 13C tracers allow to determine all fluxes in the photomixotrophic carbon core metabolism

**Fig. 3:**
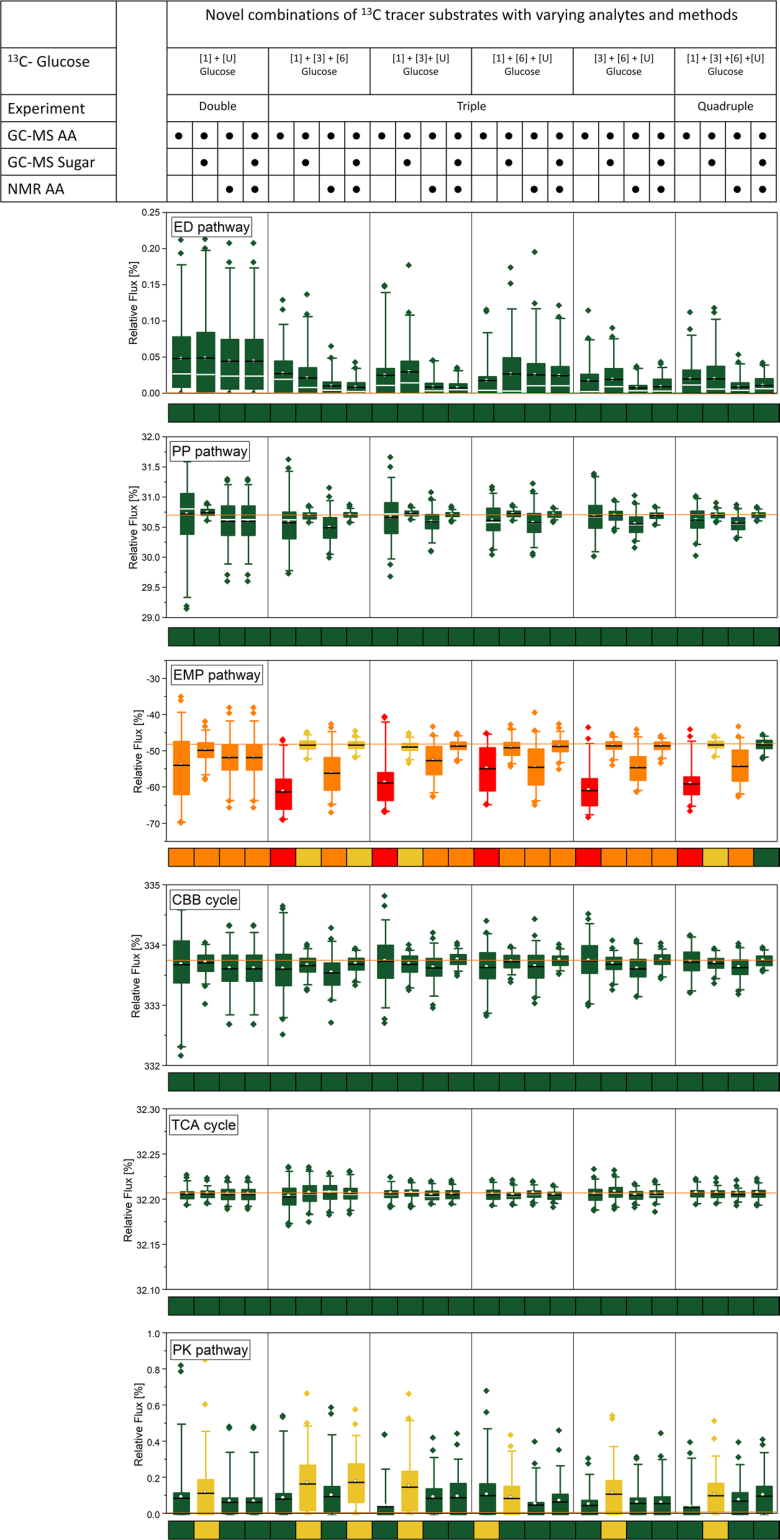
Experimental design for ^13^C metabolic flux analysis of photomixotrophic *Synechocystis* 6803. Different setups using different combinations of ^13^C tracer substrates and ^13^C labelling data were analyzed for achievable flux precision and accuracy, assuming a flux scenario with zero flux through the ED and the PK pathway. Here, the key fluxes of upper and lower carbon metabolism, i.e., through the ED, OPP, EMP, and PK pathways, the CBB cycle, and the TCA cycle, are shown. Each setup was evaluated by a Monte-Carlo approach that mimicked 100 repetitions of the corresponding flux study while taking experimental errors into account. The sensitive substrates [1-^13^C], [3-^13^C], [6-^13^C], and [^13^C_6_] glucose seemed useful for the following reasons. The combination of [1-^13^C] glucose and [6-^13^C] glucose well discriminated the fluxes through the EMP, the PP, and the ED pathway in glucose-grown pseudomonads, revealing a similarly cyclic pathway architecture as cyanobacteria [9]. Metabolization of [3-^13^C] glucose (based on the underlying carbon transitions) via the ED route should selectively lead to ^13^C label enrichment at the C_1_ of pyruvate (and amino acids derived therefrom), providing a sensitive readout, should this pathway be active. The use of [^13^C_6_] glucose appeared beneficial, likely because it helped to estimate the relative uptake of ^13^C sugar versus (non-labelled) CO_2_, as previously demonstrated for *Basfia succiniciproducens*, grown on sucrose under high rates of CO_2_ assimilation [22]. The color indicates flux determinability: green, < 0.1%, yellow < 1%, orange < 10%; and red, > 10%

The simulation-based approach was extended to evaluate a range of other ^13^C tracers, applied alone or in combination, and different analytical setups in terms of assessed metabolites and type of labelling information (mass isotopomers from GC/MS and positional ^13^C enrichment from NMR). The experimental design was conducted for different flux distributions which all yielded the same findings. One of the scenarios is exemplarily discussed below (Fig. 3). The concurring outcome for the other flux distributions can be taken from the supplement (Additional file 1: Figs. S4–S6). Different to single tracer studies, a two-tracer strategy with [1-^13^C] and [^13^C_6_] glucose, yielded improved determinability (Fig. 3). Although larger uncertainties regarding the EMP flux remained, parallel tracer experiments, obviously, delivered helpful information for the flux determination. The use of [^13^C_6_] glucose as second tracer obviously supported to estimate the relative uptake of the sugar versus (non-labelled) CO_2_, as demonstrated for ^13^C flux analysis of sucrose-grown *Basfia succiniciproducens* under high rates of CO_2_ assimilation [22].

Considering [1-^13^C], [3-^13^C], [6-^13^C], and [^13^C_6_] glucose as promising tracers, we then simulated a range of strategies that were based on triple and quadruple isotope studies (Fig. 3). In short, several triple combinations, each using three of the four tracers, plus the quadruple approach, using all four tracers, appeared useful. Only triple tracer strategies that exclusively relied on labelling data of protein-based amino acids somewhat underestimated the EMP pathway and created larger errors (independent if the ^13^C labelling patterns were derived from GC/MS, NMR, or from both tools together). It therefore appeared important to integrate the complementary labelling information from sugars and sugar derivatives. This was likely due to the fact that the analytes glucose (from storage carbohydrates), glucosamine (from cell wall constituents) and ribose (from RNA) represented the ^13^C labelling pattern of glucose 6-phosphate, fructose 6-phosphate, and ribose 5-phosphate, respectively, which are central hubs in the photomixotrophic metabolism of *Synechocystis* 6803 (Fig. 1). It should be noticed that the addition of NMR-based amino acid labelling patterns, i.e., positional ^13^C enrichments, to the data set resulted in the highest precision (Fig. 3), making this strategy attractive for scenarios that aim at the discrimination of small flux differences. From a practical point of view, the approach four parallel isotope studies on the different tracers plus GC/MS labelling analysis of amino acids, sugars, and sugar derivatives appeared most suitable. It coupled a reasonable workload to very good precision.

### Single mass isotopomer ratios sensitively reflect pathway fluxes but do not function as pathway specific indicators due to high network connectivity

We also investigated the possibility to use individual labelling data as qualitative and/or quantitative indicators of selected pathways. For this purpose, we evaluated changes in the abundance of mass isotopomers upon changes in a flux parameter of interest. Precisely said, the ratio between two different mass isotopomers of the same analyte was considered as readout [55]. Mass isotopomer ratios were sensitively influenced by a change in flux. Exemplified for the ED and the PK pathways, sensitive analytes were amino acids fragments, originating from pyruvate, such as leucine *m/z* 274, and, furthermore, sugar fragments like ribose *m/z* 307 (Additional file 1: Fig. S7). On a first glance, this suggested the possibility to use such a fragment as indicator for the activity of a certain pathway. However, none of the labelling patterns responded to only one single flux parameter alone because the highly intertwined network structure connected several pathways to the labelling state of a particular analyte. As example, pyruvate (derived amino acids such as leucine) can be derived from glucose through the EMP, the ED, the OPP pathways, and the CBB cycle (Fig. 1). Furthermore, also carbon from the TCA cycle can fuel the pyruvate pool. This made the independent estimation of a single flux parameter infeasible. Global flux estimation remained as sole approach to access the intracellular flux distribution.

### The ED and the PK pathways are inactive in photomixotrophic *Synechocystis* 6803

**Fig. 4:**
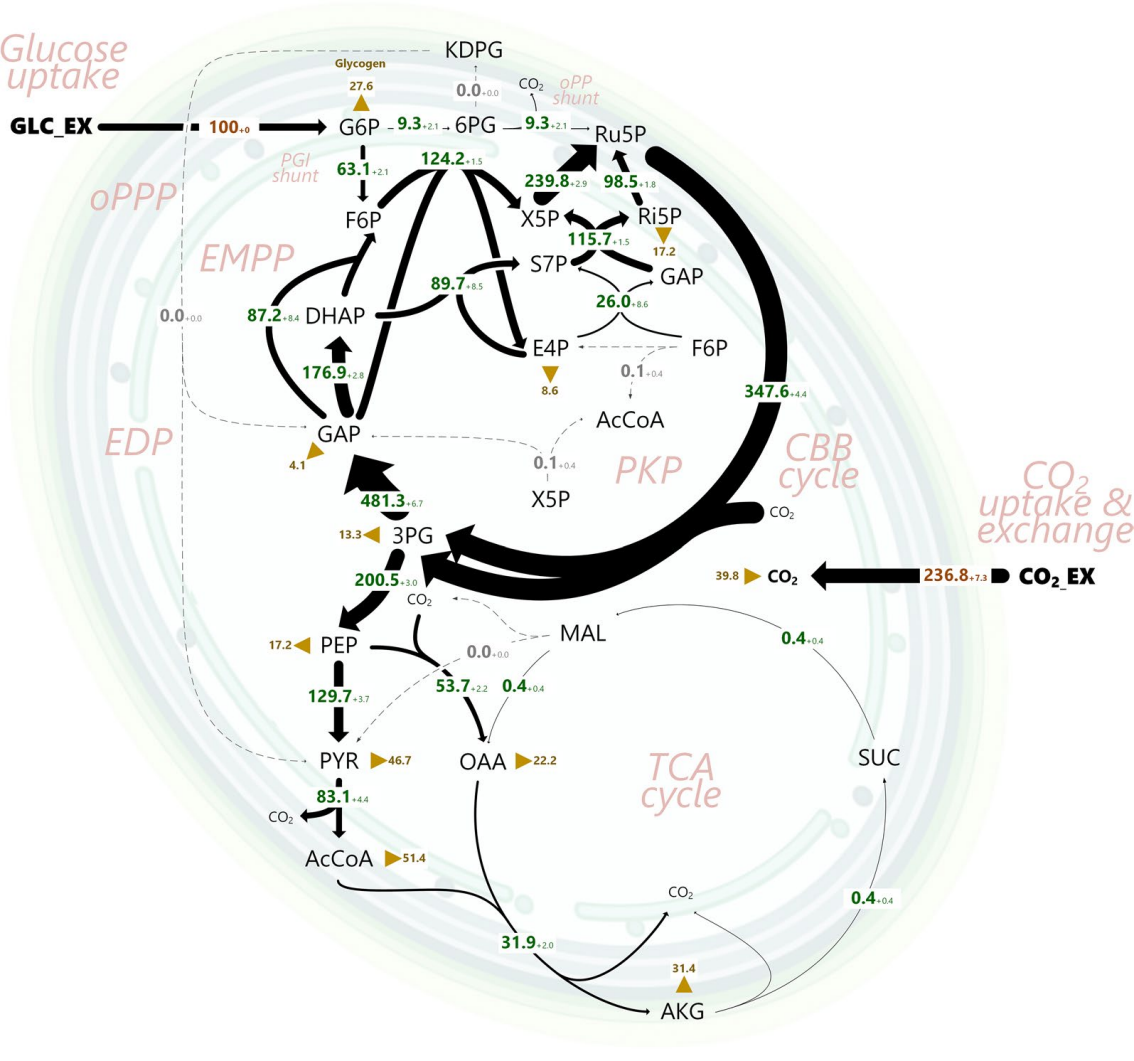
In vivo flux distribution of *Synechocystis* 6803 during photomixotrophic growth on glucose and CO_2_ determined by GC–MS based ^13^C metabolic flux analysis. Fluxes are normalized to the glucose uptake (100%, 0.421 mmol g^−1^ h^−1^). The thickness of the arrows denotes the amount of flux. The errors for the fluxes reflect standard deviations, estimated by Monte-Carlo simulation. The anabolic fluxes into biomass are shown as triangles. The complete flux data set is given in Additional file 1: Table S2, where also the 95% confidence intervals from the Monte-Carlo analysis are provided. *GLC_ex* extracellular glucose; *G6P* glucose 6-phosphate; *F6P* fructose 6-phosphate; *DHAP* dihydroxyacetone phosphate; *GAP* glyceraldehyde 3-phosphate; *3PG* 3-phosphoglycerate; *PEP* phosphoenolpyruvate; *PYR* pyruvate; *AcCoA* acetyl coenzyme A; *ICI* isocitrate; *2OG* 2-oxoglutarate; *SucA* succinate-semialdehyde; *SUC* succinate; *FUM* fumarate; *MAL* malate; *OAA* oxaloacetate; *6PG* 6-phosphogluconate; *KDPG* 2-keto-3-deoxy-6-phosphogluconate; *Ri5P* ribose 5-phosphate; *Ru5P* ribulose 5-phosphate; *X5P* xylose 5-phosphate; *S7P* sedoheptulose 7-phosphate; *E4P* erythrose 4-phosphate; *CO2_EX* extracellular carbon dioxide; *CO*_*2*_ intracellular carbon dioxide. The flux estimation yielded an excellent quality of fit for the considered mass isotopomers of amino acids, sugars, and sugar derivatives (Additional file 1: Table S1). The variance-weighted sum of squared residuals (SSR) was 377 and within the expected range (342–434) of the chi-square test at 95% confidence level, confirming also statistical acceptability (Additional file 1: Fig. S8). n = 4

Using the newly developed approach, *Synechocystis* 6803 was grown under photomixotrophic steady-state on [1-^13^C], [3-^13^C], [6-^13^C], and [^13^C_6_] glucose, respectively. The cyanobacterium consumed glucose and CO_2_ at specific rates of 0.42 mmol g^−1^ h^−1^ and 1.58 mmol g^−1^ h^−1^, respectively. Biomass was the only product, while organic by-products were not formed (Table 1). GC/MS analysis yielded 388 ^13^C mass isotopomers from the parallel setups (Additional file 1: Table S8). The labelling data were used, together with data on growth (Table 1) and cellular composition (Additional file 1: Table S2), to estimate the intracellular flux distribution of the microbe. All fluxes could be successfully determined at high precision (Fig. 4). With this, the GC–MS based approach delivered the first fully detailed flux distribution for photomixotrophic *Synechocystis* 6803. Overall, an excellent quality of fit was achieved (SSR = 377 and within the expected range (342; 434), passing the chi-square test at 95% confidence level) (Additional file 1: Fig. S8A). An extended data set, additionally considering 168 ^13^C positional amino acid enrichments from 1 and 2D NMR and thus 556 labelling information in total, provided the same intracellular fluxes (Additional file 1: Table S2, Fig. S9), while an excellent goodness of fit and a statistically valid estimation were achieved (Additional file 1: Table S3, Fig. S8B). The successful integration of the huge orthogonal ^13^C labelling data set from NMR and GC–MS greatly strengthened the confidence in the approach. Notably, the combination of NMR and GC–MS delivered flux estimates at increased precision (Additional file 1: Table S2).

**Table 1:** Kinetics and stoichiometry of photomixotrophic *Synechocystis* sp. PCC 6803 and selected single gene deletion mutants

Strain	Y_X/Glc_ (g mol^−1^)	µ (h^−1^)	q_Glc_ (mmol g^−1^ h^−1^)
Wildtype	183 ± 8	0.077 ± 0.002	0.421 ± 0.012
Δ*eda*	177 ± 8	0.076 ± 0.002	0.429 ± 0.017
Δ*pfkAB*	181 ± 8	0.077 ± 0.001	0.425 ± 0.011
Δ*xfp1/*Δ*xfp2*	177 ± 8	0.083 ± 0.002	0.468 ± 0.012

Glucose was used as carbon source. The data comprise the biomass yield on glucose (Y_X/Glc_), the specific growth rate (µ), and the specific glucose uptake rate (q_Glc_). (n = 3)

From the metabolic perspective, the influx of CO_2_ strongly increased the flux through the CBB pathway (Fig. 4). The CBB cycle flux (347.6%) surpassed the net uptake of CO_2_ from the outside (236.8%), indicating that CO_2_ was formed in metabolism via decarboxylating reactions. No flux through ED pathway toward 2-keto-3-desoxy-6-phosphogluconate (KDPG) was detected (0%). This was a big surprise, after it has been concluded that this pathway would serve as a previously overlooked glycolytic route in photomixotrophic *Synechocystis* 6803 [43]. We searched for KDPG, the key intermediate of the ED pathway, using ultrasensitive LC–MS/MS. In cell extracts of photomixotrophically growing *Synechocystis* 6803, however, the metabolite was not detected (< 0.02 nmol g^−1^). Glucose-grown *P. putida* KT2440, a well-known ED pathway user, taken as a control, contained a substantial amount KDPG (1.4 ± 0.2 µmol g^−1^), approximately fifty thousand-fold more than the threshold.

Also, no flux through the PK pathway was found. This observation was also unexpected, since this pathway metabolized up to 30% of the carbon in a mutant, derived from *Synechocystis* sp. PCC 6803, during photomixotrophic growth [44]. All in all, the microbe operated only a subset of its genetically available pathway repertoire at detectable levels.

### Glucose enters the CBB cycle via two parallel routes to maximize CO2 fixation

On a c-molar basis, glucose (68%), compared to CO_2_ (32%), provided the major fraction of carbon to build up the cell and drive metabolism. From a flux perspective, the sugar (except for a small amount being used for anabolism) exclusively fueled the CBB cycle and thereby maximized the fixation of CO_2_. At the G6P node, the sugar flux was distributed into the PGI shunt (63.1% phosphoglucoisomerase flux) and, to a lesser extent, into the OPP shunt (9.3% G6P dehydrogenase flux). The remaining amount of G6P was used to synthesize glycogen (Fig. 4). At the level of F6P, the glycolytic influx from G6P (63.1%) merged with a slightly higher CBB cycle flux from the carbon-three intermediate GAP (87.2%). GAP itself mainly supplied DHAP (176.9%) toward the formation of S7P via FBP aldolase and ended up in the regeneration phase of the CBB cycle via the action of transketolase (115.7%). Because the ED pathway was inactive, carbon-three intermediates were not generated from 6-phosphogluconate. Altogether, the microbe fueled a network of parallel routes that finally all supplied ribulose-1,5-bisphosphate, the CO_2_ acceptor, at high flux (347.6%) (Fig. 4). The CBB pathway and the PGI shunt were the dominant contributors of phosphorylated pentoses and provided more than 90% of them, while the OPP shunt formed the rest.

The carbon-three intermediate 3PG was the most strongly formed and consumed metabolite: with two molecules of 3PG being produced via the CO_2_ fixing reaction of the CBB, its turnover flux was as high as 695.2% (Fig. 4). Most of the 3PG pool, approximately 71%, was recycled back through gluconeogenesis to provide anabolic precursors in upper metabolism and (mainly) refuel the CBB cycle, as shown above. Hereby, the flux was distributed such that, supported by the glycolytic PGI shunt, GAP itself, DHAP, and F6P were each supplied at significant amount, perfectly matching the required stoichiometry of the regenerating part of the CBB cycle.

### The lower EMP pathway and the connected TCA cycle exclusively provide anabolic precursors

No extra carbon remained after all biosynthetic withdrawals, but the TCA cycle operated only for anabolic purposes and, therefore, did not contribute to the formation of redox power and energy (Fig. 4). This behavior appeared reasonable, as the light reaction of the operating photosynthesis supplied sufficient ATP and NADPH. Virtually no 2OG was left to be processed via succinic semialdehyde. In fact, 2OG provided the main carbon skeleton for nitrogen assimilation via the GS/GOGAT cycle at the intersection of carbon and nitrogen metabolism. The cellular level of 2-OG is sensed to regulate the PGAM reaction, which converts 3PG to 2PG and thereby adjusts the carbon flux from the CBB cycle either into the anabolic or catabolic direction [59].

### An ED pathway-deficient mutant exhibits perturbed fluxes at the intersection of the CBB cycle and glycolysis but no growth phenotype, when grown under photomixotrophic steady state

Given the surprising finding that the ED route did not carry flux, we studied a previously established *∆eda* mutant that lacked a functional ED pathway due to the deletion of KDPG aldolase [43]. The mutant revealed no growth phenotype under steady state photomixotrophic conditions (> 35 µE m^−2^ s^−1^, considering an imposed light intensity of 50 µE m^−2^ s^−1^ and cell concentrations below OD_750nm_ that absorbed less than 30% of the imposed light) (Table 1).

**Fig. 5:**
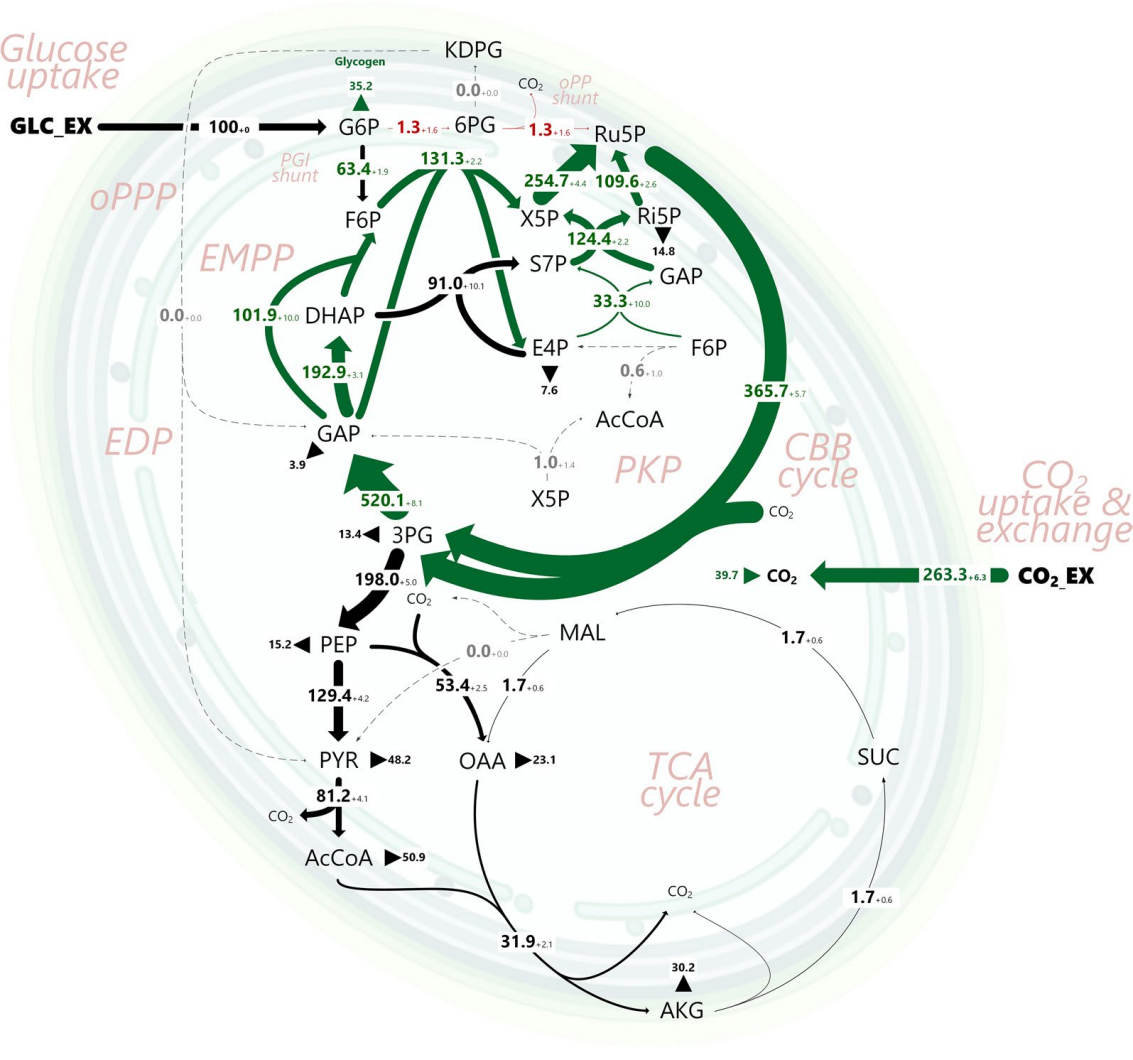
In vivo flux distribution of *Synechocystis* 6803 ∆*eda* during photomixotrophic growth on glucose and CO_2_ determined by GC–MS based ^13^C metabolic flux analysis. Fluxes are normalized to the glucose uptake (100%, 0.429 mmol g^−1^ h^−1^). The thickness of the arrows denotes the amount of flux. The errors for the fluxes reflect standard deviations, estimated by Monte-Carlo simulation. The anabolic fluxes into biomass are shown as triangles. The complete flux data set is given in Additional file 1: Table S2, where also the 95% confidence intervals from the Monte-Carlo analysis are provided. The abbreviations for the metabolites are explained in the legend of Fig. 4. The flux estimation yielded an excellent quality of fit for the considered mass isotopomers of amino acids, sugars, and sugar derivatives (Additional file 1: Table S3). The variance-weighted sum of squared residuals (SSR) was 377 and within the expected range (342–434) of the chi-square test at 95% confidence level, confirming also statistical acceptability (Additional file 1: Fig. S8). n = 4

Strikingly, strain *∆eda* re-directed its carbon fluxes to compensate for the deletion, when being analyzed using the novel GC–MS based approach (Fig. 5). Substantial changes were observed for the flux partitioning at the G6P node. The OPP shunt became almost inactive (1.3% versus 9.3%), while the anabolic flux from G6P into glycogen was increased (35.2% versus 27.6%). The latter was due to an increased glycogen content (252 mg g^−1^) in strain *∆eda*, approximately 27% higher than observed for the wildtype here (199 mg g^−1^) and previously [60]. The flux through PGI remained constant (63.1%). Notably, the CBB cycle flux was enhanced as compared to the wild type (365.7% versus 347.6%). This resulted in elevated supply of 3PG (514.9% versus 476.2%). Neither the wild type nor the *∆eda* mutant revealed EDA activity (< 0.01 mU mg^−1^).

At the 3PG node, however, the extra influx did not trigger an increased supply of metabolites of the lower glycolysis because the flux toward PEP (200.5% versus 198.0%) was unchanged. In fact, the extra carbon, arriving at the 3PG node, was exclusively cycled back to the regeneration phase of the CBB cycle. Hereby, the flux was distributed among the parallel routes that finally merged at Ru5P, including fine adjustments among pentose isomerases, transaldolase, and the two transketolases in the PP pathway. The TCA cycle was exclusively operated for anabolic purposes, and so were the anaplerotic reactions at the entry into the TCA cycle. It should be noted that the flux analysis of *Synechocystis* 6803 *∆eda*, like for the wild type, yielded an excellent quality of fit (Additional file 1: Table S4) and statistical validity for the best-fit solution (SSR = 394) (Additional file 1: Fig. S10A). The high precision regarding the estimated fluxes allowed clear discrimination between the wild type and the *∆eda* mutant. Because the relative glucose uptake rate was identical for both strains, all observed relative flux differences, described above, matched differences in absolute carbon fluxes. Notably, the flux changes in the mutant occurred, while no flux via the ED shunt was detectable in the wildtype. This indicates that Eda either possesses additional, yet undiscovered functions and/or that alternatively minor fluxes that are below detection limits in steady state conditions display regulatory functions, possibly by providing catalytic amounts of intermediates.

A *∆pfkAB* mutant, lacking the two phosphofructokinase genes [43], was not affected in growth (Table 1) but exhibited slight variations in intracellular fluxes (Additional file 1: Tables S2, S5). The same was true for a newly constructed *∆xfp1/∆xfp2* mutant (Additional file 1: Tables S2, S6). In both cases, the flux estimation was precise (Additional file 1: Table S2), the obtained fit very good (Additional file 1: Tables S5, S6) and the best-fit-solution was also statistically acceptable (SSR = 401 for *∆pfkAB*, SSR = 415 *Δxfp1/∆xfp2*) (Additional file 1: Fig. S10B, C).

### Strain ∆eda exhibits decreased fitness under fluctuating light

**Fig. 6:**
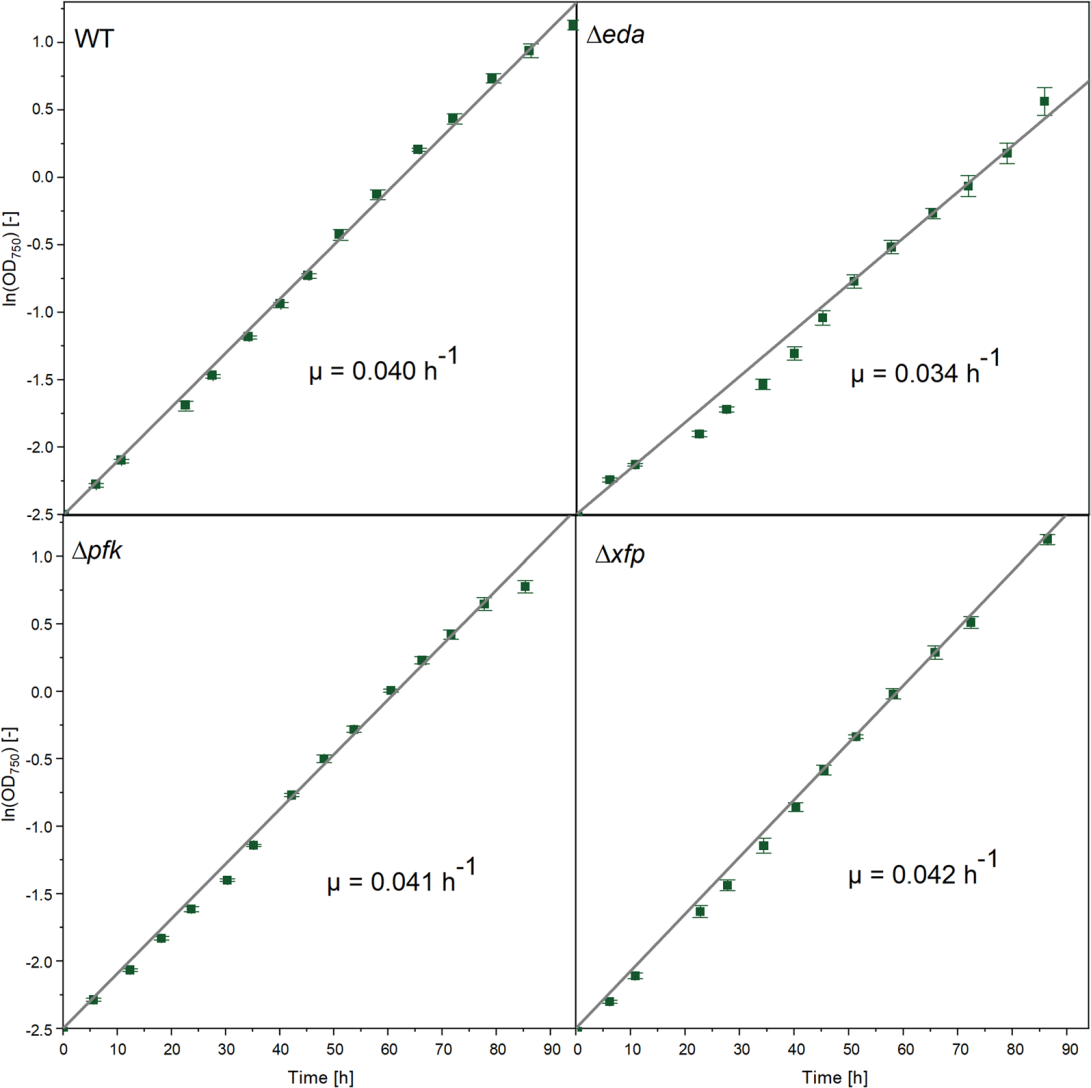
Photomixotrophic growth of *Synechocystis* 6803 wildtype and the single gene deletion mutants ∆*eda*, ∆*pfkAB,* and ∆*xfp1/xfp2* using glucose as carbon source. The cultures were illuminated using 1-min dark–light cycles. The statistical significance of differences in the specific growth rate was assessed by a t-test, considering a 95% confidence level (p < 0.01, **) and a 90% confidence level (p < 0.05, *). The analysis revealed that ∆*eda* (µ = 0.0339 ± 0.005 h^−^1, p = 0.003) and ∆*xfp1/xfp2* (µ = 0.0423 ± 0.003 h^−^1, p = 0.012) differed significantly from the wild type (µ = 0.0401 ± 0.003 h^−^1), whereas ∆*pfkAB* did not (µ = 0.0406 ± 0.003 h^−^1, p = 0.178). n = 3

As shown, pathway mutants showed no growth phenotype, when grown with consistently good illumination. We were curious, if the light regime would have an impact, and cultivated *Synechocystis* sp. PCC 6803 and the three deletion strains under fluctuating light. For this purpose, the illumination was changed from constant supply to automatically controlled 1-min intervals of light (50 µE m^−2^ s^−1^) and darkness. The wildtype was not affected in growth efficiency. The deletion strains *∆pfkAB* and *∆xfp1/∆xfp2* behaved like the wildtype and retained fast growth, the latter strain grew even slightly faster (Fig. 6). In contrast, the *∆eda* mutant revealed significantly decreased fitness: its specific growth rate was reduced by 15–20%.

### Deletion of the anabolic GAPDH2 renders glucose essential for growth in the light makes deletion of Eda impossible

The anabolic glyceraldehyde 3-phosphate dehydrogenase (GAPDH2), encoded by *gap2* (sll1342) is part of the regenerative part of the CBB cycle in the anabolic direction. As shown, this reaction carried the highest flux in the network (Fig. 4) and was even activated in the *∆eda* mutant (Fig. 5). A GAPDH2 deletion strain was constructed in this work to get a first impression on the impact of the enzyme. It was unable to grow under photoautotrophic conditions (Table 2). This was to be expected as the regeneration of the acceptor for CO_2_ should be difficult in this mutant. Under photomixotrophic conditions, i.e., upon addition of glucose in the light, growth of the mutant was (partly) rescued, however, with a significantly impaired growth rate in comparison to the wildtype. This observation confirmed that the supply of carbon via GAPDH2 was crucial to replenish and operate the CBB cycle at full rate. On the other hand, it showed the importance of the glycolytic PGI and OPP shunts which seemingly could serve as a backup to fuel the CBB cycle under photomixotrophic conditions. Notably, it was impossible (four independent trials) to delete *eda* in the *Δgap2* mutant under photoautotrophic or photomixotrophic conditions as the resultant mutants were not viable. It is thus nearby to assume that the ED shunt might gain importance in this genetic background and might supply GAP which can no longer be provided by GAPDH2. Further analysis of the growth phenotypes revealed that strain *Δgap2* was able to grow faster under photomixotrophic conditions than the wildtype under photoautotrophic conditions and *Δgap2* under heterotrophic conditions (Table 2). Since photomixotrophic growth of *Δgap2* was faster than its heterotrophic growth, it can be deduced that the observable efficient photomixotrophic growth was the result of a synergistic metabolism, in which glucose fuelled the CBB cycle and thereby enhanced CO_2_ fixation, as shown by the flux analyses (Fig. 4). It appears interesting to study this mutant in more detail in the future, including also studies on the metabolic flux level and a possible activation of the ED shunt.

**Table 2:** Impact of deleting the anabolic glyceraldehyde 3-phosphate dehydrogenase (GAPDH2) on the growth of *Synechocystis* sp. PCC 6803

Strain	Photoautotrophic growth (h^−1^)	Photomixotrophic growth (h^−1^)	Heterotrophic growth (h^−1^)
Wildtype	0.012 ± 0.003	0.077 ± 0.002	0.001 ± 0.001
Δ*gap2*	No growth	0.033 ± 0.009	0.002 ± 0.001

The data show the specific growth rate in the wildtype and a *∆gap2* mutant under photoautotrophic, photomixotrophic, heterotrophic conditions. Glucose was used as carbon source for the latter two conditions. (n = 3)

## Discussion

### The developed approach enables 13C metabolic flux analysis of photomixotrophic cyanobacteria at high resolution and accuracy

As shown, our approach enables precise and accurate metabolic flux studies of photomixotrophic cyanobacteria. Photomixotrophic metabolism is particularly promising for the future commercialization of cyanobacteria because the co-utilization of CO_2_ and organic carbon provides additional building blocks and energy. This mode enables enhanced production and higher cell density [61] and confers industrial feasibility [62]. Remarkably, photomixotrophic production has increased titers up to five-fold over traditional autotrophic conditions [63]. To further exploit this growth mode, recent efforts have even installed photomixotrophy in cyanobacteria that are obligate photoautotrophic [64]. Therefore, our approach displays a valuable tool to further characterize and develop cyanobacterial cell factories. As example, it allows to study the pathways of genetically engineered mutants in vivo which, as example, provide higher levels of acetyl-CoA [61] or exhibit enhanced CO_2_ fixation and butanediol production [62]. In addition, flux studies could shed light on the mixotrophic use of alternative carbon sources, including glycerol, acetic acid [65], and xylose [66], toward more sustainable bioproduction. Finally, cyanobacteria include about 2000 species in 150 genera, with a wide range cellular structures and physiological strategies. Many of them are capable of photomixotrophic growth, including members of biotechnological relevance and impact [67].

Our approach renders these interesting microbes more easily accessible on the flux level. Using GC–MS data alone as labelling input, it offers low experimental effort because the instrument is easy to use and does not even have to be adapted to handle the different analytes for labelling measurement. Using both, NMR and GC–MS data might help in situations, were maximum precision and determinability is needed. In addition, it promises additional value, if the network gets even more complex, e.g., due to the incorporation of additional heterologous pathways.

### No flux through the ED pathway is detectable in photomixotrophic cells under metabolic steady state

The ED pathway has been discovered in various microbes as part of the glycolytic network, where it provided enhanced robustness and metabolic flexibility [14, 68]. Recently, it was concluded that the ED pathway would serve as a previously overlooked glycolytic route in photomixotrophic *Synechocystis* 6803 [43]. However, *Synechocystis* 6803 *∆eda*, lacking a functional ED pathway, grew like the wildtype under photomixotrophic conditions with constant light.

This observation alone did not allow a clear conclusion on the contribution of the ED route in vivo. *Synechocystis* sp. PCC 6803 possessed an arsenal of alternative routes in its central carbon metabolism to potentially bypass the ED pathway (Fig. 1). Notably, microbes are well known to compensate the knockout of a pathway by redirecting flux through such alternative routes or redundant enzymes, often without a notifiable growth phenotype. As example, such observations have been made in yeast [69, 70] and bacteria [53, 71].

Our results, however, surprisingly demonstrate that the ED pathway in vivo does not contribute substantial glycolytic flux to glucose breakdown in photomixotrophic *Synechocystis* cells. Likewise, also the PK pathway, phosphofructokinase and malic enzyme appeared dispensable. Under the metabolic steady-state conditions used here, glucose (except for a small fraction used in anabolism) was exclusively fed via the PGI and the oxidative OPP shunts into the CBB cycle. Stoichiometrically, this flux pattern maximized the fixation of CO_2_ (Fig. 4) and can be regarded optimal about growth efficiency, an important performance indicator when applying cyanobacteria as cell factories.

On a first glance, the lack of detectable fluxes via the ED route in vivo seems to contradict the previously assumed picture [43] but a closer inspection of the experimental conditions suggests that the supply with light might render the ED route active or inactive. Here, the microbe was grown under continuous light. In contrast, the previous setup, a several centimeter thick glass tube, was well illuminated only in outer zones, whereas the core of the culture (covering up to 90% of the volume) was darkened (Additional file 1: Fig. S11). Cells traveling across the tube, obviously faced dark–light cycles. When mimicking this setup by fluctuating light supply (Fig. 6), we could interestingly “activate” the limited growth phenotype of the *∆eda* mutant, observed before under photomixotrophic conditions [43]. It appears possible that the ED pathway gets activated when cells face changing light [43, 72]. Naturally, cyanobacteria frequently face changing light, including fluctuations across time and gradients across space. These changes also occur during cyanobacterial chemical production in bioreactors. Clearly, the microbes must be well prepared to handle such changes.

Mutants in which either the OPP shunt or *eda* were deleted, display a delayed reactivation of the CBB cycle when transferred from darkness to light [72]. It was put forward that the CBB cycle is either replenished via the OPP shunt or a combination of the PGI and ED shunt at the onset of light. Glycogen-derived G6P is decarboxylated via the OPP shunt and directly yields ribulose 5P. However, the PGI shunt yields fructose-6P. To replenish the CBB cycle, two molecules of F6P must be combined with one molecule of GAP (DHAP). Under photomixotrophic steady state conditions, there is a substantial flux via the PGI shunt which merges with GAP (DHAP) from the CBB cycle provided by GAPDH2 (Fig. 4). The CBB cycle is regulatory tightly connected to the light reaction of photosynthesis. Fluctuating light conditions that perturb photosynthesis and the CBB cycle might transiently cut back the supply of GAP via GAPDH2 from the CBB cycle. It is likely that this is the condition under which the ED shunt gets activated and supplies GAP to be combined with two molecules of F6P from the PGI shunt. In line with this, we were unable to delete *eda* in a *∆gap2* mutant which lacks GAPDH2. This combination plays apparently no or only a minor role under steady state conditions, if the CBB cycle operates smoothly. However, it is highly remarkable, that the OPP shunt is substantially downregulated in *∆eda.* One possible and obvious explanation might be that there is indeed a very small flux via the ED shunt also under steady state conditions (not detectable here), which might result in the accumulation of KDPG, which again remains below detection limits but regulates the first enzyme of the OPP shunt G6P dehydrogenase (*zwf*) as shown for *P. putida* [73]. This kind of regulation might require only catalytic amounts of KDPG. This idea requires further experimental examinations. However, the downregulation of the OPP shunt in *∆eda* obviously impedes the injection of glucose into the CBB cycle, which results in the formation of higher amounts of glycogen in the mutant and furthermore accelerates CO_2_ fixation by enhanced CBB cycle fluxes. It is due to these enhanced CBB cycle fluxes, that the photomixotrophic growth of *∆eda* can keep up with the growth of the WT even though it forms higher amounts of glycogen. However, as soon as conditions get more natural by fluctuating light, *Synechocystis* obviously requires a higher metabolic flexibility and additional glycolytic shunts to counteract these perturbations and keep high growth rates.

Under photoautotrophic conditions, glycolytic routes form as well shortened glycolytic shunts that replenish and fine-tune the Calvin-Benson-Bassham (CBB) cycle as anaplerotic reactions to support transitions from darkness to light [72]. The idea that the OPP shunt replenishes the CBB cycle under rapidly changing conditions has been hypothesized for plants as well [74]. Cyanobacteria and plants might switch between different glycolytic shunts in order to balance redox pools that might be perturbed in fluctuating light in photosynthesis. Such a function could explain the growth defect of strain *∆eda* under rapidly oscillating light. Performing a time-averaged flux analysis under conditions of rapidly oscillating light might help this hypothesis to be tested in the future.

The idea that the OPP shunt replenishes the CBB cycle under rapidly changing conditions has been hypothesized for plants [74]. Such a function could explain the growth defect of strain *∆eda* under rapidly oscillating light. Performing a time-averaged flux analysis under conditions of rapidly oscillating light might help this hypothesis to be tested in the future.

Apart from a potential minor flux via the ED pathway under steady state, which stays below detection limits but might suffice to provide minor amounts of GAP and furthermore regulates the OPP shunt via catalytic amounts of KDPG (which are again below detection limits), EDA might as well have additional functions, that were not described yet. As example, EDA is a known multifunctional enzyme in *E. coli*, conferring activity as 2-keto-4-hydroxyglutarate aldolase and oxaloacetate decarboxylase in addition to its contribution to the ED pathway [75] but more work is needed to resolve this picture. Dynamic studies that integrate fluxomics with proteomics, transcriptomics, as well as protein–protein interaction studies, seem promising toward a multi-level insight [7].

### Photomixotrophic growth flexibly integrates light and nutrient availability by adapted intracellular flux distributions

So far, the outcome of photomixotrophic flux studies is quite diverse. As example, substantial flux through malic enzyme has been reported in earlier studies [76] whereas we observed that the enzyme was inactive (Fig. 4). Likewise, the contribution of the OPP pathway greatly differs. In photomixotrophic *Synechocystis*, the OPP pathway flux was found to be absent [76], low (9%) (this work), and high (70%) [56]. Notably, photomixotrophic steady state growth in these studies differed in light and CO_2_ supply. Here (this work) and before [43, 72], CO_2_ was supplied from the gas phase at atmospheric level, mimicking natural availability. In contrast, other setups supplied elevated amounts of NaHCO_3_ (2 g L^−1^) under a sealed atmosphere, leading to a markedly increased CO_2_ level [77]. Obviously, this boosted CO_2_ uptake over glucose uptake, leading to a molar uptake ratio of 6:1 between CO_2_ and the sugar; in this work using ambient CO_2_ the ratio was only 2:1. In another flux study, CO_2_ was even quantitatively removed from the aeration stream so that the cells could only fix CO_2_ that stemmed from glucose oxidation, while the light supply (125 µE m^−2^ s^−1^) was more than twice as high [76]. It seems plausible that these differences affect pathway fluxes.

It was suggested for cyanobacteria that the regeneration of pyruvate from malate may become beneficial when cells enter into a state, in which NADPH needs to be replenished [76]. Photomixotrophy, offering the ability to generate ATP and NAPDH by using light, however, does not appear to trigger NADPH limitation. Under these conditions, the cells even maintain the ATP and NADPH costly recycling of GAP to refuel of the CBB cycle at very high flux (Fig. 4). In this regard it appears that the flux through the OPP shunt (generating less than 10% of the total NADPH) presumably contributed to a coordinated fueling of the CBB cycle, rather than to supply redox power, which would be the case for heterotrophic microbes. The picture could be different for conditions of limiting light, since a dependency of NADPH regeneration on light is known [78]. Here, we assured that cells were constantly supplied with sufficient light, making supply of NADPH from decarboxylating reactions suboptimal due to the loss of carbon. Notably, (under photoautotrophic conditions) the C_4_ shunt via malic enzyme responds to light supply [79]. Furthermore, also the OPP pathway flux is affected by the inhibition of photosynthesis [56]. Beneficially, our approach now enables to study these effects more systematically in the future, and these data promise to contribute to a better understanding of the lifestyle of cyanobacteria.

## Conclusions

The ^13^C metabolic flux approach developed in this work, allows to fully determine all fluxes in the complex core carbon network of photomixotrophic *Synechocystis*. The approach requires only GC/MS for ^13^C analysis and therefore rather little experimental effort. Given the impact of flux analysis to derive systems understanding of microbes and breed superior cell factories [5, 22], our development provides a useful technology for future research of photomixotrophic cyanobacteria, such as *Synechocystis*, *Synechococcus* sp. [66] and *Spirulina* sp. [46], as well as photomixotrophic microalgae such as *Chlorella vulgaris* [48] and other species [80].

Glucose almost exclusively fueled the CBB cycle and maximized CO_2_ fixation during mixotrophic growth of *Synechocystis* 6863. In this optimum mode, only the CBB cycle operated in its entirety, whereas the glycolytic routes formed glycolytic shunts that fueled the CBB cycle. The PGI shunt was the most prominent glycolytic shunt, followed by the OPP shunt. No flux through the ED shunt and the PK was detectable. However, an ED pathway-deficient mutant revealed a strongly decreased OPP flux, while the CBB cycle flux was enhanced, and glycogen formation was increased. When exposed to photomixotrophic conditions under fluctuating light, this mutant exhibited decreased fitness. It was furthermore impossible to delete Eda in a GAPDH2 deletion mutant. This indicates that minor regulatory fluxes via the ED pathway might exists that provide minor amounts of GAP for the regeneration phase of the CBB cycle and might furthermore regulate the activity of the OPP shunt possibly by catalytic amounts of KDPG. However, this view requires further experimental studies.

## Materials and methods

### Strains

*Synechocystis* sp. PCC 6803 was obtained from the Pasteur Culture Collection of Cyanobacteria (PCC, Paris, France). A *∆eda* mutant, lacking 2-keto-3-deoxy-6-phosphogluconate aldolase (*eda*, EC 4.1.2.14, sll0107) was obtained from previous work [43]. A *∆pfkAB* double deletion strain, lacking the two phosphofructokinase isoenzymes PfkA and PfkB (*pfk*A, EC 2.7.1.11, sll0745; *pfk*B, EC 2.7.1.90, sll0745) was taken from the same study [43]. *P. putida* KT2440 was taken from previous work [81].

### Genetic engineering

A double deletion mutant, lacking the two phosphoketolase isoenzymes (*xfp1*, slr0453; *xfp2*, sll0529) was constructed in two subsequent steps using the work flow described before [43]. In short, deletion constructs were assembled from a kanamycin resistance cassette and from 200 bp long flanking regions up- and down-stream of the target gene using Gibson assembly and then cloned into pBluescript. *Synechocystis* sp. PCC 6803 was then transformed with the plasmid. Selected mutants were checked via PCR for segregation. Using the same approach, the gene for the CBB cycle isoform of the glyceraldehyde-3-phosphate dehydrogenase (GAPDH2) was knocked out in *Synechocystis*. This was accomplished by replacing the *gap2* gene (sll1342) of WT with a kanamycin resistance cassette. *Synechocystis* sp. PCC 6803 was transformed with the constructed plasmid. The mutants were finally checked via Southern blotting for segregation and the correct genotype (Additional file 1: Figs. S12, S13). The corresponding primers are listed in Additional file 1: Table S7.

### Southern-blotting

Two hundred ng genomic DNA was digested with Hind III and loaded on a 0.8% agarose gel in TBE buffer. After blotting the DNA on a nylon membrane (Hybond N +, Merck, Darmstadt, Germany) it was cross-linked to the membrane in a Stratalinker (Stratagene, CA, USA). Detection of the respective bands was carried out by the Dig DNA labeling and detection kit (Roche, Penzberg, Germany) according to the manufacturer’s instructions.

### Medium

One liter of liquid BG11 medium [82] contained 10 mM of glucose, 1.50 g of NaNO_3_, 69 mg of MgSO_2_* 6H_2_O, 36 mg of CaCl_2_* 2H_2_O, 6 mg of citric acid, 10.4 mg of Na_2_EDTA, 2.86 mg of H_2_BO_3_, 1.81 g of MnCl_2_* 4H_2_O, 0.22 mg of ZnSO_4_* 7H_2_O, 0.39 mg of NaMoO_4_* 2H_2_O, 0.079 mg of CuSO_4_* 5H_2_O, 0.0494 mg of Co(NO_3_)_2_* 6H_2_O, 4 mg of K_2_HPO_4_, 2 mg of Na_2_CO_3_, 6 mg of ammonium ferric citrate, and 5 mL of 50 mM TES (pH 8.0). For plate cultures, the medium was amended with 15 g L^−1^ of agar (Becton Dickinson, Franklin Lakes, NJ, USA) and 1 mM of thiosulfate.

### Medium for ^13^C flux analysis

For isotopic ^13^C tracer studies, naturally labelled glucose was replaced either by (i) 99% [1-^13^C] glucose (Sigma-Aldrich, Steinheim, Germany), (ii) 99% [3-^13^C] glucose (Cambridge Isotope Laboratories, Tewksbury, MA, USA), (iii) 99% [6-^13^C] glucose (Omicron Biochemicals, Southbend, IN, USA), and (iv) 99.9% [^13^C_6_] glucose (Eurisotop, Saarbrücken, Germany), respectively.

### Physiological studies in shake flasks

Generally, cells were collected from a 5-day old plate culture (28 °C, 50 µE m^2^ s^−1^) and resuspended in 1 mL fresh medium. An appropriate amount of this suspension was used to inoculate a pre-culture to an initial cell concentration of OD_750_ = 0.1. For photomixotrophic growth studies, the pre-culture was then incubated under light in 250 mL baffled shake flasks (10% filling volume, covered with translucent caps for improved illumination) on a rotary shaker (28 °C, 100 rpm, Infors HT Multitron, Infors, Basel, Switzerland). The in-built illumination panel at the ceiling of the incubator provided LED warm white light (50 µE m^2^ s^−1^) with an even distribution across the tray (± 6%). The translucent caps were derived from aluminum caps, routinely used in microbial cultivation for contamination-free gas exchange with the surrounding ambient atmosphere. They were constructed as follows. The aluminum caps were cut out in a circular shape so that the top cap had a round hole about 3 cm in diameter. The hole in each cap was then covered by a translucent foil (PCR adhesive film, Axon Labortechnik, Kaiserslautern, Germany) which was glued to the outer aluminum ring. During the exponential growth phase, cells were collected (8000×*g*, 3 min, 4 °C), washed, and used to inoculate the main culture to an initial cell concentration of OD_750_ = 0.1, which was then incubated as described above. In addition, heterotrophic growth studies were conducted, using the same set-up, except that all cultures were conducted in the dark. (n = 4). Growth studies of *∆gap2* were performed as previously described [43]. Specific growth rates were calculated during exponential growth, taking the slope of the logarithmic increase of cell concentration over time (Fig. 5).

### Isotopic tracer studies

The cultures for ^13^C tracer experiments were grown as described above, except that 50 mL baffled shake flasks with 5 mL medium were used. An initial comparison showed that the smaller volume did not affect the growth physiology (data not shown). The pre-culture and the main culture medium were conducted on the same isotopic glucose tracer to ensure that the non-labelled inoculum, initially obtained from a plate culture, was below 1% of the cells, finally harvested slightly above OD_750_ = 1, so that its impact on the later labelling measurement was negligible [54]. Each glucose isoform was treated individually (n = 4).

### Light intensity measurement

Light intensity measurement was carried out using an illuminometer (Tack Life LM01, Shenzen Temine Technology, Guandong, China).

### Quantification of cell concentration

Cell growth was monitored spectrophotometrically at 750 nm. Hereby, culture samples with higher cell densities were diluted with medium to optical densities below 0.3 to ensure a liner correlation between cell concentration and optical density reading. In addition, cell dry mass (CDM) was quantified by gravimetric analysis. Cells were collected by filtration (0.2 μm, regenerated cellulose, 47 mm, Sartorius, Göttingen, Germany), washed once with deionized water, and dried at 80 °C until constant weight. The obtained correlation factor was CDM [g L^−1^] = 0.261 × OD_750_.

### Quantification of glucose

Glucose was quantified in culture supernatant using a biochemical analyzer (Biochemical Analyzer 2950D, YSI, Yellow Springs, Ohio, USA).

### Quantification of intracellular glycogen

Glycogen was quantified as monomeric sugar after enzymatic digest of the polymer [83]. In short, cells were harvested by centrifugation (9000×*g*, 5 min, 4 °C) and lysed at 95 °C for 2 h in 300 µL 30% KOH. Glycogen was then precipitated by addition of 900 µL absolute ethanol, incubation over night at − 20 °C, and subsequent centrifugation (10,000×*g*, 5 min, 4 °C). After drying for 20 min at 50 °C the resulting pellet was dissolved in 500 µL reaction buffer (100 mM sodium acetate, adjusted to pH 4.5) and subjected to digestion with amyloglucosidase (1.5 mg mL^−1^, 90 min, 60 °C). After centrifugation (10,000×*g*, 10 min, RT), the level of glucose in the supernatant was quantified spectrophotometrically (340 nm) using an enzymatic assay, coupling glucose conversion to the formation of NADPH [83].

### GC–MS labelling analysis of proteinogenic amino acids

Cells (2 mg CDM) were hydrolyzed (250 µL, 6 M HCl, 18 h, 100 °C). Cell debris was then removed from the hydrolysate by filtration (0.2 μm, Ultrafree-MC, Merck Millipore, Darmstadt, Germany). Subsequently, the hydrolysate was dried under nitrogen. The obtained pellet was re-dissolved in 50 µL N,N-dimethylformamide [1% (v/v) in pyridine]. The contained amino acids were converted into the corresponding *t*-butyl-dimethyl-silyl derivatives by the addition of 50 µL N-methyl-*t*-butyldimethylsilyl-trifluoroacetamide (MBDSTFA, Macherey–Nagel, Düren, Germany) and incubation for 30 min at 80 °C [22]. The mass isotopomer distributions of the derivatized amino acids were then determined using GC–MS analysis (Agilent 7890A, Quadrupole Mass Selective Detector 5975C, Agilent Technologies, Santa Clara, California, USA) equipped with an HP-5MS column as stationary phase (30 m, 250 × 0.25 μm, Agilent Technologies), and helium (5.0) as mobile phase (1.7 mL min^−1^). The following temperature gradient was used: 0–2 min, 120 °C; 2–12 min, 8 °C min^−1^; 12–24.5 min, 10 °C min^−1^; 24.5–27 min, 325 °C. Further temperature settings controlled the inlet (250 °C), the transfer liner (280 °C), the ion source (230 °C), and the quadrupole temperature (150 °C).

The mass isotopomer distributions of 19 amino acid fragments, previously proven informative for ^13^C metabolic flux analysis with *Synechocystis* sp. PCC 6803 [57] were found of appropriate quality to be considered for flux estimation: l-alanine (*m/z* 260, *m/z* 232), glycine (*m/z* 218, *m/z* 246), l-valine (*m/z* 260, *m/z* 288), l-leucine (*m/z* 274), l-isoleucine (*m/z* 274), l-serine (*m/z* 390, *m/z* 362), l-threonine (*m/z* 404, *m/z* 376), l-phenylalanine (*m/z* 336), l-aspartate (*m/z* 418, *m/z* 390), l-glutamate (*m/z* 432), and l-arginine (*m/z* 442). l-Lysine, l-histidine, and l-tyrosine yielded signals of low intensity and ambiguous quality, so that they were not considered further. Other amino acids (l-glutamine, l-asparagine, l-cysteine, l-methionine and l-tryptophan) were not available due to chemical decomposition during the hydrolysis process [54]. The ^13^C labelling pattern of each ion fragment was first evaluated in scan mode to exclude isobaric interference with the matrix. Subsequently, its mass isotopomer distribution was measured in duplicate using singe ion monitoring mode [22].

### GC–MS labelling analysis of sugars

For the derivatization of biomass-derived sugars, silylation was applied according to previous work [9]. In short, 2 mg CDM was hydrolyzed (2 h, 100 °C, 250 µL 2 M HCl). Cell debris was removed by filtration (0.2 μm, Ultrafree-MC, Merck Millipore, Germany). The obtained hydrolysate was dried under nitrogen, dissolved in 100 µL methoxylamine (2% (v/v) in pyridine), and incubated at 80 °C for 1 h to yield the oximated sugar derivatives, which were then further converted into the corresponding silylated form, using a 30 min incubation at 80 °C with 50 µL N-Methyl-N-(trimethylsilyl) trifluoroacetamide (MSTFA, Macherey–Nagel, Germany). The mass isotopomer distributions of the derivatized sugars were determined using GC–MS (Agilent 7890A, Quadrupole Mass Selective Detector 5975C, Agilent Technologies), equipped with an HP-5MS column as stationary phase (30 m, 250 × 0.25 μm, Agilent Technologies) and helium (5.0) as mobile phase (1.7 mL min^−1^). The following temperature gradient was used: 0 min, 100 °C; 0–26 min, 5 °C min^−1^; 26–29.8 min, 25 °C min^−1^. Further settings controlled the inlet (250 °C), the transfer liner (280 °C), the ion source (230 °C), and the quadrupole temperature (150 °C). The analysis yielded clean signal for the following ion clusters: ribose (*m/z* 307), glucose (*m/z* 319) and glucosamine (*m/z* 319). The ^13^C labelling pattern of each fragment was first evaluated in scan mode to exclude isobaric interference with the matrix and then measured in singe ion monitoring mode as duplicate.

### NMR measurement

Collected biomass (5 mg) was resuspended in 6 M HCl and hydrolyzed for 12 h at 110 °C. The obtained hydrolysate was dried and washed twice with D_2_O (dried again between each washing step), resuspended in 200 μL DCl [0.1% (v/v) in D_2_O], and transferred into 3 mm NMR tubes for analysis. The biomass hydrolysates were analyzed in duplicate by 1D ^1^H NMR, 1D ^13^C NMR, 2D ZQF-TOCSY NMR and 2D HSQC [21]. For the analysis, a spectrometer Bruker Ascend™ 800 MHz (Bruker, Billerica, Massachusetts, USA) equipped with a 5 mm CQPI (^1^H, ^13^C, ^31^P, ^15^N) cryoprobe and a Sample Jet auto sampler (Bruker, Billerica, Massachusetts, USA) was used.

### Metabolic network of photomixotrophic *Synechocystis* sp. PCC 6803

A stoichiometric network model of the central metabolic pathways of *Synechocystis* sp. PCC 6803 was created, using data from genome annotation [84]. Afterwards, the model was curated and extended, based on recent findings (Fig. 1). It comprised the CBB cycle, the EMP pathway (including the PGI shunt), the OPP pathway (including the OPP shunt), the ED pathway [43], the TCA cycle with succinate semialdehyde as intermediate [45], the PK pathway [44], and the entire set of reactions at the pyruvate/phosphoenolpyruvate node. The anabolic pathways to biomass were adapted from previous work [60], including more recent amendments [85]. For strain *∆eda*, the biomass composition considered the slightly increased glycogen formation, previously found under photoautotrophic conditions [72] and experimentally determined for mixotrophic growth here (Additional file 1: Table S8). No organic by-product was detected in any of the cultures so that no further side reaction was included.

### Metabolic flux estimation and statistical evaluation

The network for photomixotrophic growth was implemented into the software OpenFLUX, including a specification of the underlying carbon transitions [86]. The reaction list is given in Additional file 1: Table S9. The software integrated labelling data of different origin: mass isotopomer distributions from GC–MS measurement and position specific isotopic enrichments from NMR analysis. Experimental GC–MS data were corrected for the natural abundance of isotopes [87]. NMR data did not require such a correction. For the integrated analysis of parallel tracer experiments, all data were fitted simultaneously to the same flux model as described before [22].

For flux calculation, the variance-weighted sum of squared residuals (SSR) deviation between measured and simulated mass isotopomer distributions, positional enrichments, and rates was minimized. The result, obtained at minimized deviation, was considered best to represent the in vivo fluxes. Experimental errors were implemented using the pre-solver function of OpenFLUX. Anabolic fluxes into biomass were varied by ± 15% to reflect experimental noise. The considered measurement error for GC–MS ^13^C analysis of mass isotopomers was ± 0.006 (amino acids) and ± 0.01 (sugars). For the case, where NMR and GC–MS data were combined to estimate the fluxes, the absolute error for the analysis of positional ^13^C enrichments by NMR was ± 0.025. The model exhibited limited capacity to precisely estimate the cyclic flux through the oxaloacetate-malate-pyruvate node. To overcome this limitation, in vitro activity analysis of malic enzyme in the strains was used as a constraint as described previously [22]. The enzyme was found not expressed (< 0.01 mU mg^−1^), so that the corresponding flux was set to zero which enabled a robust determination of these fluxes. The flux through genetically eliminated reactions was set to zero, when analyzing deletion strains. Because the non-linear structure of isotopomer models potentially leads to local minima, 250 parameter estimations with random initial starting points were performed. These yielded all the same solution which verified that the acquired data were fully descriptive and that the determined flux distribution displayed the global minimum [9]. To determine the goodness-of-fit, the SSR of the best-fit result was subjected to a chi-square test at 95% confidence level [88]. Finally, 95% confidence intervals for all individual fluxes were calculated using Monte-Carlo analysis with 250 iterations.

### Quantification of KDPG

Metabolite extraction and sampling was performed as described previously [89]. Prior to extraction, cells were quenched in − 20 °C buffer [25 mM formic acid, 95% (v/v) acetonitrile in water] at a volume ratio of 1–3. The quenched mixture was immediately frozen using liquid nitrogen, followed by freeze drying. The dried extract was resuspended in 500 µL of 5 mM ammonium carbonate (pH 9.2) and clarified (0.2 μm, Ultrafree-MC, Merck Millipore). The obtained eluate was used for analysis. Subsequent analysis was conducted on an LC–ESI–MS/MS (QTRAP 6500^+^, AB Sciex, Darmstadt, Germany), using a pHILIC column (SeQuant ZIC-pHILIC 5 µm, 150 × 2.1 mm, Merk, Darmstadt, Germany) as stationary phase with a flow rate of 0.3 mL min^−1^ and column temperature of 25 °C with the following gradient of eluent A (5 mM ammonium carbonate, pH 9.2) and eluent B (acetonitrile): 0 min 80% eluent B, 20 min shift to 20% eluent of B, 21–25 min shift to 5% eluent B, 28–30 min shift to 80% eluent B. The MS was adjusted to optimized settings, acquired for a KDPG standard (M1 = 257, M2 = 97, DP = − 4.64 kV, CE = − 20.63 kV, CXP = − 9.9 kV).

### Enzyme assays

Cells were harvested by centrifugation (8000×*g*, 3 min, 4 °C), washed twice (100 mM Tris–HCl, pH 7.8), and resuspended (100 mM Tris–HCl, 10 mM dithiothreitol). After addition of 0.1 mm silica beads (Lysing Matrix B, MP Biomedicals, Santa Ana, CA, USA), the cells were disrupted (3 × 30 s, 6000 s^−1^, Precellys, PeqLab, Erlangen, Germany) with 30 s cooling breaks on ice in between. Cell debris was removed by centrifugation (8000×*g*, 3 min, 4 °C). The obtained cell extract was then used for enzyme activity measurement. In all cases, the protein concentration in the cell extract was measured using a colorimetric kit (Pierce BCA Protein Assay Kit, Thermo Fisher, Rockford, IL, USA), to derive specific enzyme activity.

KDPG aldolase was assayed by coupling its activity to that of lactate dehydrogenase [43]. The reaction mix (1 mL) contained 100 mM Tris–HCl (pH 7.8), 1 mM NADH, 5 mM MgCl_2_, 20 mM KDPG, 10 U lactate dehydrogenase and 100 µL cell extract. Enzyme activity was monitored at 28 °C via the change of absorbance at 340 nm. Malic enzyme activity was assayed using a reaction mix (1 mL) that contained 100 mM Tris–HCl (pH 7.8), 1 mM NADP^+^, 40 mM sodium malate, 5 mM MgCl_2_, and 100 µL cell extract [22]. In a parallel set-up, NADP^+^ was replaced by an equimolar amount of NAD^+^. Enzyme activity was monitored at 28 °C via the change of absorbance at 340 nm.

## Supplementary Information

**Additional file 1: Figure S1.** Development of a suitable workflow for photomixotrophic growth of *Synechocystis *6803 in ^13^C metabolic flux analysis. Cultivation profile with indicated threshold for growth with sufficient light supply, **A** estimation of the maximal cell concentration that provides sufficient light for maximum growth, **B** light absorption of cultures, incubated in BG11 medium, at varied cell concentration and depth, the values are normalized to 100% for the maximum illuminance, **C** modelling of the relative light intensity as function of cell concentration and light passage (depth) using the Lambert–Beer law, **D** the orange lines indicate that a culture at OD = 2 and the light path length for the conducted shake flask cultures (42 mm) receives 70% of the supplied illumination, while absorbing the remaining 30%. n = 3.** Figure S2.** Time profile of ^13^C amino acid labelling patterns (given at different cell concentrations) during photomixotrophic cultivation of *Synechocystis *6803, grown on [1-^13^C] glucose (left) and [U-^13^C] glucose (right). **Figure S3. **Computational evaluation of previous approaches, based on single isotope experiments and (mainly) GC/MS analysis of proteinogenic amino acids, for ^13^C metabolic flux analysis of photomixotrophic *Synechocystis *6803. The tested approaches had been applied for photomixotrophic flux analysis of *Synechocystis*, before the full network was known [44] or had focused on a subnetwork of the microbe [45]. A third approach had been derived to analyze heterotrophic *Synechocystis *sp. PCC6803 without an active CBB cycle [55]. There, the approaches analyzed for the achievable precision and accuracy to determine fluxes in a scenario with 0% (left), 5% (middle), and 50% (right) flux through the ED and the PK pathways. The show the outcome of a Monte-Carlo simulation that mimicked 100 repetitions of the corresponding flux study while taking experimental errors into account. Displayed are key fluxes of upper and lower carbon metabolism, i.e., through ED, PP, EMP, and PK pathways, CBB cycle, and TCA cycle, are shown. The color indicates the determinability of a flux parameter. The color indicates flux determinability: green, < 0.1%, yellow < 1%, orange < 10%; and red, > 10%. **Figure S4. **Computational evaluation of different setups for ^13^C metabolic flux analysis of *Synechocystis *6803. The aim of the simulations was to identify optimum strategies for flux analysis in the photomixotrophic microbe. Different setups using different tracer substrates and labelling data were analyzed for the achievable precision and accuracy to determine a flux scenario with low flux (5%) through the ED and the PK pathway. Key fluxes of upper and lower carbon metabolism, i.e., through ED, PP, EMP, and PK pathways, CBB cycle, and TCA cycle, are shown. Each setup was evaluated by a Monte-Carlo approach that mimicked 100 repetitions of the corresponding flux study while taking experimental errors into account. Double, triple, and quadruple tracer studies were evaluated. The substrates shown here, were [1-^13^C], [3-^13^C], [6-^13^C], and [^13^C_6_] glucose for the following reasons. The combination of [1-^13^C] glucose and [6-^13^C] glucose well discriminated the fluxes through the EMP, the PP, and the ED pathway in glucose-grown pseudomonads, revealing a similarly cyclic pathway architecture as cyanobacteria [42]. Metabolization of [3-^13^C] glucose (based on the underlying carbon transitions) via the ED route should selectively lead to ^13^C label enrichment at the C1 of pyruvate (and amino acids derived therefrom), providing a sensitive readout, should this pathway be active. The use of [^13^C_6_] glucose appeared beneficial, likely because it helped to estimate the relative uptake of ^13^C sugar versus (non-labelled) CO2, as previously demonstrated for *Basfia succiniciproducens*, grown on sucrose under high rates of CO_2_ assimilation [22]. The color indicates flux determinability: green, < 0.1%, yellow < 1%, orange < 10%; and red, > 10%. **Figure S5. **Computational evaluation of different setups for ^13^C metabolic flux analysis of *Synechocystis *6803. The aim of the simulations was to identify optimum strategies for flux analysis in the photomixotrophic microbe. Different setups using different tracer substrates and labelling data were analyzed for the achievable precision and accuracy to determine a flux scenario with medium flux (25%) through the ED and the PK pathway. Key fluxes of upper and lower carbon metabolism, i.e., through ED, PP, EMP, and PK pathways, CBB cycle, and TCA cycle, are shown. Each setup was evaluated by a Monte-Carlo approach that mimicked 100 repetitions of the corresponding flux study while taking experimental errors into account. The color indicates flux determinability: green, < 0.1%, yellow < 1%, orange < 10%; and red, > 10%. **Figure S6.** Computational evaluation of different setups for ^13^C metabolic flux analysis of *Synechocystis *6803. The aim of the simulations was to identify optimum strategies for flux analysis in the photomixotrophic microbe. Different setups using different tracer substrates and labelling data were analyzed for the achievable precision and accuracy to determine a flux scenario with high flux (50%) through the ED and the PK pathway. Key fluxes of upper and lower carbon metabolism, i.e., through ED, PP, EMP, and PK pathways, CBB cycle, and TCA cycle, are shown. Each setup was evaluated by a Monte-Carlo approach that mimicked 100 repetitions of the corresponding flux study while taking experimental errors into account. The color indicates flux determinability: green, < 0.1%, yellow < 1%, orange < 10%; and red, > 10%. **Figure S7. **Sensitivity of selected mass isotopomer ratios to a variation of individual flux parameters using alternative single ^13^C labelled glucose as input. The most sensitive change is highlighted. **Figure S8.** Goodness-of-fit for ^13^C metabolic flux analysis of *Synechocystis* 6863. The data reflect measured and model predicted (simulated) data for the best-fit solution: 388 mass isotopomers from amino acids, sugars, and sugar derivatives, measured by GC-MS (**A**) and on basis of 388 mass isotopomers from amino acids, sugars, and sugar derivatives, measured by GC-MS, plus 168 positional ^13^C enrichments, obtained by NMR (**B**). **Figure S9.** In vivo flux distribution of *Synechocystis *6803 during photomixotrophic growth on glucose and CO_2_ determined by GC-MS and NMR based ^13^C metabolic flux analysis. Fluxes are normalized to the glucose uptake (100%, 0.421 mmol g^−1^ h^−1^). The thickness of the arrows denotes the amount of flux. The errors for the fluxes reflect standard deviations, estimated by Monte-Carlo simulation. The anabolic fluxes into biomass are shown as triangles. The complete flux data set is given in **Table S2**, where also the 95% confidence intervals from the Monte-Carlo analysis are provided. *GLC_ex* extracellular glucose; *G6P* glucose 6-phosphate; *F6P* fructose 6-phosphate; *DHAP* dihydroxyacetone phosphate; *GAP* glyceraldehyde 3-phosphate; *3PG* 3-phosphoglycerate; *PEP* phosphoenolpyruvate; *PYR* pyruvate; *AcCoA* acetyl coenzyme A; *ICI* isocitrate; *2OG* 2-oxoglutarate; *SucA* succinate-semialdehyde; *SUC* succinate; *FUM* fumarate; *MAL* malate; *OAA* oxaloacetate; *6PG* 6-phosphogluconate; *KDPG* 2-keto-3-deoxy-6-phosphogluconate; *Ri5P* ribose 5-phosphate; *Ru5P* ribulose 5-phosphate; *X5P* xylose 5-phosphate; *S7P* sedoheptulose 7-phosphate; *E4P* erythrose 4-phosphate; *CO2_EX* extracellular carbon dioxide;*CO*_*2*_ intracellular carbon dioxide. The flux estimation yielded an excellent quality of fit for the considered mass isotopomers of amino acids, sugars, and sugar derivatives and NMR-derived positional enrichments (Additional file 1: Table S3). The variance-weighted sum of squared residuals (SSR) was 583 and thus within the expected range (511; 621) of the chi-square test at 95% confidence level. n = 4. **Figure S10.** Goodness-of-fit for the ^13^C metabolic flux analysis of *Synechocystis* 6863 deletion mutants. The data reflect the fest-fit solution and show measured and simulated GC-MS data (388 mass isotopomers from amino acids, sugars, and sugar derivatives) for strains *Δeda *(**A**), *ΔpfkAB *(**B**), and *Δxfp1/xfp2 *(**C**). **Figure S11. **Evaluation of the light supply during cultivation in glass tubes with 3.5 cm diameter that were illuminated from the front and the back side and were mixed by air, bubbled from the bottom [37]. Simulating the light supply for this geometry, using the obtained Lambert-Beer correlation (Additional file 1: Fig. S1), revealed large inner zones of insufficient illumination, when considering the determined threshold of 35 μE m^−2^ s^−1^. Already at OD750 = 1, cells largely faced limiting light supply, and the light-limited areas became even more pronounced at higher cell concentrations, comprising up to more than 90% of the culture volume. The show modelled light intensity profiles in 200 mL Kniese tubes, illuminated with 50 μE m^−2^ s^−1^ from the back and the front side, during cultivation of *Synechocystis *6830. The calculation was based the measured relationship between cell concentration and light absorption (Fig. 2). The relative light intensities are encoded by different colour and range from green (100%) to black (0%). The colour code shows all areas, illuminated with a light intensity below 35 μE m^−2^ s^−1^ and shown to limit growth, in dark. The values were calculated at a spatial resolution of 0.1 mm. **Figure S12. **Southern blot of wildtype (WT) and Δ*gap2*. The Southern blot was performed in order to verify the completed segregation of Δ*gap2*. The probe detected a fragment in the size of 4916 bp in the wildtype (WT) and of 780 bp in Δ*gap2 *as expected. This result confirmed that Δ*gap2 *was segregated and that no wild type copies were left. In addition, an unspecific fragment of about 4500 bp was detected in Δ*gap2 *as well. **Figure S13. **Southern blots of wildtype (WT), *Δxfp1 *and *Δxfp1/Δxfp2*. Southern blots were performed with probes against *xfp1 *and *xfp2 *in order to check segregation of *Δxfp1*, *Δxfp2*, and *Δxfp1/Δxfp2*. The probe against *xfp1 *was expected to detect a fragment size of 1420 bp in the wildtype and of 2013 bp in *Δxfp1 *(top). The probe against *xfp2 *was expected to detect a fragment in the size of 970 bp in the wildtype and of 731 bp in *Δxfp2 *(bottom)*. *Lanes 4 and 5 in the bottom gel, right from the three strains, are not relevant. The southern blots thus confirmed that *Δxfp1, Δxfp2*, and *Δxfp1/Δxfp2 *were segregated and that no wildtype copies were left. **Table S1.** Measured and simulated GC-MS ^13^C labelling data for ^13^C metabolic flux analysis of photomixotrophic *Synechocystis *6803. The approach involved four parallel isotope studies on different ^13^C glucose tracers. The data represent the best-fit solution after minimizing the variance-weighted sum of square residuals and display the experimentally measured (exp) and model simulated (sim) mass isotopomer distributions of amino acids, sugars, and sugar derivatives. The specified fragments represent the ion clusters considered for the analysis, whereby the number denotes the corresponding monoisotopic mass. The flux fit was statistically acceptable. The variance-weighted sum of square residuals (SSR) was 377 and thus within the expected range (342; 434) of the chi-square test at 95% confidence level. **Table S2. **Flux distributions in *Synechocystis *6863 and related deletion mutants. The data represent the best-fit-solution for each strain and include the estimated fluxes (Mean), the standard deviation (SD) and the corresponding 95% confidence intervals (*LB* lower boundary; *UB* upper boundary). The ^13^C labelling data, considered for flux estimation, were taken from GC-MS and from GC-MS plus NMR analysis. The reaction numbers refer to the biochemical network model (Additional file 1: Table S9). **Table S3.** Measured and simulated GC-MS and NMR ^13^C labelling data for ^13^C metabolic flux analysis of photomixotrophic *Synechocystis *6803. The approach involved four parallel isotope studies on different ^13^C glucose tracers. The data represent the best-fit solution after minimizing the variance-weighted sum of square residuals and display the experimentally measured (exp) and model simulated (sim) GC-MS mass isotopomer distributions of amino acids, sugars, and sugar derivatives plus positional enrichments from NMR analysis. Regarding GC-MS analysis, the specified fragments represent the ion clusters considered for the analysis, whereby the number denotes the corresponding monoisotopic mass. Data has been corrected for natural occurring isotopes. For NMR, the assessed carbon atom is given. The flux fit was statistically acceptable. The variance-weighted sum of squared residuals (SSR) was 583 and thus within the expected range (511; 621) of the chi-square test at 95% confidence level. **Table S4.** Measured and simulated GC-MS ^13^C labelling data for ^13^C metabolic flux analysis of photomixotrophic *Synechocystis *6803 *Δeda*. The approach involved four parallel isotope studies on different ^13^C glucose tracers. The data represent the best-fit solution after minimizing the variance-weighted sum of square residuals and display the experimentally measured (exp) and model simulated (sim) mass isotopomer distributions of amino acids, sugars, and sugar derivatives. The specified fragments represent the ion clusters considered for the analysis, whereby the number denotes the corresponding monoisotopic mass. The flux fit of this mutant was statistically acceptable. The variance-weighted sum of squared residuals (SSR) was 394 and thus within the expected range (343; 435) of the chi-square test at 95% confidence level. **Table S5.** Measured and simulated GC-MS ^13^C labelling data for ^13^C metabolic flux analysis of photomixotrophic *Synechocystis *6803 *ΔpfkA/ΔpfkB*. The approach involved four parallel isotope studies on different ^13^C glucose tracers. The data represent the best-fit solution after minimizing the variance-weighted sum of square residuals and display the experimentally measured (exp) and model simulated (sim) mass isotopomer distributions of amino acids, sugars, and sugar derivatives. The specified fragments represent the ion clusters considered for the analysis, whereby the number denotes the corresponding monoisotopic mass. The flux fit of this mutant was statistically acceptable. The variance-weighted sum of squared residuals (SSR) was 401 and thus within the expected range (343; 435) of the chi-square test at 95% confidence level. **Table S6.** Measured and simulated GC-MS ^13^C labelling data for ^13^C metabolic flux analysis of photomixotrophic *Synechocystis *6803 *Δxfp1/*Δ*xfp2*. The approach involved four parallel isotope studies on different ^13^C glucose tracers. The data represent the best-fit solution after minimizing the variance-weighted sum of square residuals and display the experimentally measured (exp) and model simulated (sim) mass isotopomer distributions of amino acids, sugars, and sugar derivatives. The specified fragments represent the ion clusters considered for the analysis, whereby the number denotes the corresponding monoisotopic mass. The flux fit of this mutant was statistically acceptable. The variance-weighted sum of squared residuals (SSR) was 415 and thus within the expected range (344; 436) of the chi-square test at 95% confidence level. **Table S7. **Primers used to construct a phosphoketolase double deletion mutant *Δxfp1/Dxfp2 *and a single gene deletion mutant *Δgap2 *from wild type. In addition, the corresponding annealing temperature (AT) is given. **Table S8.** Cellular composition used for metabolic flux analysis of *Synechocystis *6863. The data for wild type (WT) were also used for the strains *ΔpfkAB and Δxfp1/Δxfp2*. For strain Δ*eda*, the data reflect the increased glycogen content. **Table S9. **Biochemical reaction network for ^13^C metabolic flux analysis of *Synechocystis *6803 including reaction stoichiometry, atom transition, and reaction directionality. *F* unidirectional (forward only) reaction; *FR* reversible reaction; *B* biomass. The reactions R1 (v_1_) to R34 (v_34_) refer to the carbon core network of the microbe (Fig. 1). The reactions R35 to R84 represent biomass forming reactions. In Fig. 1 they lumped into the corresponding anabolic fluxes (vx).

## Data Availability

The dataset(s) supporting the conclusions of this article are all included within the article.
