# Supplementary material for: GC/MS-based 13C metabolic flux analysis resolves the parallel and cyclic photomixotrophic metabolism of Synechocystis sp. PCC 6803 and selected deletion mutants including the Entner-Doudoroff and phosphoketolase pathways
Source: Microb Cell Fact. 2022 Apr 22;21:69. doi: 10.1186/s12934-022-01790-9 (PMC9034593; doi:10.1186/s12934-022-01790-9)
Supplement: Supplementary file 1 — Additional file 1: Figure S1. Development of a suitable workflow for photomixotrophic growth of Synechocystis 6803 in 13C metabolic flux analysis. Cultivation profile with indicated threshold for growth with sufficient light supply, A estimation of the maximal cell concentration that provides sufficient light for maximum growth, B light absorption of cultures, incubated in BG11 medium, at varied cell concentration and depth, the values are normalized to 100% for the maximum illuminance, C modelling of the relative light intensity as function of cell concentration and light passage (depth) using the Lambert–Beer law, D the orange lines indicate that a culture at OD = 2 and the light path length for the conducted shake flask cultures (42 mm) receives 70% of the supplied illumination, while absorbing the remaining 30%. n = 3. Figure S2. Time profile of 13C amino acid labelling patterns (given at different cell concentrations) during photomixotrophic cultivation of Synechocystis 6803, grown on [1-13C] glucose (left) and [U-13C] glucose (right). Figure S3. Computational evaluation of previous approaches, based on single isotope experiments and (mainly) GC/MS analysis of proteinogenic amino acids, for 13C metabolic flux analysis of photomixotrophic Synechocystis 6803. The tested approaches had been applied for photomixotrophic flux analysis of Synechocystis, before the full network was known [44] or had focused on a subnetwork of the microbe [45]. A third approach had been derived to analyze heterotrophic Synechocystis sp. PCC6803 without an active CBB cycle [55]. There, the approaches analyzed for the achievable precision and accuracy to determine fluxes in a scenario with 0% (left), 5% (middle), and 50% (right) flux through the ED and the PK pathways. The show the outcome of a Monte-Carlo simulation that mimicked 100 repetitions of the corresponding flux study while taking experimental errors into account. Displayed are key fluxes of upper and lower carbon metabolism, i [file 12934_2022_1790_MOESM1_ESM.pdf]

Supplement to:

GC/MS-based ^{13}C metabolic flux analysis resolves the parallel and cyclic photomixotrophic metabolism of *Synechocystis* sp. PCC 6803 and selected deletion mutants including the Entner-Doudoroff and phosphoketolase pathways

Microbial Cell Factories

Dennis Schulze[1], Michael Kohlstedt[1], Judith Becker[1], Edern Cahoreau[2,3,4], Lindsay Peyriga[2,3,4], Alexander Makowka[5], Sarah Hildebrandt[5], Kirstin Gutekunst[5,6], Jean-Charles Portais[2,3,4], and Christoph Wittmann[1*]

[1] Institute of Systems Biotechnology, Saarland University, Saarbrücken, Germany

[2] Toulouse Biotechnology Institute, Université de Toulouse, CNRS, INRAE, INSA, Toulouse, France

[3] MetaboHUB-MetaToul, National Infrastructure of Metabolomics & Fluxomics, Toulouse, France

[4] RESTORE, Université de Toulouse, Inserm U1031, CNRS 5070, UPS, EFS, Toulouse, France

[5] Institute of Botany, Christian-Albrecht University of Kiel, Germany

[6] Molecular Plant Physiology, Bioenergetics in Photoautotrophs, University Kassel, Germany

* Phone/FAX: 0049-681-302-71970/71972, Email: christoph.wittmann@uni-saarland.de

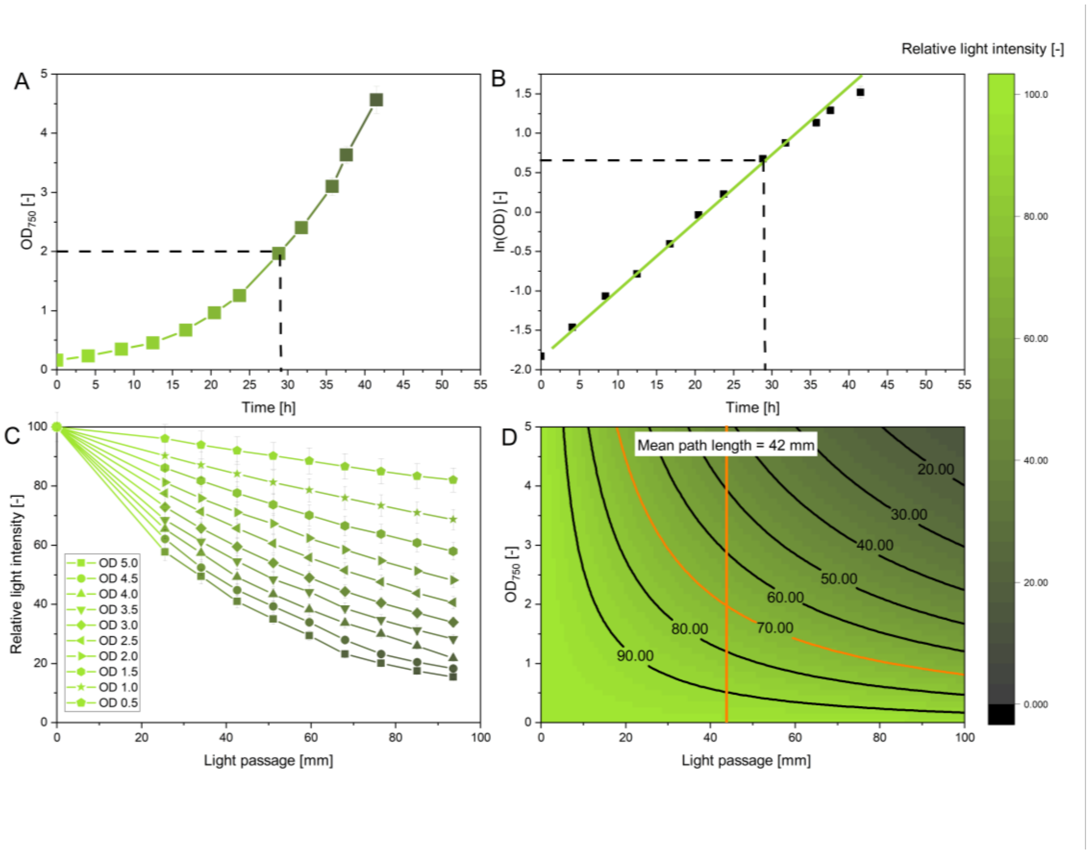

Figure S1: Development of a suitable workflow for photomixotrophic growth of *Synechocystis* 6803 in ^{13}C metabolic flux analysis. Cultivation profile with indicated threshold for growth with sufficient light supply, A. Estimation of the maximal cell concentration that provides sufficient light for maximum growth, B. Light absorption of cultures, incubated in BG11 medium, at varied cell concentration and depth, the values are normalized to 100 % for the maximum illuminance, C. Modelling of the relative light intensity as function of cell concentration and light passage (depth) using the Lambert-Beer law, D. The orange lines indicate that a culture at OD = 2 and the light path length for the conducted shake flask cultures (42 mm) receives 70 % of the supplied illumination, while absorbing the remaining 30%. n=3.

The development was done es follows. Careful inspection of photomixotrophic cultures of *Synechocystis* sp. PCC 6803 revealed that cells grew exponentially only up to a cell density of OD_{750} = 2 (AB). Tested over a broad range, cell suspensions of different concentration (OD_{750} from 0.5 to 5.0) and thickness (1 to 10 mm) absorbed light according to the Lambert-Beer law (C). The obtained linear relationship could be used to infer the light intensity in growing cultures, considering that illumination was applied from above, resulting in a mean path length of 42 mm for the light through the culture

(D). In consequence, cells at OD_{750} = 2 absorbed 30% of the imposed light, resulting in a remaining light intensity of 35 µS m^{-2} s^{-1}. Given the growth behavior, OD_{750} = 2 was considered as upper boundary that should not be exceeded to avoid growth limitations and metabolic changes by reduced light supply. Consequently, all ^{13}C tracer cultures were harvested for ^{13}C labelling analysis between OD_{750} 1.0 – 1.5. Subsequently, another round of experiments aimed to minimize the impact of non-labelled cells from the inoculum on the later ^{13}C labelling analysis. Inoculation of ^{13}C tracer cultures with non-labelled cells at very low initial cell concentration (OD_{750} < 0.01), below 1% of the cell concentration planned to be harvested, as commonly done for other microbes [22, 52, 53], did not provide satisfying results. Because of the small inoculum, the cultures exhibited lag-phases of up to several days, and parallel cultures did not achieve sufficient reproducibility (data not shown). This problem was overcome by growing cells on the corresponding ^{13}C tracer in two steps: a ^{13}C pre-culture (from $OD_{750\,=}$ 0.1 to about 1.5), inoculated into a subsequent ^{13}C main culture (again grown from OD_{750} = 0.1 to about 1.5), finally excluding inoculum effects on labelling analysis. Regarding the experimental setup, it should be noted that the use of self-constructed translucent caps (see materials and methods) supported illumination: viewed from above, a common aluminum cap otherwise covered approximately 50% of the shake flask area, substantially reducing light supply from the upper illumination panel of the shaker.

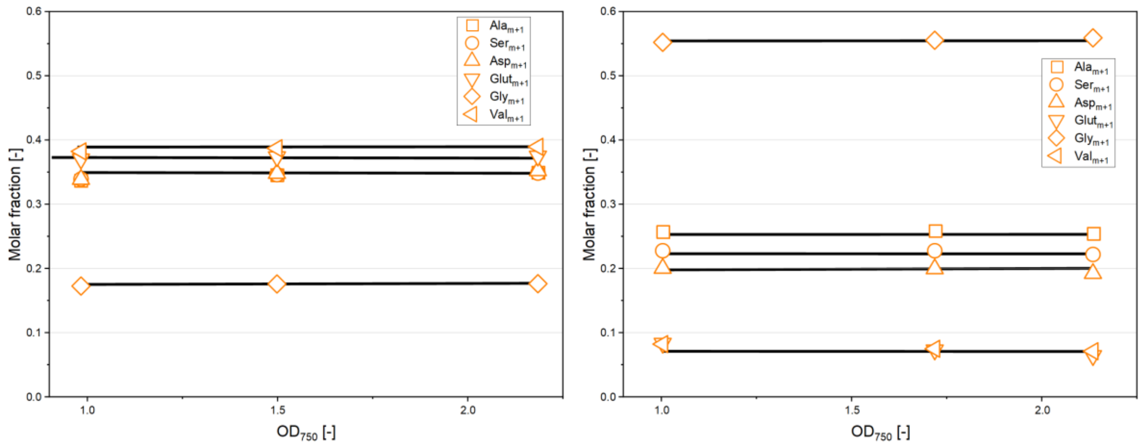

Figure S2: Time profile of ^{13}C amino acid labelling patterns (given at different cell concentrations) during photomixotrophic cultivation of *Synechocystis* 6803, grown on [1-^{13}C] glucose (left) and [U-^{13}C] glucose (right).

	Previous studies		
^{13}C- Glucose	[1,2] Glucose	[1]/[U] (70/30) Glucose	[1] Glucose
Experiment		Single	
GC-MS AA	●	●	●
GC-MS Sugar			
NMR AA			

	Previous studies		
^{13}C- Glucose	[1,2] Glucose	[1]/[U] (70/30) Glucose	[1] Glucose
Experiment		Single	
GC-MS AA	●	●	●
GC-MS Sugar			
NMR AA			

	Previous studies		
^{13}C- Glucose	[1,2] Glucose	[1]/[U] (70/30) Glucose	[1] Glucose
Experiment		Single	
GC-MS AA	●	●	●
GC-MS Sugar			
NMR AA			

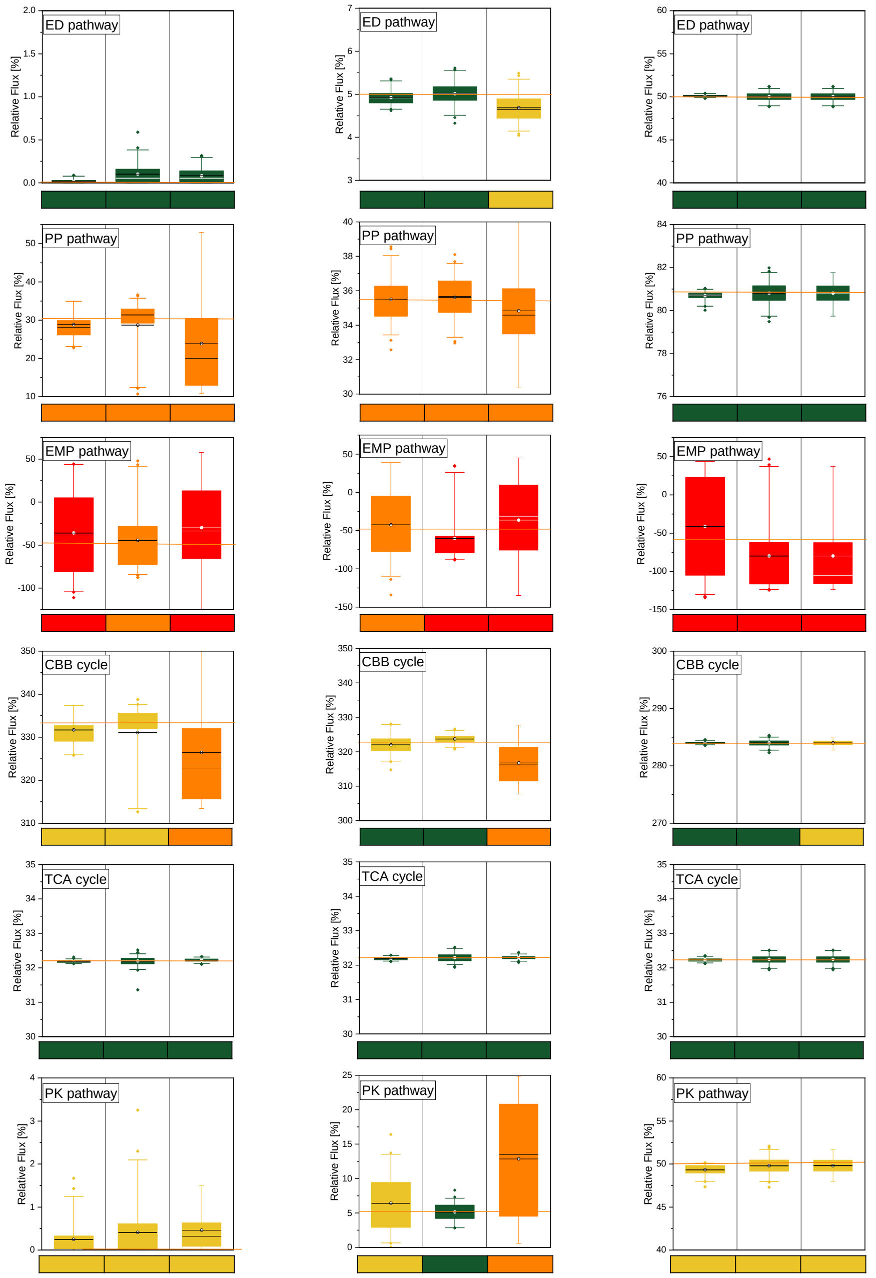

Figure S3. Computational evaluation of previous approaches, based on single isotope experiments and (mainly) GC/MS analysis of proteinogenic amino acids, for ^{13}C metabolic flux analysis of photomixotrophic *Synechocystis* 6803. The tested approaches had been applied for photomixotrophic flux analysis of *Synechocystis*, before the full network was known [44] or had focused on a subnetwork of the microbe [45]. A third approach had been derived to analyze heterotrophic *Synechocystis* sp.

PCC6803 without an active CBB cycle [55]. There, the approaches analyzed for the achievable precision and accuracy to determine fluxes in a scenario with 0% (left), 5% (middle), and 50% (right) flux through the ED and the PK pathways. The show the outcome of a Monte-Carlo simulation that mimicked 100 repetitions of the corresponding flux study while taking experimental errors into account. Displayed are key fluxes of upper and lower carbon metabolism, i. e. through ED, PP, EMP, and PK pathways, CBB cycle, and TCA cycle, are shown. The color indicates the determinability of a flux parameter. The color indicates flux determinability: green, < 0.1%, yellow < 1%, orange < 10%; and red, > 10%.

	Novel combinations of ^{13}C tracer substrates with varying analytes and methods																							
^{13}C- Glucose	[1] + [U] Glucose				[1] + [3] + [6] Glucose				[1] + [3]+ [U] Glucose					[1] + [6] + [U] Glucose			[3] + [6] + [U] Glucose				[1] + [3] +[6] +[U] Glucose			
Experiment	Double				Triple																Quadruple			
GC-MS AA	●	●	●	●	●	●	●	●	●	●	●	●	●	●	●	●	●	●	●	●	●	●	●	●
GC-MS Sugar		●		●		●		●		●		●		●		●		●		●		●		●
NMR AA			●	●			●	●			●	●			●	●			●	●			●	●

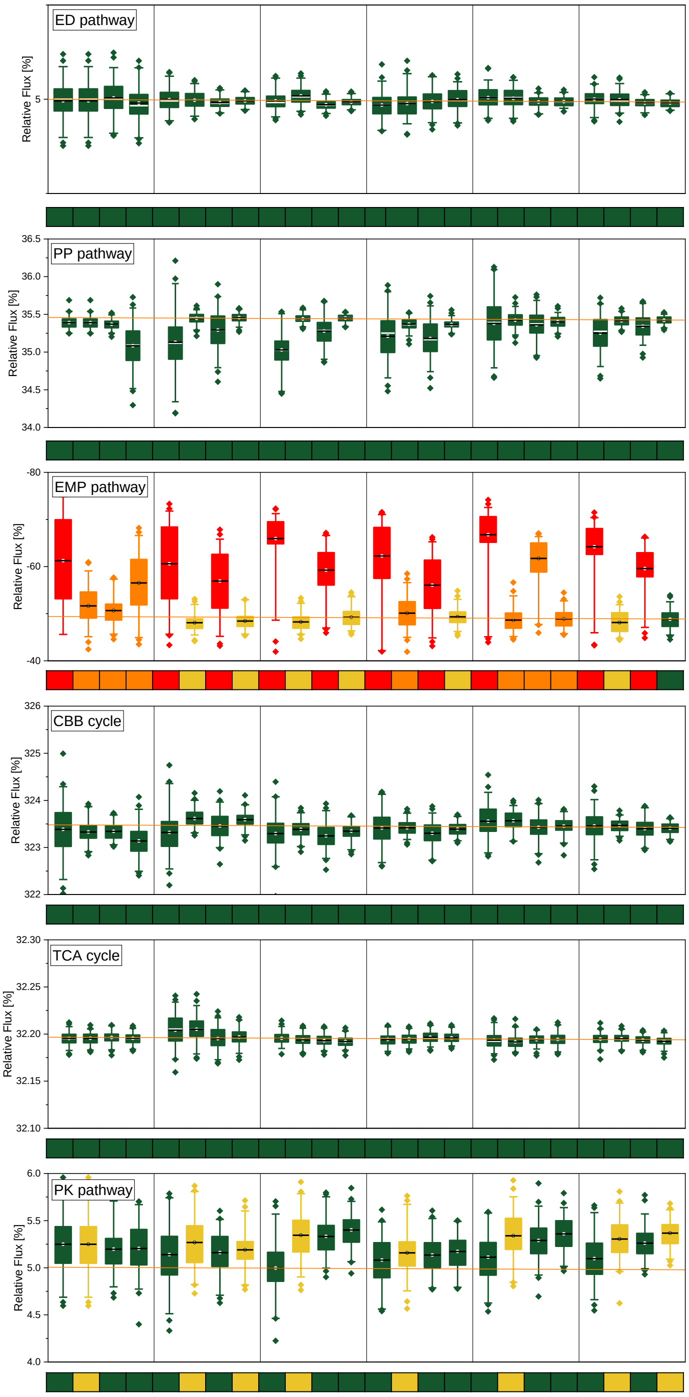

Figure S4. Computational evaluation of different setups for ^{13}C metabolic flux analysis of *Synechocystis* 6803. The aim of the simulations was to identify optimum strategies for flux analysis in the photomixotrophic microbe. Different setups using

different tracer substrates and labelling data were analyzed for the achievable precision and accuracy to determine a flux scenario with low flux (5%) through the ED and the PK pathway. Key fluxes of upper and lower carbon metabolism, i. e. through ED, PP, EMP, and PK pathways, CBB cycle, and TCA cycle, are shown. Each setup was evaluated by a Monte-Carlo approach that mimicked 100 repetitions of the corresponding flux study while taking experimental errors into account. Double, triple, and quadruple tracer studies were evaluated. The substrates shown here, were [1-^{13}C], [3-^{13}C], [6-^{13}C], and [$^{13}C_6$] glucose for the following reasons. The combination of [1-^{13}C] glucose and [6-^{13}C] glucose well discriminated the fluxes through the EMP, the PP, and the ED pathway in glucose-grown pseudomonads, revealing a similarly cyclic pathway architecture as cyanobacteria [42]. Metabolization of [3-^{13}C] glucose (based on the underlying carbon transitions) via the ED route should selectively lead to ^{13}C label enrichment at the C_1 of pyruvate (and amino acids derived therefrom), providing a sensitive readout, should this pathway be active. The use of [$^{13}C_6$] glucose appeared beneficial, likely because it helped to estimate the relative uptake of ^{13}C sugar versus (non-labelled) CO_2, as previously demonstrated for *Basfia succiniciproducens*, grown on sucrose under high rates of CO_2 assimilation [22]. The color indicates flux determinability: green, < 0.1%, yellow < 1%, orange < 10%; and red, > 10%.

	Novel combinations of ^{13}C tracer substrates with varying analytes and methods																							
^{13}C- Glucose	[1] + [U] Glucose				[1] + [3] + [6] Glucose				[1] + [3]+ [U] Glucose				[1] + [6] + [U] Glucose				[3] + [6] + [U] Glucose				[1] + [3] +[6] +[U] Glucose			
Experiment	Double				Triple																Quadruple			
GC-MS AA	●	●	●	●	●	●	●	●	●	●	●	●	●	●	●	●	●	●	●	●	●	●	●	●
GC-MS Sugar		●		●		●		●		●		●		●		●		●		●		●		●
NMR AA			●	●			●	●			●	●			●	●			●	●			●	●

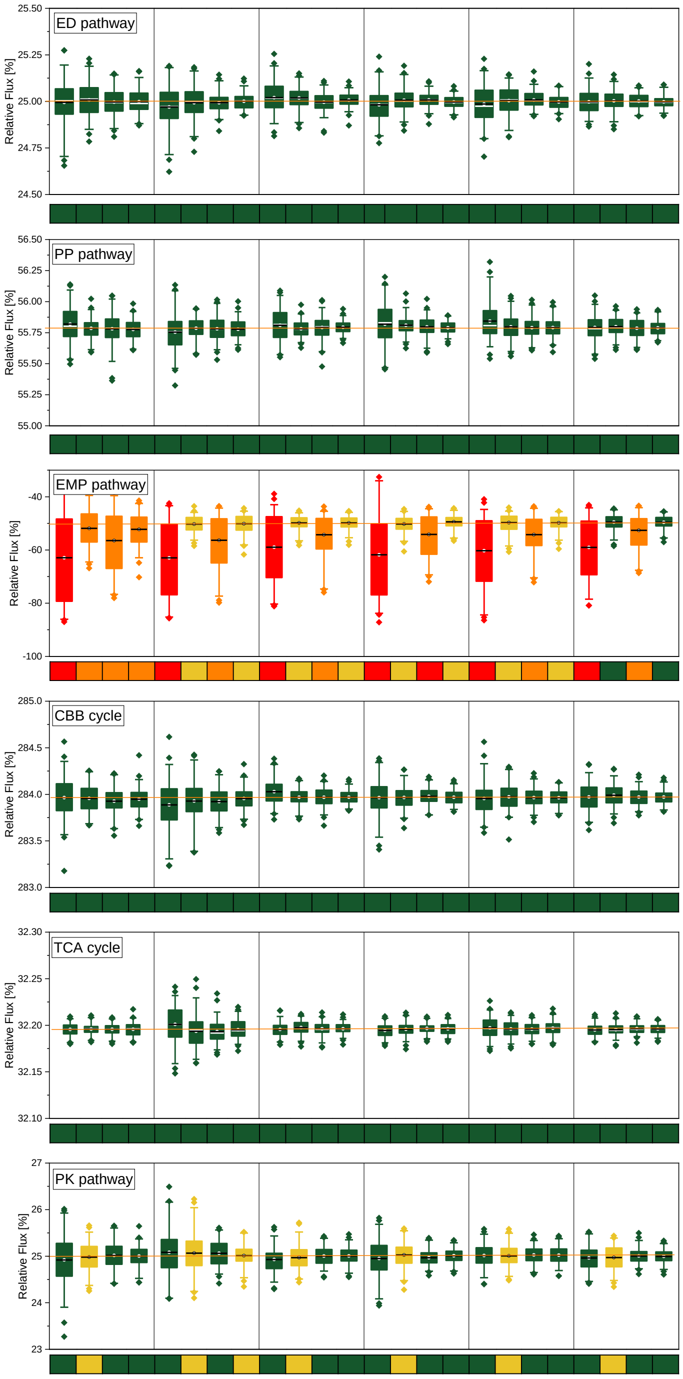

Figure S5. Computational evaluation of different setups for ^{13}C metabolic flux analysis of *Synechocystis* 6803. The aim of the simulations was to identify optimum strategies for flux analysis in the photomixotrophic microbe. Different setups using different tracer substrates and labelling data were analyzed for the achievable

precision and accuracy to determine a flux scenario with medium flux (25%) through the ED and the PK pathway. Key fluxes of upper and lower carbon metabolism, i. e. through ED, PP, EMP, and PK pathways, CBB cycle, and TCA cycle, are shown. Each setup was evaluated by a Monte-Carlo approach that mimicked 100 repetitions of the corresponding flux study while taking experimental errors into account. The color indicates flux determinability: green, < 0.1%, yellow < 1%, orange < 10%; and red, > 10%.

	Novel combinations of ^{13}C tracer substrates with varying analytes and methods																							
^{13}C- Glucose	[1] + [U] Glucose				[1] + [3] + [6] Glucose				[1] + [3]+ [U] Glucose				[1] + [6] + [U] Glucose				[3] + [6] + [U] Glucose				[1] + [3] +[6] +[U] Glucose			
Experiment	Double				Triple																Quadruple			
GC-MS AA	●	●	●	●	●	●	●	●	●	●	●	●	●	●	●	●	●	●	●	●	●	●	●	●
GC-MS Sugar		●		●		●		●		●		●		●		●		●		●		●		●
NMR AA			●	●			●	●			●	●			●	●			●	●			●	●

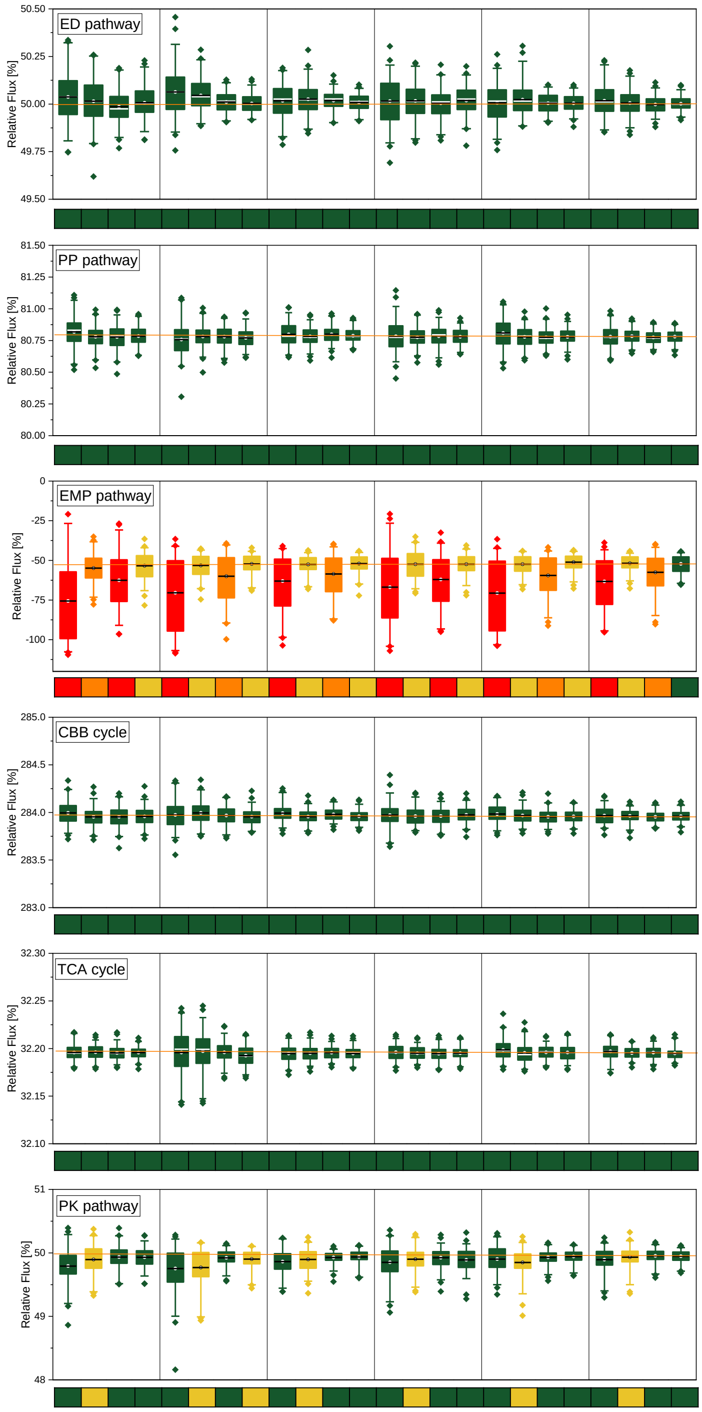

Figure S6: Computational evaluation of different setups for ^{13}C metabolic flux analysis of *Synechocystis* 6803. The aim of the simulations was to identify optimum strategies for flux analysis in the photomixotrophic microbe. Different setups using

different tracer substrates and labelling data were analyzed for the achievable precision and accuracy to determine a flux scenario with high flux (50%) through the ED and the PK pathway. Key fluxes of upper and lower carbon metabolism, i. e. through ED, PP, EMP, and PK pathways, CBB cycle, and TCA cycle, are shown. Each setup was evaluated by a Monte-Carlo approach that mimicked 100 repetitions of the corresponding flux study while taking experimental errors into account. The color indicates flux determinability: green, < 0.1%, yellow < 1%, orange < 10%; and red, > 10%.

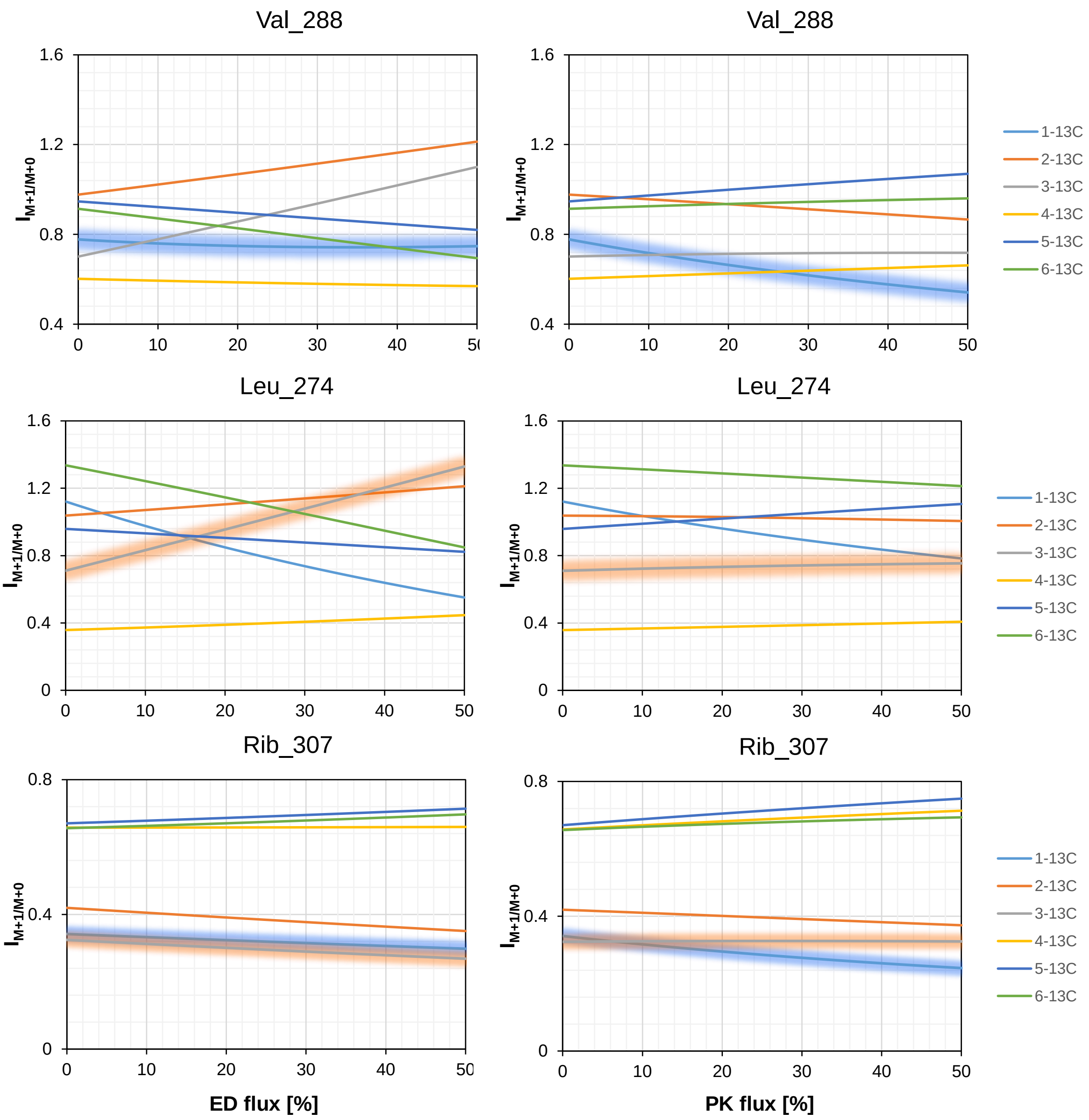

Figure S7. Sensitivity of selected mass isotopomer ratios to a variation of individual flux parameters using alternative single ^{13}C labelled glucose as input. The most sensitive change is highlighted.

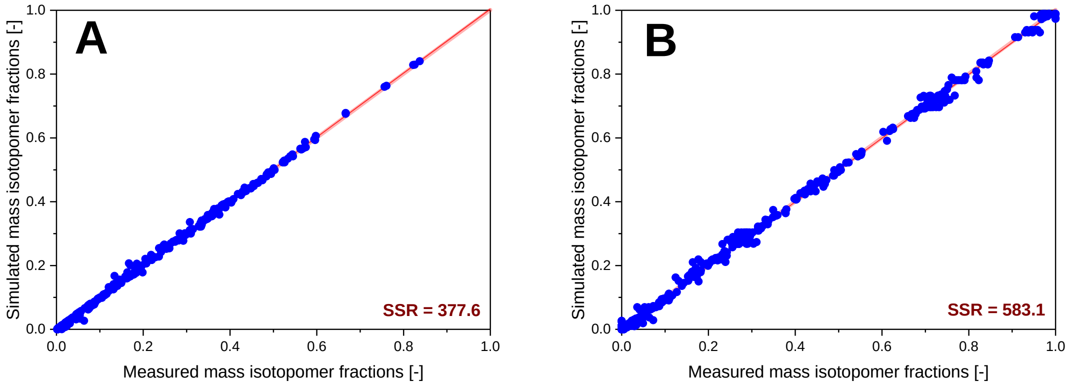

Figure S8: Goodness-of-fit for ^{13}C metabolic flux analysis of Synechocystis 6863. The data reflect measured and model predicted (simulated) data for the best-fit solution: 388 mass isotopomers from amino acids, sugars, and sugar derivatives, measured by GC-MS (A) and on basis of 388 mass isotopomers from amino acids, sugars, and sugar derivatives, measured by GC-MS, plus 168 positional ^{13}C enrichments, obtained by NMR (B).

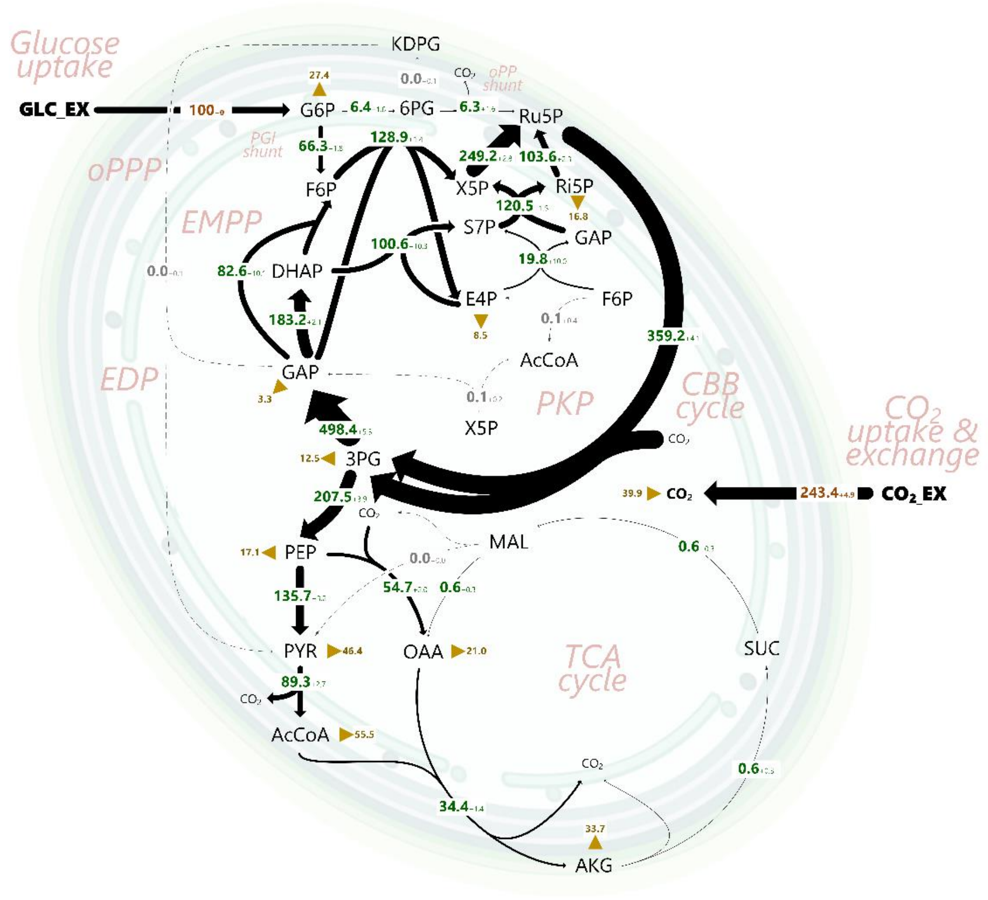

Fig. S9. In vivo flux distribution of *Synechocystis* 6803 during photomixotrophic growth on glucose and CO_2 determined by GC-MS and NMR based ^{13}C metabolic flux analysis. Fluxes are normalized to the glucose uptake (100 %, 0.421 mmol g^{-1} h^{-1}). The thickness of the arrows denotes the amount of flux. The errors for the fluxes reflect standard deviations, estimated by Monte-Carlo simulation. The anabolic fluxes into biomass are shown as triangles. The complete flux data set is given in **Table S2**, where also the 95% confidence intervals from the Monte-Carlo analysis are provided. Abbreviations: extracellular glucose (GLC_ex), glucose 6-phosphate (G6P), fructose 6-phosphate (F6P), dihydroxyacetone phosphate (DHAP), glyceraldehyde 3-phosphate (GAP), 3-phosphoglycerate (3PG), phosphoenolpyruvate (PEP), pyruvate (PYR), acetyl coenzyme A (AcCOA), isocitrate (ICI), 2-oxoglutarate (2OG), succinate-

semialdehyde (SucA), succinate (SUC), fumarate (FUM), malate (MAL), oxaloacetate (OAA), 6-phosphogluconate (6PG), 2-keto-3-deoxy-6-phosphogluconate (KDPG), ribose 5-phosphate (Ri5P), ribulose 5-phosphate (Ru5P), xylose 5-phosphate (X5P), sedoheptulose 7-phosphate (S7P), erythrose 4-phosphate (E4P), extracellular carbon dioxide (CO2_EX), intracellular carbon dioxide (CO2). The flux estimation yielded an excellent quality of fit for the considered mass isotopomers of amino acids, sugars, and sugar derivatives and NMR-derived positional enrichments (**Table S3**). The variance-weighted sum of squared residuals (SSR) was 583 and thus within the expected range (511; 621) of the chi-square test at 95% confidence level. n=4.

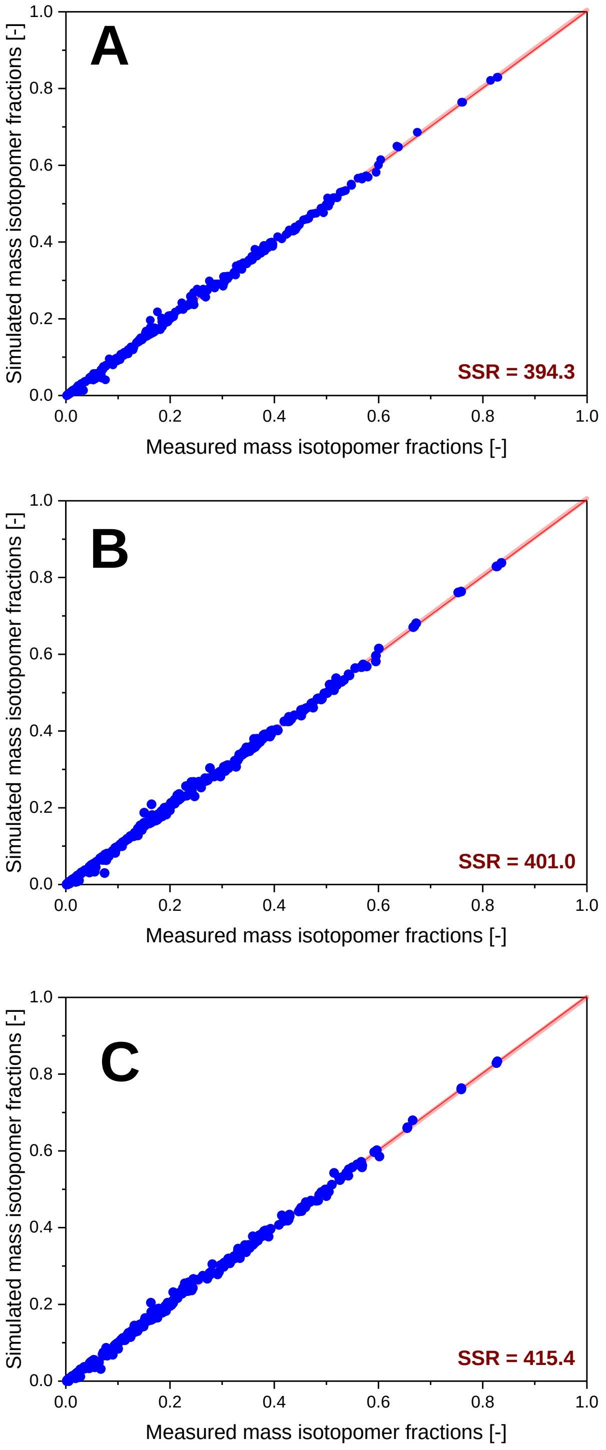

Figure S10: Goodness-of-fit for the ^{13}C metabolic flux analysis of Synechocystis 6863 deletion mutants. The data reflect the fest-fit solution and show measured and simulated GC-MS data (388 mass isotopomers from amino acids, sugars, and sugar derivatives) for strains *Δeda* (A), *ΔpfkAB* (B), and *Δxfp1/xfp2* (C).

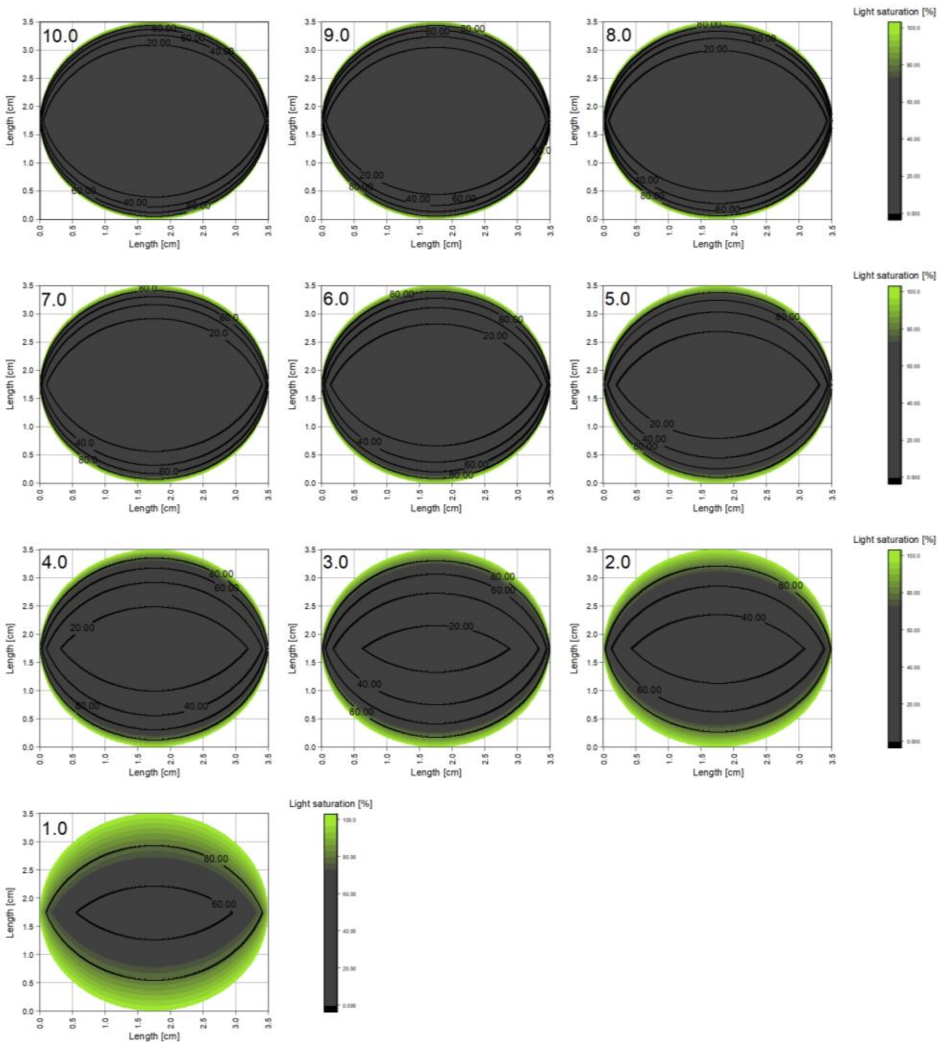

Figure S11: Evaluation of the light supply during cultivation in glass tubes with 3.5 cm diameter that were illuminated from the front and the back side and were mixed by air, bubbled from the bottom [37]. Simulating the light supply for this geometry, using the obtained Lambert-Beer correlation (**Fig. S1**), revealed large inner zones of insufficient illumination, when considering the determined threshold of 35 µE m^{-2} s^{-1}. Already at OD_{750} = 1, cells largely faced limiting light supply, and the light-limited areas became even more pronounced at higher cell concentrations, comprising up to more than 90% of the culture volume. The show modelled light intensity profiles in 200 mL Kniese tubes, illuminated with 50 µE m^{-2} s^{-1} from the back and the front side, during cultivation of *Synechocystis* 6830. The calculation was based the measured relationship between cell concentration and light absorption (**Fig. 2**). The relative light intensities are encoded by different colour and range from green (100 %) to black (0 %). The colour code shows all areas, illuminated with a light intensity below 35 µE m^{-2} s^{-1} and shown to limit growth, in dark. The values were calculated at a spatial resolution of 0.1 mm.

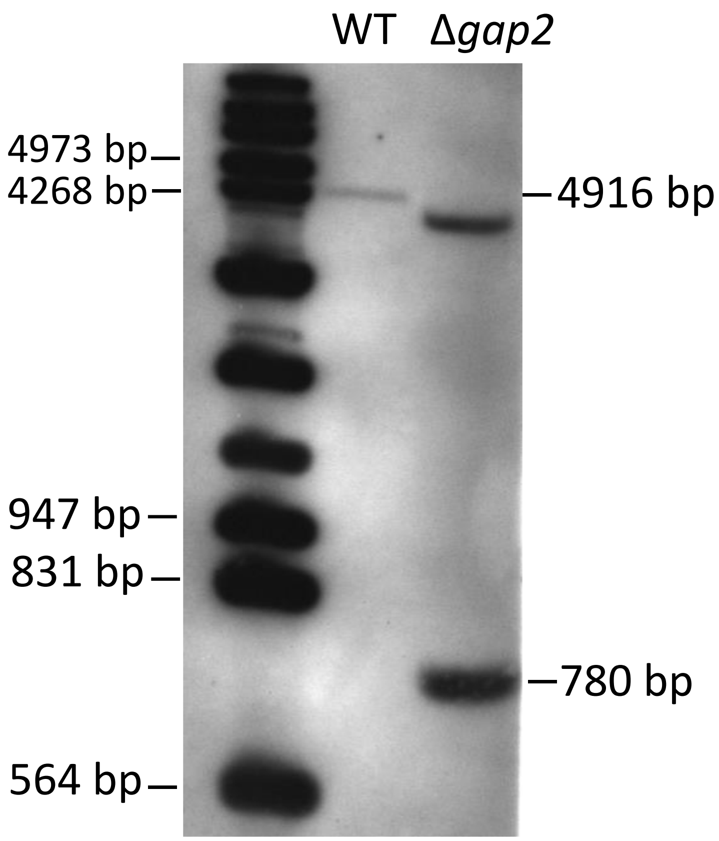

Figure S12. Southern blot of wildtype (WT) and Δ*gap2*. The Southern blot was performed in order to verify the completed segregation of Δ*gap2*. The probe detected a fragment in the size of 4,916 bp in the wildtype (WT) and of 780 bp in Δ*gap2* as expected. This result confirmed that Δ*gap2* was segregated and that no wild type copies were left. In addition, an unspecific fragment of about 4,500 bp was detected in Δ*gap2* as well.

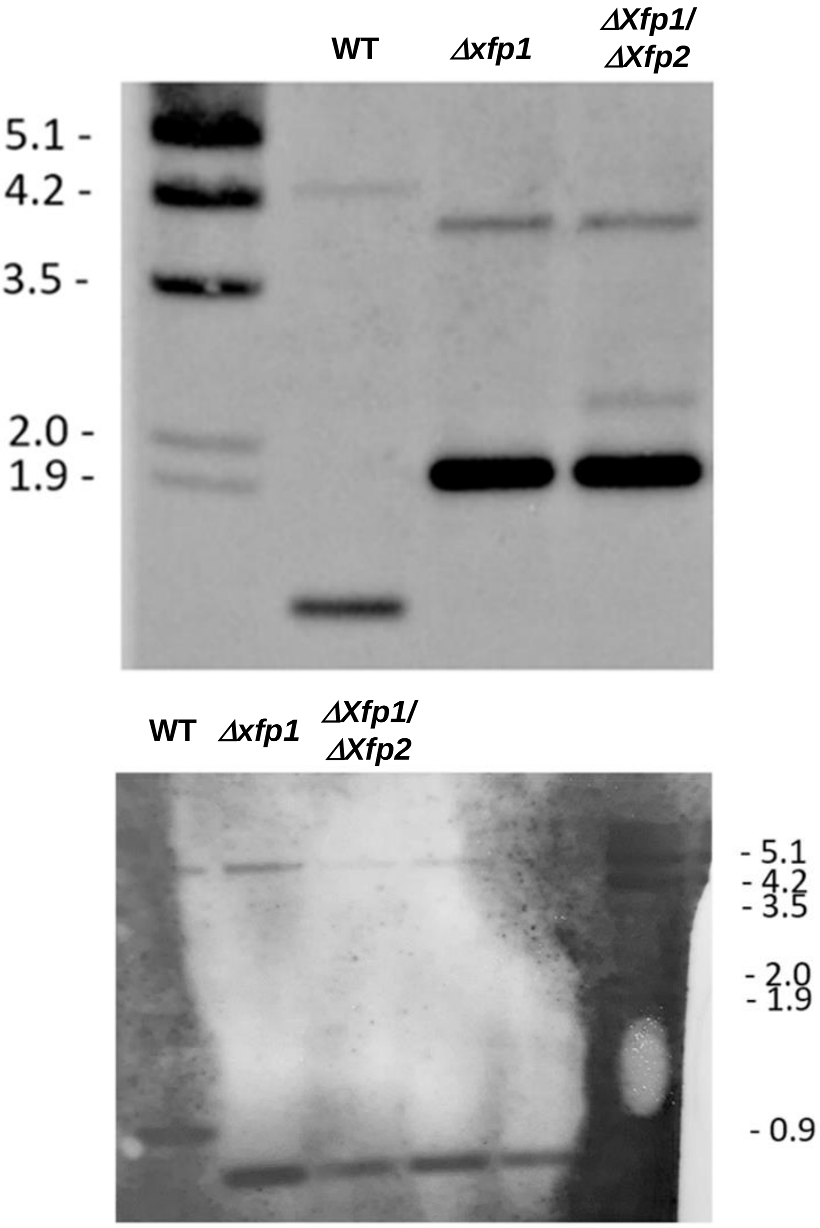

Figure S13. Southern blots of wildtype (WT), Δ*xfp1* and Δ*xfp1*/Δ*xfp2*. Southern blots were performed with probes against *xfp1* and *xfp2* in order to check segregation of Δ*xfp1*, Δ*xfp2*, and Δ*xfp1*/Δ*xfp2*. The probe against *xfp1* was expected to detect a fragment size of 1420 bp in the wildtype and of 2013 bp in Δ*xfp1* (top). The probe against *xfp2* was expected to detect a fragment in the size of 970 bp in the wildtype and of 731 bp in Δ*xfp2* (bottom). Lanes 4 and 5 in the bottom gel, right from the three strains, are not relevant. The southern blots thus confirmed that Δ*xfp1,* Δ*xfp2*, and Δ*xfp1*/Δ*xfp2* were segregated and that no wildtype copies were left.

Table S1: Measured and simulated GC-MS ^{13}C labelling data for ^{13}C metabolic flux analysis of photomixotrophic *Synechocystis* 6803. The approach involved four parallel isotope studies on different ^{13}C glucose tracers. The data represent the best-fit solution after minimizing the variance-weighted sum of square residuals and display the experimentally measured (exp) and model simulated (sim) mass isotopomer distributions of amino acids, sugars, and sugar derivatives. The specified fragments represent the ion clusters considered for the analysis, whereby the number denotes the corresponding monoisotopic mass. The flux fit was statistically acceptable. The variance-weighted sum of square residuals (SSR) was 377 and thus within the expected range (342; 434) of the chi-square test at 95% confidence level.

Fragment	[1-^{13}C] glucose		[3-^{13}C] glucose		[6 -^{13}C] glucose		[$^{13}C_6$] glucose	
	exp	sim	exp	sim	exp	sim	exp	sim
ala_260	0.5222	0.5246	0.5688	0.5671	0.5452	0.5422	0.0894	0.0841
	0.3489	0.3470	0.2934	0.2948	0.3322	0.3342	0.1922	0.1977
	0.1003	0.1000	0.1099	0.1104	0.0960	0.0967	0.4549	0.4488
	0.0286	0.0285	0.0279	0.0277	0.0266	0.0269	0.2634	0.2694
ala_232	0.5439	0.5483	0.5941	0.5956	0.5653	0.5642	0.1050	0.0986
	0.3584	0.3543	0.2981	0.2960	0.3399	0.3405	0.2283	0.2258
	0.0978	0.0975	0.1078	0.1084	0.0949	0.0953	0.6667	0.6756
gly_246	0.7561	0.7600	0.5977	0.6065	0.7606	0.7633	0.1319	0.1328
	0.1732	0.1695	0.3091	0.3017	0.1692	0.1667	0.5620	0.5662
	0.0707	0.0704	0.0932	0.0919	0.0702	0.0700	0.3061	0.3010
gly_218	0.8223	0.8286	0.6667	0.6781	0.8258	0.8292	0.1626	0.1595
	0.1777	0.1714	0.3333	0.3219	0.1742	0.1708	0.8374	0.8405
val_288	0.3582	0.3584	0.4255	0.4205	0.3896	0.3819	0.0221	0.0087
	0.3944	0.3989	0.3423	0.3458	0.3850	0.3915	0.0482	0.0386
	0.1798	0.1771	0.1626	0.1640	0.1649	0.1660	0.1363	0.1442
	0.0519	0.0515	0.0523	0.0536	0.0470	0.0479	0.2365	0.2544
	0.0129	0.0121	0.0141	0.0134	0.0112	0.0110	0.3622	0.3599
	0.0027	0.0019	0.0032	0.0026	0.0022	0.0017	0.1946	0.1943
val_260	0.3589	0.3655	0.4289	0.4317	0.3892	0.3881	0.0239	0.0099
	0.3977	0.4011	0.3453	0.3451	0.3877	0.3928	0.0539	0.0435
	0.1798	0.1734	0.1615	0.1600	0.1653	0.1630	0.1633	0.1785
	0.0513	0.0488	0.0510	0.0509	0.0468	0.0458	0.2839	0.3009
	0.0124	0.0112	0.0133	0.0124	0.0110	0.0102	0.4750	0.4673
leu_274	0.2604	0.2538	0.4047	0.4033	0.2925	0.2775	0.0137	0.0026
	0.3829	0.3898	0.3489	0.3498	0.3834	0.3910	0.0309	0.0184
	0.2412	0.2425	0.1703	0.1714	0.2223	0.2281	0.0870	0.0776
	0.0874	0.0867	0.0570	0.0578	0.0775	0.0791	0.1854	0.2056
	0.0230	0.0226	0.0155	0.0148	0.0200	0.0203	0.3074	0.3363

	0.0051	0.0046	0.0035	0.0029	0.0043	0.0040	0.3755	0.3595
ile_274	0.3488	0.3585	0.4182	0.4244	0.3789	0.3825	0.0207	0.0086
	0.3955	0.3998	0.3440	0.3456	0.3862	0.3922	0.0479	0.0380
	0.1841	0.1771	0.1650	0.1623	0.1701	0.1658	0.1434	0.1540
	0.0543	0.0509	0.0540	0.0524	0.0496	0.0473	0.2485	0.2659
	0.0139	0.0118	0.0149	0.0129	0.0123	0.0107	0.4081	0.4083
	0.0034	0.0018	0.0039	0.0024	0.0030	0.0016	0.1314	0.1252
ser_390	0.4567	0.4557	0.4943	0.4927	0.4755	0.4708	0.0805	0.0767
	0.3546	0.3550	0.3149	0.3141	0.3431	0.3456	0.1864	0.1891
	0.1404	0.1409	0.1442	0.1461	0.1358	0.1373	0.4370	0.4332
	0.0482	0.0485	0.0466	0.0471	0.0455	0.0463	0.2961	0.3010
ser_362	0.4846	0.4858	0.5258	0.5274	0.5028	0.4995	0.0969	0.0946
	0.3735	0.3711	0.3282	0.3242	0.3593	0.3602	0.2362	0.2278
	0.1419	0.1431	0.1460	0.1484	0.1380	0.1403	0.6669	0.6776
thr_404	0.4438	0.4425	0.4837	0.4801	0.4633	0.4596	0.0686	0.0656
	0.3531	0.3530	0.3096	0.3127	0.3425	0.3437	0.1587	0.1654
	0.1416	0.1432	0.1454	0.1467	0.1366	0.1387	0.3754	0.3798
	0.0493	0.0496	0.0480	0.0479	0.0463	0.0471	0.2816	0.2785
	0.0122	0.0117	0.0133	0.0126	0.0112	0.0109	0.1157	0.1108
thr_376	0.4537	0.4556	0.4974	0.4969	0.4721	0.4713	0.0785	0.0744
	0.3590	0.3560	0.3123	0.3129	0.3469	0.3461	0.1810	0.1835
	0.1394	0.1404	0.1441	0.1443	0.1354	0.1368	0.5259	0.5233
	0.0480	0.0479	0.0463	0.0459	0.0456	0.0458	0.2147	0.2188
phe_336	0.2930	0.2899	0.1454	0.1559	0.1405	0.1361	0.0122	0.0003
	0.3806	0.3885	0.3713	0.3780	0.3635	0.3774	0.0122	0.0023
	0.2137	0.2191	0.2770	0.2805	0.3073	0.3108	0.0242	0.0108
	0.0748	0.0770	0.1333	0.1294	0.1302	0.1285	0.0449	0.0322
	0.0210	0.0206	0.0462	0.0427	0.0383	0.0372	0.0798	0.0767
	0.0072	0.0042	0.0153	0.0109	0.0109	0.0084	0.1206	0.1323
	0.0027	0.0007	0.0047	0.0022	0.0030	0.0014	0.1702	0.2009
	0.0023	0.0001	0.0024	0.0004	0.0019	0.0002	0.2049	0.2210
	0.0027	0.0000	0.0028	0.0000	0.0027	0.0000	0.2198	0.2171
	0.0019	0.0000	0.0018	0.0000	0.0017	0.0000	0.1113	0.1064
asp_418	0.4483	0.4416	0.4839	0.4791	0.4646	0.4586	0.0756	0.0655
	0.3494	0.3523	0.3096	0.3122	0.3409	0.3430	0.1640	0.1652
	0.1415	0.1439	0.1457	0.1474	0.1372	0.1394	0.3731	0.3794
	0.0490	0.0502	0.0479	0.0484	0.0464	0.0477	0.2743	0.2785
	0.0118	0.0120	0.0129	0.0129	0.0109	0.0112	0.1130	0.1114
asp_390	0.4563	0.4547	0.4965	0.4960	0.4724	0.4704	0.0823	0.0743
	0.3570	0.3555	0.3129	0.3124	0.3466	0.3455	0.1831	0.1834
	0.1392	0.1412	0.1445	0.1451	0.1356	0.1376	0.5216	0.5231
	0.0476	0.0486	0.0461	0.0465	0.0454	0.0464	0.2130	0.2191
glu_432	0.3105	0.3076	0.3667	0.3642	0.3350	0.3282	0.0179	0.0076
	0.3755	0.3794	0.3378	0.3396	0.3709	0.3752	0.0428	0.0347
	0.2072	0.2063	0.1905	0.1907	0.1956	0.1967	0.1314	0.1413
	0.0778	0.0780	0.0751	0.0759	0.0724	0.0735	0.2389	0.2541

	0.0235	0.0233	0.0238	0.0237	0.0212	0.0215	0.4031	0.3971
	0.0054	0.0053	0.0061	0.0060	0.0048	0.0049	0.1660	0.1652
arg_442	0.3098	0.3001	0.3601	0.3568	0.3300	0.3217	0.0199	0.0067
	0.3717	0.3785	0.3346	0.3411	0.3691	0.3753	0.0454	0.0312
	0.2062	0.2105	0.1925	0.1932	0.1970	0.2001	0.1286	0.1273
	0.0782	0.0803	0.0767	0.0774	0.0731	0.0751	0.2181	0.2335
	0.0242	0.0241	0.0255	0.0242	0.0221	0.0220	0.3603	0.3666
	0.0071	0.0055	0.0078	0.0061	0.0065	0.0049	0.1627	0.1685
	0.0027	0.0011	0.0028	0.0013	0.0024	0.0009	0.0651	0.0661
glcn_319	0.5747	0.5709	0.2463	0.2624	0.2533	0.2521	0.0542	0.0332
	0.2686	0.2749	0.4948	0.4870	0.5008	0.5047	0.0922	0.0898
	0.1164	0.1147	0.1695	0.1662	0.1605	0.1603	0.1669	0.2068
	0.0326	0.0323	0.0728	0.0694	0.0709	0.0691	0.1987	0.1782
	0.0078	0.0072	0.0166	0.0149	0.0145	0.0139	0.4880	0.4920
glc_319	0.5964	0.5934	0.2048	0.2074	0.1962	0.1993	0.0636	0.0269
	0.2464	0.2590	0.5249	0.5287	0.5389	0.5425	0.0802	0.0728
	0.1134	0.1113	0.1742	0.1725	0.1689	0.1679	0.1339	0.1676
	0.0322	0.0297	0.0776	0.0755	0.0776	0.0751	0.1495	0.1452
	0.0117	0.0067	0.0186	0.0159	0.0185	0.0151	0.5728	0.5875
rib_307	0.5335	0.5350	0.5642	0.5638	0.3697	0.3715	0.0731	0.0622
	0.3083	0.3079	0.2736	0.2743	0.4312	0.4301	0.1500	0.1499
	0.1209	0.1201	0.1261	0.1262	0.1431	0.1426	0.3433	0.3437
	0.0373	0.0370	0.0360	0.0356	0.0560	0.0559	0.4336	0.4442

Table S2: Flux distributions in *Synechocystis* 6863 and related deletion mutants. The data represent the best-fit-solution for each strain and include the estimated fluxes (Mean), the standard deviation (SD) and the corresponding 95% confidence intervals (lower boundary, LB; upper boundary, UB). The ^{13}C labelling data, considered for flux estimation, were taken from GC-MS and from GC-MS plus NMR analysis. The reaction numbers refer to the biochemical network model (Table S9).

	**Biochemical**	**WT (GC-MS)**				**WT (GC-MS+NMR)**				**Δ*eda* (GC-MS)**				**Δ*pfk*AB (GC-MS)**				**Δ*xfp*1 Δ*xfp2* (GC-MS)**			
**No**	**reaction**	**Mean**	SD	LB	UB	**Mean**	SD	LB	UB	**Mean**	SD	LB	UB	**Mean**	SD	LB	UB	**Mean**	SD	LB	UB
R1	GLC_EX = G6P	**100.0**	0.0	100.0	100.0	**100.0**	0.0	100.0	100.0	**100.0**	0.0	100.0	100.0	**100.0**	0.0	100.0	100.0	**100.0**	0.0	100.0	100.0
R2	CO2_EX = CO2	**236.8**	7.3	222.5	251.2	**243.4**	4.9	233.8	252.9	**263.3**	6.3	251.0	275.6	**259.2**	8.7	242.1	276.3	**255.9**	6.8	242.6	269.1
R3	G6P = F6P	**63.1**	2.1	59.0	67.3	**66.3**	1.8	62.7	69.8	**63.4**	1.9	59.7	67.2	**67.6**	2.2	63.2	72.1	**68.0**	2.1	63.9	72.1
R4	F6P = F16BP	**-87.2**	8.4	-103.8	-70.7	**-82.6**	10.1	-102.4	-62.8	**-101.9**	10.0	-121.5	-82.3	-103.8	11.3	-65.2	-109.4	**-91.1**	4.6	-100.1	-82.2
R5	F16BP = DHAP + GAP	**-87.2**	8.4	-103.8	-70.7	**-82.6**	10.1	-102.4	-62.8	**-101.9**	10.0	-121.5	-82.3	-103.8	11.3	-65.2	-109.4	**-91.1**	4.6	-100.1	-82.2
R6	DHAP = GAP	**-176.9**	2.8	-182.4	-171.4	**-183.2**	2.1	-187.3	-179.1	**-192.9**	3.1	-199.0	-186.8	-182.4	2.5	-185.2	-194.9	**-194.0**	2.8	-199.5	-188.5
R7	GAP = 3PG	**-476.2**	6.7	-489.3	-463.1	**-493.6**	5.3	-503.9	-483.3	**-514.9**	8.1	-530.7	-499.1	-489.3	5.9	-500.0	-523.1	**-520.2**	7.1	-534.2	-506.2
R8	3PG = PEP	**200.5**	3.0	194.6	206.5	**207.5**	3.9	199.9	215.1	**198.0**	5.0	-188.1	-207.8	**209.9**	3.4	-203.3	216.5	**216.5**	2.2	212.3	220.8
R9	PEP = PYR	**129.7**	3.7	122.4	136.9	**135.7**	3.2	129.4	142.0	**129.4**	4.2	121.2	137.6	**135.0**	4.8	125.5	144.5	**143.9**	2.0	139.9	147.9
R10	G6P = 6PG	**9.3**	2.1	5.2	13.4	**6.4**	1.6	3.3	9.5	**1.3**	1.6	-1.8	4.5	**5.1**	2.2	0.8	9.4	**5.4**	2.1	1.3	9.4
R11	6PG = Ru5P + CO2	**9.3**	2.1	5.2	13.3	**6.3**	1.6	3.2	9.4	**1.3**	1.6	-1.8	4.5	**4.9**	2.2	0.6	9.3	**4.7**	2.2	0.4	8.9
R12	Ru5P = X5P	**-239.8**	2.9	-245.5	-234.0	**-249.2**	2.8	-254.8	-243.7	**-254.7**	4.4	-263.3	-246.2	**-257.7**	2.6	-262.8	-252.6	**-262.0**	3.7	-269.2	-254.8
R13	Ru5P = Ri5P	**-98.5**	1.8	-102.1	-95.0	**-103.6**	2.0	-107.5	-99.7	**-109.6**	2.6	-114.8	-104.5	**-107.7**	1.6	-110.9	-104.4	**-110.8**	2.0	-114.8	-106.9
R14	Ri5P + X5P = S7P + G3P	**-115.7**	1.5	-118.7	-112.8	**-120.5**	1.5	-123.3	-117.6	**-124.4**	2.2	-128.7	-120.1	**-124.6**	1.3	-127.2	-122.0	**-127.5**	2.0	-131.3	-123.6
R15	S7P + G3P = E4P + F6P	**-26.0**	8.6	-42.8	-9.3	**-19.8**	10.0	-39.5	-0.2	**-33.3**	10.0	-53.0	-13.7	**-21.8**	11.0	-43.4	-0.3	**-24.7**	4.4	-33.2	-16.1
R16	E4P + X5P = F6P + G3P	**-124.2**	1.5	-127.1	-121.3	**-128.9**	1.4	-131.7	-126.1	**-131.3**	2.2	-135.6	-127.1	**-133.1**	1.3	-135.7	-130.5	**-134.5**	1.8	-138.0	-131.0
R17	6PG = KDPG	**0.0**	0.1	-0.2	0.3	**0.0**	0.1	-0.2	0.3	**0.0**	0.0	0.0	0.0	**0.1**	0.3	-0.4	0.6	**0.7**	0.6	-0.5	1.8
R18	KDPG = PYR + G3P	**0.0**	0.1	-0.2	0.3	**0.0**	0.1	-0.2	0.3	**0.0**	0.0	0.0	0.0	**0.1**	0.3	-0.4	0.6	**0.7**	0.6	-0.5	1.8
R19	Ru5P = RuBP	**347.6**	4.4	339.0	356.1	**359.2**	4.1	351.1	367.3	**365.7**	5.7	354.5	376.9	**370.3**	3.6	363.2	377.3	**377.5**	4.6	368.5	386.4
R20	RuBP + CO2 = 3PG + 3PG	**347.6**	4.4	339.0	356.1	**359.2**	4.1	351.1	367.3	**365.7**	5.7	354.5	376.9	**370.3**	3.6	363.2	377.3	**377.5**	4.6	368.5	386.4
R21	DHAP + E4P = SBP	**89.7**	8.5	73.1	106.3	**100.6**	10.3	80.5	120.8	**91.0**	10.1	71.2	110.9	**102.8**	11.0	81.1	124.4	**102.8**	4.5	93.9	111.7
R22	SBP = S7P	**89.7**	8.5	73.1	106.3	**100.6**	10.3	80.5	120.8	**91.0**	10.1	71.2	110.9	**102.8**	11.0	81.1	124.4	**102.8**	4.5	93.9	111.7
R23	PYR = ACCOA + CO2	**83.1**	4.4	74.4	91.7	**89.3**	2.7	84.1	94.6	**81.2**	4.1	73.3	89.1	**88.1**	5.0	78.2	97.9	**93.3**	1.3	90.8	95.8
R24	ACCOA + OAA = ICIT	**31.9**	2.0	27.9	35.9	**34.4**	1.4	31.7	37.0	**31.9**	2.1	27.8	36.0	**35.7**	1.7	32.3	39.0	**37.9**	0.8	36.3	39.6
R25	ICIT = AKG + CO2	**31.9**	2.0	27.9	35.9	**34.4**	1.4	31.7	37.0	**31.9**	2.1	27.8	36.0	**35.7**	1.7	32.3	39.0	**37.9**	0.8	36.3	39.6
R26	AKG = SucA + CO2	**0.4**	0.4	-0.4	1.1	**0.6**	0.3	0.0	1.3	**1.7**	0.6	0.6	2.8	**1.1**	0.5	0.1	2.2	**3.4**	0.6	2.2	4.5

R27	SucA = SUC	**0.4**	0.4	-0.4	1.1	**0.6**	0.3	0.0	1.3	**1.7**	0.6	0.6	2.8	**1.1**	0.5	0.1	2.2	**3.4**	0.6	2.2	4.5
R28	SUC = 0.5 FUM + 0.5 FUM	**0.4**	0.4	-0.4	1.1	**0.6**	0.3	0.0	1.3	**1.7**	0.6	0.6	2.8	**1.1**	0.5	0.1	2.2	**3.4**	0.6	2.2	4.5
R29	FUM = MAL	**0.4**	0.4	-0.4	1.1	**0.6**	0.3	0.0	1.3	**1.7**	0.6	0.6	2.8	**1.1**	0.5	0.1	2.2	**3.4**	0.6	2.2	4.5
R30	MAL = OAA	**0.4**	0.4	-0.4	1.1	**0.6**	0.3	0.0	1.3	**1.7**	0.6	0.6	2.8	**1.1**	0.5	0.1	2.2	**3.4**	0.6	2.2	4.5
R31	MAL = PYR + CO2	**0.0**	0.0	0.0	0.0	**0.0**	0.0	0.0	0.0	**0.0**	0.0	0.0	0.0	**0.0**	0.0	0.0	0.0	**0.0**	0.0	0.0	0.0
R32	PEP + CO2 = OAA	**53.7**	2.2	49.4	58.0	**54.7**	2.0	50.9	58.5	**53.4**	2.5	48.5	58.2	**57.9**	2.4	53.3	62.6	**58.6**	1.6	55.5	61.6
R33	X5P = ACCOA + G3P	**0.1**	0.4	-0.6	0.9	**0.1**	0.2	-0.4	0.5	**1.0**	1.4	-1.8	3.8	**0.0**	0.2	-0.3	0.3	**0.0**	0.0	0.0	0.0
R34	F6P = ACCOA + E4P	**0.1**	0.4	-0.7	0.9	**0.1**	0.4	-0.7	1.0	**0.6**	1.0	-1.4	2.6	**0.0**	0.0	-0.1	0.1	**0.0**	0.0	0.0	0.0
R35	PYR + PYR = VAL + CO2	**5.5**	0.6	4.4	6.7	**5.3**	0.4	4.5	6.1	**6.0**	0.4	5.3	6.7	**5.8**	0.7	4.4	7.2	**6.5**	0.3	5.9	7.0
R36	PYR + OAA = ILE + CO2	**5.2**	0.5	4.1	6.2	**4.9**	0.4	4.2	5.7	**5.6**	0.3	4.9	6.2	**5.5**	0.6	4.2	6.7	**6.0**	0.2	5.6	6.5
R37	E4P + PEP = SHKM	**8.6**	0.1	8.5	8.7	**8.5**	0.1	8.3	8.8	**7.6**	0.5	6.5	8.6	**8.5**	0.2	8.2	8.8	**7.0**	0.7	5.7	8.3
R38	SHKM + PEP = CHRM	**8.6**	0.1	8.5	8.7	**8.5**	0.1	8.3	8.8	**7.6**	0.5	6.5	8.6	**8.5**	0.2	8.2	8.8	**7.0**	0.7	5.7	8.3
R39	CHRM = PHE + CO2	**4.1**	0.0	4.0	4.1	**4.1**	0.0	4.0	4.1	**3.6**	0.3	3.0	4.2	**4.0**	0.1	3.9	4.2	**3.2**	0.4	2.4	4.0
R40	CHRM = TYR + CO2	**3.0**	0.0	2.9	3.0	**3.0**	0.0	2.9	3.0	**2.6**	0.2	2.2	3.1	**2.9**	0.0	2.8	3.0	**2.3**	0.3	1.8	2.9
R41	PYR + PYR = ISV + CO2	**10.8**	0.9	9.0	12.5	**11.3**	0.5	10.4	12.2	**10.5**	0.4	9.8	11.2	**10.3**	1.2	7.9	12.7	**11.2**	0.1	11.0	11.4
R42	ISV + ACCOA = LEU + CO2	**10.8**	0.9	9.0	12.5	**11.3**	0.5	10.4	12.2	**10.5**	0.4	9.8	11.2	**10.3**	1.2	7.9	12.7	**11.2**	0.1	11.0	11.4
R43	CHRM = ANTHR + PYR	**1.6**	0.0	1.5	1.6	**1.5**	0.1	1.3	1.7	**1.3**	0.2	1.0	1.6	**1.5**	0.1	1.4	1.7	**1.5**	0.0	1.5	1.5
R44	ANTHR + Ri5P = CPADP5P	**1.6**	0.0	1.5	1.6	**1.5**	0.1	1.3	1.7	**1.3**	0.2	1.0	1.6	**1.5**	0.1	1.4	1.7	**1.5**	0.0	1.5	1.5
R45	CPADP5P = INDG + CO2	**1.6**	0.0	1.5	1.6	**1.5**	0.1	1.3	1.7	**1.3**	0.2	1.0	1.6	**1.5**	0.1	1.4	1.7	**1.5**	0.0	1.5	1.5
R46	INDG = IND + G3P	**1.6**	0.0	1.5	1.6	**1.5**	0.1	1.3	1.7	**1.3**	0.2	1.0	1.6	**1.5**	0.1	1.4	1.7	**1.5**	0.0	1.5	1.5
R47	IND + 3PG = TRP	**1.6**	0.0	1.5	1.6	**1.5**	0.1	1.3	1.7	**1.3**	0.2	1.0	1.6	**1.5**	0.1	1.4	1.7	**1.5**	0.0	1.5	1.5
R48	OAA + PYR = LYS + CO2	**3.5**	0.4	2.8	4.2	**3.3**	0.3	2.8	3.8	**3.7**	0.2	3.2	4.1	**3.7**	0.4	2.8	4.5	**4.0**	0.2	3.7	4.3
R49	GLU = PRO	**4.6**	0.3	4.0	5.3	**4.8**	0.3	4.2	5.4	**4.3**	0.4	3.6	5.0	**5.0**	0.3	4.4	5.5	**5.0**	0.1	4.7	5.2
R50	GLU + CO2 = ARG	**4.5**	0.3	3.9	5.2	**4.8**	0.3	4.2	5.3	**4.2**	0.3	3.5	4.9	**4.9**	0.3	4.3	5.4	**4.9**	0.1	4.7	5.1
R51	PYR = ALA	**6.9**	0.7	5.5	8.3	**6.5**	0.3	5.9	7.1	**7.3**	0.5	6.3	8.4	**7.2**	1.0	5.4	9.1	**7.5**	0.8	5.9	9.1
R52	OAA = ASP	**9.0**	0.9	7.3	10.8	**8.5**	0.5	7.6	9.4	**9.3**	0.7	7.9	10.7	**9.5**	1.2	7.2	11.8	**9.4**	0.9	7.7	11.1
R53	OAA = THR	**4.5**	0.5	3.6	5.4	**4.2**	0.2	3.8	4.7	**4.6**	0.4	3.8	5.3	**4.7**	0.6	3.6	5.9	**4.6**	0.5	3.7	5.5
R54	SER = GLY + C1	**6.2**	0.7	4.9	7.4	**5.7**	0.3	5.1	6.4	**6.4**	0.5	5.5	7.3	**6.4**	0.8	4.8	8.0	**6.1**	0.6	5.0	7.3
R55	SER = CYS	**0.8**	0.1	0.7	1.0	**0.8**	0.1	0.7	0.9	**0.8**	0.1	0.7	0.9	**0.9**	0.1	0.7	1.1	**0.8**	0.1	0.7	1.0
R56	3PG = SER	**11.7**	1.2	9.4	14.0	**11.0**	0.6	9.9	12.1	**12.1**	0.9	10.3	13.8	**12.3**	1.5	9.3	15.3	**11.6**	1.1	9.6	13.7
R57	AKG = GLU	**19.4**	1.3	16.9	21.9	**20.6**	0.9	18.9	22.4	**18.4**	1.3	15.8	21.0	**21.2**	1.0	19.2	23.1	**21.2**	0.4	20.4	22.0
R58	ASP + C1 = MET	**1.6**	0.1	1.3	1.9	**1.5**	0.1	1.4	1.7	**1.6**	0.1	1.4	1.9	**1.7**	0.2	1.3	2.1	**1.6**	0.1	1.4	1.9
R59	Ri5P + C1 = HIS	**1.9**	0.1	1.7	2.0	**1.8**	0.1	1.6	2.0	**1.6**	0.2	1.3	1.9	**1.8**	0.1	1.6	2.0	**1.8**	0.1	1.6	1.9
R60	C1 = C1X	**0.8**	0.5	-0.2	1.8	**0.5**	0.3	-0.1	1.1	**1.4**	0.5	0.4	2.3	**1.0**	0.6	-0.1	2.2	**0.9**	0.5	-0.2	1.9

R61	G6P = G6PB	**27.6**	0.5	26.6	28.5	**27.4**	0.8	25.8	28.9	**35.2**	1.1	33.1	37.4	**27.3**	0.4	26.6	28.0	**26.6**	0.3	26.1	27.2
R62	Ri5P = Ri5PB	**13.8**	0.5	12.8	14.8	**13.5**	0.9	11.7	15.4	**11.8**	1.3	9.2	14.5	**13.6**	0.7	12.3	14.9	**13.3**	0.5	12.5	14.2
R63	ACCOA = ACCOAB	**40.7**	3.7	33.4	47.9	**43.9**	2.5	39.0	48.7	**40.4**	2.6	35.2	45.5	**42.1**	3.7	34.9	49.3	**44.2**	0.7	42.7	45.6
R64	AKG = AKGB	**12.1**	0.9	10.3	13.9	**13.1**	0.6	11.8	14.4	**11.8**	0.8	10.3	13.4	**13.4**	0.6	12.1	14.6	**13.4**	0.2	12.9	13.8
R65	3PG = 3PGB	**5.1**	0.5	4.1	6.0	**4.8**	0.2	4.4	5.2	**5.2**	0.5	4.3	6.1	**5.3**	0.7	4.0	6.7	**5.0**	0.5	4.1	6.0
R66	ALA = ALAB	**6.9**	0.7	5.5	8.3	**6.5**	0.3	5.9	7.1	**7.3**	0.5	6.3	8.4	**7.2**	1.0	5.4	9.1	**7.5**	0.8	5.9	9.1
R67	ARG = ARGB	**4.5**	0.3	3.9	5.2	**4.8**	0.3	4.2	5.3	**4.2**	0.3	3.5	4.9	**4.9**	0.3	4.3	5.4	**4.9**	0.1	4.7	5.1
R68	ASP = ASPB	**7.4**	0.8	5.9	8.9	**7.0**	0.4	6.2	7.8	**7.7**	0.6	6.4	8.9	**7.8**	1.0	5.9	9.8	**7.7**	0.8	6.2	9.3
R69	CYS = CYSB	**0.8**	0.1	0.7	1.0	**0.8**	0.1	0.7	0.9	**0.8**	0.1	0.7	0.9	**0.9**	0.1	0.7	1.1	**0.8**	0.1	0.7	1.0
R70	GLU = GLUB	**10.3**	0.8	8.7	11.9	**11.1**	0.5	10.0	12.1	**9.9**	0.7	8.5	11.4	**11.3**	0.5	10.3	12.3	**11.3**	0.2	10.9	11.8
R71	GLY = GLYB	**6.2**	0.7	4.9	7.4	**5.7**	0.3	5.1	6.4	**6.4**	0.5	5.5	7.3	**6.4**	0.8	4.8	8.0	**6.1**	0.6	5.0	7.3
R72	ILE = ILEB	**5.2**	0.5	4.1	6.2	**4.9**	0.4	4.2	5.7	**5.6**	0.3	4.9	6.2	**5.5**	0.6	4.2	6.7	**6.0**	0.2	5.6	6.5
R73	LEU = LEUB	**10.8**	0.9	9.0	12.5	**11.3**	0.5	10.4	12.2	**10.5**	0.4	9.8	11.2	**10.3**	1.2	7.9	12.7	**11.2**	0.1	11.0	11.4
R74	LYS = LYSB	**3.5**	0.4	2.8	4.2	**3.3**	0.3	2.8	3.8	**3.7**	0.2	3.2	4.1	**3.7**	0.4	2.8	4.5	**4.0**	0.2	3.7	4.3
R75	PHE = PHEB	**4.1**	0.0	4.0	4.1	**4.1**	0.0	4.0	4.1	**3.6**	0.3	3.0	4.2	**4.0**	0.1	3.9	4.2	**3.2**	0.4	2.4	4.0
R76	PRO = PROB	**4.6**	0.3	4.0	5.3	**4.8**	0.3	4.2	5.4	**4.3**	0.4	3.6	5.0	**5.0**	0.3	4.4	5.5	**5.0**	0.1	4.7	5.2
R77	SER = SERB	**4.7**	0.5	3.8	5.7	**4.5**	0.2	4.1	4.9	**4.9**	0.4	4.1	5.7	**5.0**	0.6	3.7	6.2	**4.7**	0.5	3.8	5.6
R78	THR = THRB	**4.5**	0.5	3.6	5.4	**4.2**	0.2	3.8	4.7	**4.6**	0.4	3.8	5.3	**4.7**	0.6	3.6	5.9	**4.6**	0.5	3.7	5.5
R79	TRP = TRPB	**1.6**	0.0	1.5	1.6	**1.5**	0.1	1.3	1.7	**1.3**	0.2	1.0	1.6	**1.5**	0.1	1.4	1.7	**1.5**	0.0	1.5	1.5
R80	TYR = TYRB	**3.0**	0.0	2.9	3.0	**3.0**	0.0	2.9	3.0	**2.6**	0.2	2.2	3.1	**2.9**	0.0	2.8	3.0	**2.3**	0.3	1.8	2.9
R81	VAL = VALB	**5.5**	0.6	4.4	6.7	**5.3**	0.4	4.5	6.1	**6.0**	0.4	5.3	6.7	**5.8**	0.7	4.4	7.2	**6.5**	0.3	5.9	7.0
R82	MET = METB	**1.6**	0.1	1.3	1.9	**1.5**	0.1	1.4	1.7	**1.6**	0.1	1.4	1.9	**1.7**	0.2	1.3	2.1	**1.6**	0.1	1.4	1.9
R83	HIS = HISB	**1.9**	0.1	1.7	2.0	**1.8**	0.1	1.6	2.0	**1.6**	0.2	1.3	1.9	**1.8**	0.1	1.6	2.0	**1.8**	0.1	1.6	1.9
R84	C1 = C1B	**1.9**	0.1	1.8	2.0	**1.9**	0.1	1.7	2.0	**1.8**	0.0	1.7	1.9	**1.9**	0.0	1.8	2.0	**1.8**	0.1	1.7	2.0

Table S3: Measured and simulated GC-MS and NMR ^{13}C labelling data for ^{13}C metabolic flux analysis of photomixotrophic *Synechocystis* 6803. The approach involved four parallel isotope studies on different ^{13}C glucose tracers. The data represent the best-fit solution after minimizing the variance-weighted sum of square residuals and display the experimentally measured (exp) and model simulated (sim) GC-MS mass isotopomer distributions of amino acids, sugars, and sugar derivatives plus positional enrichments from NMR analysis. Regarding GC-MS analysis, the specified fragments represent the ion clusters considered for the analysis, whereby the number denotes the corresponding monoisotopic mass. Data has been corrected for natural occurring isotopes. For NMR, the assessed carbon atom is given. The flux fit was statistically acceptable. The variance-weighted sum of squared residuals (SSR) was 583 and thus within the expected range (511; 621) of the chi-square test at 95% confidence level.

Fragment	[1-^{13}C] glucose		[3-^{13}C] glucose		[6 -^{13}C] glucose		[$^{13}C_6$] glucose	
	exp	sim	exp	sim	exp	sim	exp	sim
ala_260	0.6734	0.6766	0.7325	0.7317	0.7033	0.7026	0.1060	0.0983
	0.3182	0.3148	0.2346	0.2373	0.2910	0.2912	0.2071	0.2166
	0.0084	0.0086	0.0318	0.0306	0.0056	0.0062	0.4896	0.4817
	0.0000	0.0001	0.0010	0.0004	0.0000	0.0000	0.1973	0.2035
ala_232	0.6811	0.6875	0.7445	0.7465	0.7093	0.7112	0.1129	0.1061
	0.3159	0.3092	0.2283	0.2263	0.2881	0.2858	0.2234	0.2256
	0.0029	0.0033	0.0273	0.0272	0.0026	0.0030	0.6637	0.6683
gly_246	0.9674	0.9720	0.7531	0.7657	0.9736	0.9773	0.1537	0.1521
	0.0326	0.0278	0.2424	0.2307	0.0264	0.0226	0.6250	0.6320
	0.0000	0.0002	0.0045	0.0036	0.0000	0.0001	0.2213	0.2159
gly_218	0.9793	0.9881	0.7663	0.7810	0.9842	0.9890	0.1679	0.1643
	0.0207	0.0119	0.2337	0.2190	0.0158	0.0110	0.8321	0.8357
val_288	0.4643	0.4652	0.5514	0.5462	0.5050	0.4997	0.0268	0.0104
	0.4202	0.4256	0.3355	0.3427	0.4001	0.4079	0.0533	0.0451
	0.1102	0.1054	0.0969	0.0964	0.0913	0.0898	0.1527	0.1656
	0.0038	0.0037	0.0133	0.0137	0.0024	0.0027	0.2522	0.2750
	0.0007	0.0000	0.0023	0.0009	0.0005	0.0000	0.3763	0.3678
	0.0008	0.0000	0.0007	0.0000	0.0006	0.0000	0.1387	0.1360
val_260	0.4632	0.4727	0.5532	0.5572	0.5025	0.5058	0.0269	0.0112
	0.4227	0.4252	0.3372	0.3379	0.4023	0.4065	0.0555	0.0479
	0.1100	0.1001	0.0953	0.0919	0.0920	0.0860	0.1709	0.1927
	0.0037	0.0020	0.0125	0.0123	0.0028	0.0017	0.2815	0.3016
	0.0005	0.0000	0.0019	0.0007	0.0005	0.0000	0.4651	0.4466
leu_274	0.3367	0.3290	0.5233	0.5228	0.3782	0.3637	0.0031	0.0031
	0.4290	0.4396	0.3487	0.3514	0.4216	0.4344	0.0254	0.0213
	0.1991	0.1989	0.1074	0.1070	0.1726	0.1760	0.0916	0.0875

	0.0338	0.0318	0.0173	0.0172	0.0264	0.0254	0.2187	0.2225
	0.0009	0.0006	0.0027	0.0015	0.0007	0.0005	0.3357	0.3413
	0.0006	0.0000	0.0006	0.0000	0.0004	0.0000	0.3255	0.3245
ile_274	0.4509	0.4637	0.5406	0.5491	0.4899	0.4989	0.0255	0.0104
	0.4230	0.4261	0.3388	0.3411	0.4034	0.4079	0.0540	0.0450
	0.1168	0.1063	0.1009	0.0954	0.0992	0.0904	0.1639	0.1807
	0.0064	0.0039	0.0153	0.0135	0.0050	0.0029	0.2692	0.2923
	0.0013	0.0000	0.0030	0.0009	0.0012	0.0000	0.4355	0.4324
	0.0015	0.0000	0.0013	0.0000	0.0013	0.0000	0.0518	0.0391
ser_390	0.6781	0.6766	0.7330	0.7317	0.7067	0.7026	0.1048	0.0983
	0.3141	0.3148	0.2374	0.2373	0.2886	0.2912	0.2099	0.2166
	0.0077	0.0086	0.0288	0.0306	0.0047	0.0062	0.4875	0.4817
	0.0000	0.0001	0.0007	0.0004	0.0000	0.0000	0.1978	0.2035
ser_362	0.6855	0.6875	0.7447	0.7465	0.7134	0.7112	0.1088	0.1061
	0.3139	0.3092	0.2319	0.2263	0.2866	0.2858	0.2311	0.2256
	0.0006	0.0033	0.0235	0.0272	0.0000	0.0030	0.6601	0.6683
thr_404	0.6660	0.6626	0.7256	0.7205	0.6955	0.6919	0.0982	0.0906
	0.3211	0.3223	0.2370	0.2447	0.2962	0.2974	0.1965	0.2066
	0.0113	0.0149	0.0343	0.0339	0.0072	0.0105	0.4612	0.4603
	0.0006	0.0002	0.0022	0.0010	0.0002	0.0001	0.2259	0.2236
	0.0010	0.0000	0.0010	0.0000	0.0009	0.0000	0.0182	0.0189
thr_376	0.6723	0.6744	0.7360	0.7356	0.7002	0.7014	0.1056	0.0979
	0.3215	0.3164	0.2317	0.2340	0.2953	0.2916	0.2104	0.2164
	0.0059	0.0091	0.0311	0.0301	0.0042	0.0069	0.6258	0.6273
	0.0003	0.0001	0.0012	0.0004	0.0004	0.0000	0.0583	0.0585
phe_336	0.3797	0.3762	0.1884	0.2044	0.1821	0.1789	0.0020	0.0004
	0.4188	0.4298	0.4442	0.4510	0.4354	0.4566	0.0049	0.0027
	0.1618	0.1670	0.2554	0.2572	0.2970	0.2957	0.0148	0.0126
	0.0244	0.0255	0.0818	0.0742	0.0705	0.0651	0.0395	0.0369
	0.0028	0.0015	0.0162	0.0120	0.0046	0.0036	0.0889	0.0866
	0.0039	0.0000	0.0057	0.0011	0.0028	0.0001	0.1433	0.1454
	0.0018	0.0000	0.0020	0.0001	0.0015	0.0000	0.2075	0.2156
	0.0022	0.0000	0.0018	0.0000	0.0017	0.0000	0.2174	0.2235
	0.0029	0.0000	0.0030	0.0000	0.0030	0.0000	0.2026	0.2072
	0.0017	0.0000	0.0016	0.0000	0.0014	0.0000	0.0791	0.0691
asp_418	0.6743	0.6626	0.7276	0.7205	0.6990	0.6919	0.1086	0.0906
	0.3141	0.3223	0.2374	0.2447	0.2937	0.2974	0.2015	0.2066
	0.0111	0.0149	0.0332	0.0339	0.0072	0.0105	0.4562	0.4603
	0.0000	0.0002	0.0014	0.0010	0.0000	0.0001	0.2166	0.2236
	0.0004	0.0000	0.0003	0.0000	0.0001	0.0000	0.0171	0.0189
asp_390	0.6775	0.6744	0.7364	0.7356	0.7020	0.7014	0.1109	0.0979
	0.3178	0.3164	0.2334	0.2340	0.2951	0.2916	0.2120	0.2164
	0.0046	0.0091	0.0301	0.0301	0.0029	0.0069	0.6197	0.6273
	0.0000	0.0001	0.0001	0.0004	0.0000	0.0000	0.0574	0.0585
glu_432	0.4680	0.4637	0.5525	0.5491	0.5050	0.4989	0.0248	0.0104
	0.4191	0.4261	0.3356	0.3411	0.4007	0.4079	0.0516	0.0450

	0.1091	0.1063	0.0971	0.0954	0.0918	0.0904	0.1623	0.1807
	0.0035	0.0039	0.0132	0.0135	0.0024	0.0029	0.2717	0.2923
	0.0002	0.0000	0.0015	0.0009	0.0000	0.0000	0.4471	0.4324
	0.0001	0.0000	0.0002	0.0000	0.0001	0.0000	0.0426	0.0391
arg_442	0.4684	0.4556	0.5444	0.5420	0.4988	0.4928	0.0294	0.0097
	0.4118	0.4267	0.3313	0.3438	0.3980	0.4090	0.0577	0.0426
	0.1089	0.1119	0.1025	0.0986	0.0947	0.0942	0.1673	0.1714
	0.0058	0.0057	0.0153	0.0145	0.0037	0.0039	0.2594	0.2846
	0.0013	0.0001	0.0033	0.0011	0.0012	0.0001	0.4226	0.4227
	0.0023	0.0000	0.0020	0.0000	0.0022	0.0000	0.0601	0.0663
	0.0016	0.0000	0.0014	0.0000	0.0014	0.0000	0.0034	0.0027
glcn_319	0.8174	0.8086	0.3493	0.3742	0.3594	0.3581	0.0637	0.0371
	0.1710	0.1840	0.6116	0.5909	0.6179	0.6215	0.0920	0.0946
	0.0107	0.0072	0.0352	0.0340	0.0196	0.0202	0.1639	0.2103
	0.0006	0.0001	0.0028	0.0009	0.0031	0.0002	0.1773	0.1500
	0.0003	0.0000	0.0011	0.0000	0.0000	0.0000	0.5032	0.5080
glc_319	0.8466	0.8425	0.2902	0.2914	0.2780	0.2789	0.0727	0.0287
	0.1313	0.1517	0.6689	0.6745	0.6917	0.6982	0.0729	0.0731
	0.0124	0.0057	0.0349	0.0333	0.0231	0.0227	0.1244	0.1625
	0.0042	0.0001	0.0033	0.0008	0.0035	0.0002	0.1271	0.1168
	0.0055	0.0000	0.0028	0.0000	0.0037	0.0000	0.6029	0.6188
rib_307	0.7500	0.7519	0.7923	0.7921	0.5175	0.5220	0.0868	0.0724
	0.2426	0.2416	0.1827	0.1847	0.4719	0.4678	0.1560	0.1596
	0.0073	0.0064	0.0240	0.0229	0.0106	0.0101	0.3562	0.3561
	0.0001	0.0000	0.0010	0.0004	0.0000	0.0001	0.4011	0.4120
Ala_C2	0.9676	0.9881	0.7852	0.7810	0.9719	0.9890	0.1701	0.1643
	0.0324	0.0119	0.2148	0.2190	0.0281	0.0110	0.8299	0.8357
Ala_C3	0.7136	0.6961	0.9437	0.9382	0.7347	0.7191	0.3109	0.2735
	0.2864	0.3039	0.0563	0.0618	0.2653	0.2809	0.6891	0.7265
Glu_C2	0.7106	0.7107	0.9641	0.9304	0.7339	0.7326	0.2907	0.2680
	0.2894	0.2893	0.0359	0.0696	0.2661	0.2674	0.7093	0.7320
Gly_C2	0.9789	0.9881	0.7825	0.7810	0.9853	0.9890	0.1732	0.1643
	0.0211	0.0119	0.2175	0.2190	0.0147	0.0110	0.8268	0.8357
His_C6	0.7250	0.6961	0.9530	0.9382	0.7520	0.7191	0.2890	0.2735
	0.2760	0.3039	0.0470	0.0618	0.2490	0.2809	0.7110	0.7265
Ile_C4	0.7180	0.7107	0.9470	0.9304	0.7455	0.7326	0.3039	0.2680
	0.2820	0.2893	0.0530	0.0696	0.2545	0.2674	0.6961	0.7320
Ile_C5	0.9508	0.9811	0.9654	0.9854	0.9692	0.9864	0.9068	0.9158
	0.0492	0.0189	0.0346	0.0146	0.0308	0.0136	0.0932	0.0842
Leu_C2	0.7316	0.6961	0.9589	0.9382	0.7518	0.7191	0.2945	0.2735
	0.2684	0.3039	0.0411	0.0618	0.2482	0.2809	0.7055	0.7265
Leu_C5+C6	0.7103	0.6961	0.9464	0.9382	0.7370	0.7191	0.2797	0.2735
	0.2897	0.3039	0.0536	0.0618	0.2630	0.2809	0.7203	0.7265
Lys_C5	0.6980	0.6961	0.9468	0.9382	0.7137	0.7191	0.2989	0.2735
	0.3020	0.3039	0.0532	0.0618	0.2863	0.2809	0.7011	0.7265
Lys_C6	1.0000	0.9881	0.7903	0.7810	1.0000	0.9890	0.1672	0.1643

	0.0000	0.0119	0.2097	0.2190	0.0000	0.0110	0.8328	0.8357
Pro_C2	0.7047	0.7107	0.9300	0.9304	0.7296	0.7326	0.2883	0.2680
	0.2953	0.2893	0.0700	0.0696	0.2704	0.2674	0.7117	0.7320
Pro_C3	1.0000	0.9734	0.7608	0.7889	1.0000	0.9755	0.1560	0.1698
	0.0000	0.0266	0.2392	0.2111	0.0000	0.0245	0.8440	0.8302
Pro_C5	1.0000	0.9881	0.8232	0.7810	1.0000	0.9890	0.1548	0.1643
	0.0000	0.0119	0.1768	0.2190	0.0000	0.0110	0.8452	0.8357
Ser_C2	0.9762	0.9881	0.7720	0.7810	1.0000	0.9890	0.1595	0.1643
	0.0238	0.0119	0.2280	0.2190	0.0000	0.0110	0.8405	0.8357
Thr_C2	1.0000	0.9734	0.8177	0.7889	1.0000	0.9755	0.1662	0.1698
	0.0000	0.0266	0.1823	0.2111	0.0000	0.0245	0.8338	0.8302
Thr_C3	0.7434	0.7107	0.9376	0.9304	0.7679	0.7326	0.2876	0.2680
	0.2566	0.2893	0.0624	0.0696	0.2321	0.2674	0.7124	0.7320
Thr_C4	0.9813	0.9811	0.9802	0.9854	0.9854	0.9864	0.9140	0.9158
	0.0187	0.0189	0.0198	0.0146	0.0146	0.0136	0.0860	0.0842
Val_C3	0.9970	0.9881	0.7770	0.7810	0.9760	0.9890	0.1720	0.1643
	0.0030	0.0119	0.2230	0.2190	0.0240	0.0110	0.8280	0.8357
Val_C4	0.7177	0.6961	0.9443	0.9382	0.7428	0.7191	0.2964	0.2735
	0.2823	0.3039	0.0557	0.0618	0.2572	0.2809	0.7036	0.7265
Val_C5	0.7057	0.6961	0.9337	0.9382	0.7558	0.7191	0.2693	0.2735
	0.2943	0.3039	0.0663	0.0618	0.2442	0.2809	0.7307	0.7265

Table S4: Measured and simulated GC-MS ^{13}C labelling data for ^{13}C metabolic flux analysis of photomixotrophic *Synechocystis* 6803 *Δeda*. The approach involved four parallel isotope studies on different ^{13}C glucose tracers. The data represent the best-fit solution after minimizing the variance-weighted sum of square residuals and display the experimentally measured (exp) and model simulated (sim) mass isotopomer distributions of amino acids, sugars, and sugar derivatives. The specified fragments represent the ion clusters considered for the analysis, whereby the number denotes the corresponding monoisotopic mass. The flux fit of this mutant was statistically acceptable. The variance-weighted sum of squared residuals (SSR) was 394 and thus within the expected range (343; 435) of the chi-square test at 95% confidence level.

Fragment	[1-^{13}C] glucose		[3-^{13}C] glucose		[6 -^{13}C] glucose		[$^{13}C_6$] glucose	
	exp	sim	exp	sim	exp	sim	exp	sim
ala_260	0.5264	0.5295	0.5759	0.5724	0.5483	0.5473	0.1120	0.1050
	0.3467	0.3443	0.2861	0.2906	0.3300	0.3302	0.2007	0.2086
	0.0987	0.0983	0.1105	0.1098	0.0953	0.0960	0.4430	0.4367
	0.0281	0.0279	0.0275	0.0272	0.0264	0.0265	0.2443	0.2497
ala_232	0.5474	0.5510	0.5997	0.6006	0.5678	0.5686	0.1289	0.1191
	0.3554	0.3519	0.2910	0.2910	0.3378	0.3366	0.2330	0.2334
	0.0972	0.0971	0.1092	0.1084	0.0944	0.0947	0.6381	0.6476
gly_246	0.7594	0.7641	0.6041	0.6147	0.7614	0.7642	0.1536	0.1535
	0.1702	0.1660	0.3038	0.2952	0.1680	0.1659	0.5607	0.5663
	0.0704	0.0699	0.0921	0.0901	0.0706	0.0699	0.2857	0.2802
gly_218	0.8275	0.8293	0.6744	0.6861	0.8289	0.8294	0.1850	0.1789
	0.1725	0.1707	0.3256	0.3139	0.1711	0.1706	0.8150	0.8211
val_288	0.3652	0.3637	0.4365	0.4282	0.3968	0.3887	0.0338	0.0132
	0.3918	0.3980	0.3337	0.3415	0.3806	0.3890	0.0666	0.0494
	0.1771	0.1743	0.1607	0.1619	0.1626	0.1630	0.1628	0.1668
	0.0508	0.0503	0.0515	0.0527	0.0465	0.0469	0.2394	0.2589
	0.0125	0.0118	0.0143	0.0132	0.0112	0.0107	0.3310	0.3378
	0.0025	0.0018	0.0034	0.0026	0.0024	0.0017	0.1764	0.1740
val_260	0.3661	0.3692	0.4397	0.4393	0.3973	0.3945	0.0333	0.0145
	0.3949	0.3998	0.3367	0.3401	0.3829	0.3903	0.0678	0.0541
	0.1767	0.1716	0.1596	0.1581	0.1627	0.1602	0.1835	0.2019
	0.0502	0.0483	0.0503	0.0502	0.0462	0.0450	0.2752	0.2984
	0.0121	0.0110	0.0136	0.0122	0.0109	0.0100	0.4403	0.4312
leu_274	0.2684	0.2562	0.4144	0.4083	0.3018	0.2848	0.0070	0.0042
	0.3794	0.3900	0.3404	0.3462	0.3801	0.3910	0.0232	0.0256
	0.2379	0.2410	0.1671	0.1702	0.2175	0.2238	0.0830	0.0953
	0.0864	0.0859	0.0578	0.0575	0.0764	0.0769	0.2252	0.2242
	0.0227	0.0224	0.0162	0.0148	0.0198	0.0197	0.3378	0.3286

	0.0051	0.0045	0.0040	0.0030	0.0045	0.0038	0.3237	0.3221
ile_274	0.3568	0.3626	0.4285	0.4313	0.3846	0.3878	0.0269	0.0127
	0.3920	0.3987	0.3362	0.3410	0.3825	0.3898	0.0584	0.0479
	0.1807	0.1751	0.1629	0.1607	0.1676	0.1636	0.1621	0.1775
	0.0534	0.0502	0.0530	0.0518	0.0494	0.0467	0.2452	0.2684
	0.0136	0.0116	0.0151	0.0128	0.0125	0.0105	0.3879	0.3850
	0.0034	0.0018	0.0042	0.0024	0.0034	0.0016	0.1196	0.1085
ser_390	0.4631	0.4599	0.4994	0.4972	0.4804	0.4752	0.0993	0.0954
	0.3511	0.3531	0.3103	0.3110	0.3395	0.3426	0.1988	0.2006
	0.1386	0.1393	0.1442	0.1453	0.1349	0.1364	0.4258	0.4227
	0.0472	0.0477	0.0462	0.0464	0.0451	0.0458	0.2760	0.2813
ser_362	0.4897	0.4881	0.5315	0.5318	0.5073	0.5033	0.1189	0.1138
	0.3695	0.3692	0.3222	0.3202	0.3556	0.3572	0.2460	0.2364
	0.1408	0.1426	0.1463	0.1480	0.1370	0.1395	0.6351	0.6498
thr_404	0.4479	0.4458	0.4904	0.4824	0.4659	0.4613	0.0868	0.0826
	0.3514	0.3515	0.3038	0.3103	0.3402	0.3417	0.1698	0.1764
	0.1403	0.1421	0.1452	0.1468	0.1363	0.1390	0.3732	0.3739
	0.0486	0.0491	0.0473	0.0478	0.0463	0.0470	0.2649	0.2597
	0.0118	0.0115	0.0133	0.0127	0.0113	0.0110	0.1053	0.1074
thr_376	0.4578	0.4584	0.5042	0.5006	0.4748	0.4740	0.0976	0.0905
	0.3563	0.3544	0.3058	0.3097	0.3448	0.3438	0.1898	0.1925
	0.1384	0.1397	0.1443	0.1442	0.1351	0.1366	0.5156	0.5155
	0.0475	0.0475	0.0457	0.0456	0.0454	0.0455	0.1971	0.2014
phe_336	0.3036	0.2933	0.1540	0.1677	0.1468	0.1471	0.0021	0.0007
	0.3809	0.3892	0.3759	0.3793	0.3627	0.3812	0.0070	0.0040
	0.2099	0.2173	0.2681	0.2737	0.3035	0.3033	0.0211	0.0168
	0.0718	0.0756	0.1305	0.1253	0.1292	0.1235	0.0493	0.0437
	0.0200	0.0199	0.0454	0.0411	0.0377	0.0355	0.0844	0.0939
	0.0063	0.0040	0.0155	0.0104	0.0111	0.0080	0.1407	0.1457
	0.0024	0.0006	0.0047	0.0021	0.0033	0.0013	0.2041	0.2055
	0.0021	0.0001	0.0025	0.0003	0.0023	0.0002	0.1970	0.2077
	0.0016	0.0000	0.0018	0.0000	0.0018	0.0000	0.1956	0.1918
	0.0015	0.0000	0.0015	0.0000	0.0016	0.0000	0.0985	0.0902
asp_418	0.4479	0.4448	0.4892	0.4814	0.4634	0.4603	0.0865	0.0825
	0.3503	0.3509	0.3025	0.3097	0.3418	0.3410	0.1670	0.1762
	0.1412	0.1428	0.1474	0.1475	0.1371	0.1397	0.3718	0.3736
	0.0490	0.0497	0.0478	0.0484	0.0466	0.0477	0.2674	0.2597
	0.0116	0.0118	0.0130	0.0130	0.0110	0.0113	0.1073	0.1080
asp_390	0.4559	0.4576	0.5023	0.4997	0.4708	0.4731	0.0935	0.0905
	0.3569	0.3538	0.3052	0.3092	0.3472	0.3433	0.1840	0.1924
	0.1393	0.1404	0.1463	0.1450	0.1363	0.1374	0.5207	0.5154
	0.0479	0.0482	0.0462	0.0462	0.0457	0.0462	0.2018	0.2017
glu_432	0.3134	0.3098	0.3742	0.3707	0.3354	0.3333	0.0261	0.0112
	0.3745	0.3792	0.3299	0.3362	0.3694	0.3736	0.0569	0.0436
	0.2066	0.2052	0.1904	0.1889	0.1962	0.1945	0.1524	0.1624
	0.0772	0.0775	0.0748	0.0749	0.0727	0.0726	0.2389	0.2580

	0.0231	0.0231	0.0244	0.0234	0.0213	0.0212	0.3819	0.3770
	0.0053	0.0053	0.0063	0.0059	0.0049	0.0048	0.1438	0.1478
arg_442	0.3103	0.3040	0.3669	0.3635	0.3271	0.3272	0.0245	0.0103
	0.3730	0.3788	0.3269	0.3379	0.3680	0.3738	0.0524	0.0403
	0.2066	0.2084	0.1926	0.1912	0.1984	0.1977	0.1436	0.1503
	0.0768	0.0790	0.0766	0.0763	0.0741	0.0740	0.2224	0.2417
	0.0241	0.0235	0.0260	0.0239	0.0227	0.0215	0.3578	0.3534
	0.0068	0.0053	0.0082	0.0060	0.0070	0.0049	0.1461	0.1468
	0.0024	0.0010	0.0029	0.0013	0.0028	0.0009	0.0532	0.0572
glcn_319	0.5802	0.5691	0.2518	0.2771	0.2564	0.2675	0.0689	0.0453
	0.2636	0.2772	0.4944	0.4762	0.5041	0.4937	0.1096	0.1037
	0.1149	0.1142	0.1669	0.1643	0.1589	0.1579	0.1755	0.2178
	0.0327	0.0324	0.0711	0.0677	0.0673	0.0672	0.1813	0.1717
	0.0087	0.0071	0.0158	0.0146	0.0133	0.0136	0.4647	0.4615
glc_319	0.5954	0.5814	0.2445	0.2458	0.2400	0.2373	0.0760	0.0408
	0.2568	0.2683	0.5009	0.4999	0.5129	0.5154	0.1040	0.0933
	0.1117	0.1124	0.1670	0.1680	0.1629	0.1623	0.1619	0.1961
	0.0293	0.0310	0.0719	0.0712	0.0699	0.0707	0.1560	0.1550
	0.0067	0.0068	0.0158	0.0152	0.0142	0.0142	0.5021	0.5147
rib_307	0.5363	0.5341	0.5683	0.5642	0.3822	0.3855	0.0904	0.0796
	0.3075	0.3094	0.2715	0.2737	0.4228	0.4197	0.1605	0.1628
	0.1194	0.1196	0.1248	0.1265	0.1407	0.1405	0.3428	0.3438
	0.0367	0.0370	0.0354	0.0356	0.0543	0.0542	0.4064	0.4137

Table S5: Measured and simulated GC-MS ^{13}C labelling data for ^{13}C metabolic flux analysis of photomixotrophic *Synechocystis* 6803 *ΔpfkA*/*ΔpfkB*. The approach involved four parallel isotope studies on different ^{13}C glucose tracers. The data represent the best-fit solution after minimizing the variance-weighted sum of square residuals and display the experimentally measured (exp) and model simulated (sim) mass isotopomer distributions of amino acids, sugars, and sugar derivatives. The specified fragments represent the ion clusters considered for the analysis, whereby the number denotes the corresponding monoisotopic mass. The flux fit of this mutant was statistically acceptable. The variance-weighted sum of squared residuals (SSR) was 401 and thus within the expected range (343; 435) of the chi-square test at 95% confidence level.

Fragment	[1-^{13}C] glucose		[3-^{13}C] glucose		[6 -^{13}C] glucose		[$^{13}C_6$] glucose	
	exp	sim	exp	sim	exp	sim	exp	sim
ala_260	0.5161	0.5161	0.5706	0.5733	0.5664	0.5667	0.0920	0.0845
	0.3526	0.3526	0.2908	0.2894	0.3390	0.3383	0.1894	0.1999
	0.1019	0.1019	0.1107	0.1102	0.0946	0.0950	0.4500	0.4460
	0.0294	0.0294	0.0279	0.0271	0.7587	0.7633	0.2687	0.2696
ala_232	0.5428	0.5428	0.5953	0.5961	0.1703	0.1667	0.1080	0.0999
	0.3592	0.3592	0.2956	0.2940	0.0710	0.0700	0.2251	0.2294
	0.0980	0.0980	0.1091	0.1099	0.8280	0.8292	0.6669	0.6707
gly_246	0.7531	0.7531	0.6009	0.6148	0.1720	0.1708	0.1323	0.1334
	0.1757	0.1757	0.3061	0.2955	0.3918	0.3854	0.5554	0.5636
	0.0712	0.0712	0.0930	0.0897	0.3829	0.3902	0.3123	0.3030
gly_218	0.8265	0.8265	0.6724	0.6806	0.1645	0.1645	0.1640	0.1619
	0.1735	0.1735	0.3276	0.3194	0.0471	0.0474	0.8360	0.8381
val_288	0.3535	0.3535	0.4289	0.4254	0.0113	0.0108	0.0229	0.0088
	0.3957	0.3957	0.3387	0.3417	0.0023	0.0017	0.0510	0.0395
	0.1819	0.1819	0.1621	0.1634	0.3942	0.3917	0.1384	0.1455
	0.0530	0.0530	0.0525	0.0534	0.3850	0.3914	0.2306	0.2562
	0.0132	0.0132	0.0144	0.0134	0.1635	0.1615	0.3575	0.3568
	0.0027	0.0027	0.0034	0.0026	0.0464	0.0454	0.1996	0.1932
val_260	0.3603	0.3603	0.4326	0.4322	0.0109	0.0101	0.0253	0.0102
	0.3964	0.3964	0.3421	0.3422	0.2967	0.2813	0.0574	0.0448
	0.1797	0.1797	0.1607	0.1612	0.3812	0.3910	0.1658	0.1808
	0.0510	0.0510	0.0510	0.0517	0.2196	0.2258	0.2768	0.3034
	0.0125	0.0125	0.0136	0.0127	0.0776	0.0779	0.4747	0.4608
leu_274	0.2597	0.2597	0.4070	0.4015	0.0202	0.0200	0.0075	0.0027
	0.3798	0.3798	0.3449	0.3476	0.0046	0.0039	0.0218	0.0191
	0.2419	0.2419	0.1687	0.1733	0.3835	0.3851	0.0790	0.0797
	0.0894	0.0894	0.0589	0.0591	0.3822	0.3908	0.2042	0.2085
	0.0237	0.0237	0.0164	0.0154	0.1680	0.1647	0.3336	0.3370

	0.0055	0.0055	0.0041	0.0031	0.0500	0.0471	0.3540	0.3530
ile_274	0.3466	0.3466	0.4195	0.4252	0.0127	0.0106	0.0222	0.0086
	0.3935	0.3935	0.3417	0.3426	0.0035	0.0016	0.0506	0.0384
	0.1855	0.1855	0.1643	0.1633	0.4740	0.4729	0.1430	0.1541
	0.0559	0.0559	0.0544	0.0531	0.3437	0.3441	0.2410	0.2670
	0.0146	0.0146	0.0155	0.0132	0.1365	0.1369	0.4049	0.4042
	0.0039	0.0039	0.0046	0.0025	0.0458	0.0461	0.1383	0.1277
ser_390	0.4541	0.4541	0.4963	0.4980	0.5031	0.5017	0.0807	0.0770
	0.3558	0.3558	0.3119	0.3100	0.3585	0.3585	0.1837	0.1911
	0.1416	0.1416	0.1451	0.1456	0.1383	0.1398	0.4328	0.4309
	0.0485	0.0485	0.0467	0.0464	0.4620	0.4598	0.3028	0.3011
ser_362	0.4852	0.4852	0.5292	0.5280	0.3417	0.3428	0.0988	0.0958
	0.3729	0.3729	0.3242	0.3225	0.1379	0.1391	0.2324	0.2313
	0.1419	0.1419	0.1466	0.1495	0.0469	0.0472	0.6689	0.6729
thr_404	0.4387	0.4387	0.4832	0.4843	0.0115	0.0110	0.0709	0.0640
	0.3542	0.3542	0.3086	0.3090	0.4728	0.4724	0.1570	0.1641
	0.1440	0.1440	0.1463	0.1466	0.3456	0.3450	0.3677	0.3722
	0.0503	0.0503	0.0485	0.0475	0.1361	0.1369	0.2846	0.2813
	0.0127	0.0127	0.0135	0.0126	0.0455	0.0457	0.1197	0.1184
thr_376	0.4514	0.4514	0.4981	0.4977	0.1460	0.1419	0.0817	0.0735
	0.3589	0.3589	0.3107	0.3110	0.3614	0.3794	0.1800	0.1839
	0.1409	0.1409	0.1447	0.1452	0.3034	0.3068	0.5203	0.5198
	0.0488	0.0488	0.0466	0.0461	0.1307	0.1259	0.2180	0.2228
phe_336	0.2911	0.2911	0.1502	0.1607	0.0385	0.0363	0.0035	0.0003
	0.3792	0.3792	0.3705	0.3780	0.0110	0.0082	0.0063	0.0023
	0.2162	0.2162	0.2732	0.2769	0.0031	0.0013	0.0223	0.0112
	0.0762	0.0762	0.1323	0.1284	0.0021	0.0002	0.0418	0.0332
	0.0216	0.0216	0.0463	0.0425	0.0022	0.0000	0.0836	0.0784
	0.0067	0.0067	0.0154	0.0109	0.0017	0.0000	0.1323	0.1348
	0.0025	0.0025	0.0048	0.0022	0.4602	0.4588	0.1950	0.2020
	0.0021	0.0021	0.0026	0.0004	0.3424	0.3422	0.2089	0.2208
	0.0025	0.0025	0.0028	0.0000	0.1389	0.1399	0.2019	0.2128
	0.0018	0.0018	0.0019	0.0000	0.0473	0.0478	0.1045	0.1042
asp_418	0.4518	0.4518	0.4830	0.4833	0.0112	0.0113	0.0748	0.0640
	0.3453	0.3453	0.3079	0.3085	0.4713	0.4716	0.1589	0.1639
	0.1422	0.1422	0.1475	0.1473	0.3458	0.3444	0.3674	0.3719
	0.0489	0.0489	0.0485	0.0481	0.1369	0.1377	0.2811	0.2813
	0.0118	0.0118	0.0131	0.0129	0.0460	0.0464	0.1179	0.1190
asp_390	0.4567	0.4567	0.4969	0.4968	0.3329	0.3305	0.0827	0.0734
	0.3551	0.3551	0.3104	0.3105	0.3706	0.3744	0.1782	0.1838
	0.1403	0.1403	0.1460	0.1460	0.1969	0.1958	0.5200	0.5197
	0.0480	0.0480	0.0467	0.0466	0.0730	0.0732	0.2192	0.2231
glu_432	0.3268	0.3268	0.3676	0.3650	0.0216	0.0214	0.0217	0.0076
	0.3666	0.3666	0.3351	0.3371	0.0050	0.0048	0.0503	0.0351
	0.2031	0.2031	0.1910	0.1913	0.3302	0.3239	0.1392	0.1414
	0.0755	0.0755	0.0755	0.0765	0.3683	0.3745	0.2360	0.2551

	0.0227	0.0227	0.0244	0.0241	0.1975	0.1992	0.3934	0.3937
	0.0053	0.0053	0.0063	0.0061	0.0735	0.0747	0.1594	0.1671
arg_442	0.3052	0.3052	0.3633	0.3580	0.0221	0.0218	0.0190	0.0066
	0.3717	0.3717	0.3329	0.3386	0.0062	0.0049	0.0457	0.0311
	0.2092	0.2092	0.1918	0.1936	0.0022	0.0009	0.1291	0.1258
	0.0795	0.0795	0.0762	0.0778	0.2532	0.2603	0.2141	0.2327
	0.0246	0.0246	0.0254	0.0245	0.5016	0.4987	0.3600	0.3616
	0.0071	0.0071	0.0077	0.0062	0.1606	0.1591	0.1663	0.1738
	0.0026	0.0026	0.0028	0.0013	0.0699	0.0681	0.0659	0.0683
glcn_319	0.5775	0.5775	0.2456	0.2673	0.0147	0.0138	0.0555	0.0334
	0.2672	0.2672	0.4900	0.4820	0.2472	0.2296	0.0952	0.0920
	0.1153	0.1153	0.1729	0.1665	0.5064	0.5208	0.1646	0.2089
	0.0322	0.0322	0.0739	0.0692	0.1626	0.1635	0.1931	0.1822
	0.0078	0.0078	0.0175	0.0150	0.0697	0.0717	0.4915	0.4835
glc_319	0.5950	0.5950	0.2176	0.2357	0.0141	0.0144	0.0744	0.0299
	0.2555	0.2555	0.5147	0.5061	0.3774	0.3787	0.0948	0.0823
	0.1130	0.1130	0.1741	0.1701	0.4263	0.4248	0.1506	0.1870
	0.0296	0.0296	0.0763	0.0726	0.1415	0.1415	0.1613	0.1635
	0.0069	0.0069	0.0173	0.0155	0.0549	0.0550	0.5189	0.5374
rib_307	0.5340	0.5340	0.5678	0.5665	0.5664	0.5667	0.0778	0.0634
	0.3082	0.3082	0.2725	0.2718	0.3390	0.3383	0.1524	0.1537
	0.1207	0.1207	0.1242	0.1264	0.0946	0.0950	0.3415	0.3463
	0.0371	0.0371	0.0355	0.0353	0.7587	0.7633	0.4283	0.4367

Table S6: Measured and simulated GC-MS ^{13}C labelling data for ^{13}C metabolic flux analysis of photomixotrophic *Synechocystis* 6803 Δ*xfp1*/Δ*xfp2*. The approach involved four parallel isotope studies on different ^{13}C glucose tracers. The data represent the best-fit solution after minimizing the variance-weighted sum of square residuals and display the experimentally measured (exp) and model simulated (sim) mass isotopomer distributions of amino acids, sugars, and sugar derivatives. The specified fragments represent the ion clusters considered for the analysis, whereby the number denotes the corresponding monoisotopic mass. The flux fit of this mutant was statistically acceptable. The variance-weighted sum of squared residuals (SSR) was 415 and thus within the expected range (344; 436) of the chi-square test at 95% confidence level.

Fragment	[1-^{13}C] glucose		[3-^{13}C] glucose		[6 -^{13}C] glucose		[$^{13}C_6$] glucose	
	exp	sim	exp	sim	exp	sim	exp	sim
ala_260	0.5293	0.5311	0.5689	0.5620	0.5383	0.5422	0.0983	0.0899
	0.3437	0.3419	0.2972	0.3023	0.3370	0.3341	0.1954	0.2042
	0.0989	0.0990	0.1065	0.1081	0.0973	0.0967	0.4297	0.4290
	0.0281	0.0280	0.0275	0.0276	0.0274	0.0269	0.2766	0.2769
ala_232	0.5498	0.5570	0.5919	0.5968	0.5591	0.5647	0.1136	0.1065
	0.3527	0.3467	0.3030	0.2977	0.3450	0.3400	0.2311	0.2344
	0.0975	0.0963	0.1051	0.1055	0.0959	0.0953	0.6553	0.6591
gly_246	0.7590	0.7601	0.5970	0.6016	0.7593	0.7633	0.1410	0.1370
	0.1704	0.1695	0.3098	0.3063	0.1695	0.1667	0.5431	0.5520
	0.0707	0.0704	0.0932	0.0920	0.0712	0.0700	0.3159	0.3110
gly_218	0.8265	0.8286	0.6659	0.6797	0.8268	0.8292	0.1717	0.1666
	0.1735	0.1714	0.3341	0.3203	0.1732	0.1708	0.8283	0.8334
val_288	0.3689	0.3689	0.4261	0.4180	0.3853	0.3823	0.0251	0.0100
	0.3914	0.3954	0.3486	0.3520	0.3829	0.3913	0.0570	0.0429
	0.1740	0.1722	0.1583	0.1625	0.1685	0.1659	0.1495	0.1510
	0.0504	0.0500	0.0504	0.0523	0.0485	0.0478	0.2356	0.2572
	0.0126	0.0116	0.0133	0.0128	0.0121	0.0109	0.3331	0.3437
	0.0028	0.0018	0.0033	0.0024	0.0028	0.0017	0.1997	0.1951
val_260	0.3712	0.3777	0.4294	0.4340	0.3883	0.3888	0.0279	0.0116
	0.3931	0.3968	0.3517	0.3485	0.3842	0.3925	0.0637	0.0488
	0.1737	0.1677	0.1571	0.1568	0.1679	0.1627	0.1783	0.1891
	0.0499	0.0472	0.0491	0.0491	0.0479	0.0457	0.2813	0.3047
	0.0121	0.0107	0.0126	0.0116	0.0117	0.0102	0.4488	0.4458
leu_274	0.2716	0.2667	0.4090	0.4071	0.2913	0.2782	0.0062	0.0032
	0.3834	0.3907	0.3554	0.3530	0.3806	0.3910	0.0235	0.0217
	0.2348	0.2346	0.1651	0.1680	0.2231	0.2277	0.0775	0.0867
	0.0836	0.0824	0.0535	0.0554	0.0797	0.0789	0.2145	0.2162
	0.0219	0.0214	0.0139	0.0139	0.0206	0.0203	0.3460	0.3358

	0.0047	0.0042	0.0031	0.0027	0.0046	0.0040	0.3322	0.3364
ile_274	0.3640	0.3667	0.4212	0.4234	0.3803	0.3792	0.0214	0.0095
	0.3926	0.3958	0.3527	0.3498	0.3826	0.3921	0.0534	0.0413
	0.1768	0.1737	0.1599	0.1607	0.1708	0.1677	0.1536	0.1571
	0.0514	0.0503	0.0502	0.0513	0.0511	0.0483	0.2447	0.2655
	0.0126	0.0116	0.0130	0.0124	0.0124	0.0110	0.3775	0.3842
	0.0026	0.0018	0.0031	0.0023	0.0027	0.0017	0.1494	0.1424
ser_390	0.4635	0.4623	0.4938	0.4894	0.4704	0.4704	0.0862	0.0821
	0.3506	0.3505	0.3174	0.3192	0.3464	0.3458	0.1910	0.1961
	0.1386	0.1396	0.1426	0.1446	0.1369	0.1374	0.4181	0.4170
	0.0473	0.0476	0.0462	0.0469	0.0463	0.0464	0.3047	0.3049
ser_362	0.4904	0.4925	0.5247	0.5295	0.4979	0.4992	0.1027	0.1021
	0.3686	0.3658	0.3311	0.3247	0.3628	0.3605	0.2416	0.2363
	0.1410	0.1417	0.1442	0.1458	0.1394	0.1403	0.6557	0.6616
thr_404	0.4525	0.4429	0.4845	0.4708	0.4598	0.4529	0.0796	0.0663
	0.3481	0.3504	0.3126	0.3195	0.3426	0.3456	0.1653	0.1645
	0.1389	0.1448	0.1422	0.1478	0.1379	0.1416	0.3506	0.3549
	0.0484	0.0500	0.0474	0.0491	0.0477	0.0484	0.2788	0.2849
	0.0121	0.0120	0.0132	0.0128	0.0120	0.0114	0.1257	0.1294
thr_376	0.4614	0.4585	0.4970	0.4943	0.4693	0.4671	0.0896	0.0769
	0.3532	0.3526	0.3157	0.3162	0.3473	0.3476	0.1876	0.1874
	0.1381	0.1410	0.1414	0.1435	0.1370	0.1387	0.4979	0.4935
	0.0473	0.0479	0.0458	0.0460	0.0465	0.0466	0.2248	0.2422
phe_336	0.3025	0.2988	0.1511	0.1579	0.1393	0.1399	0.0029	0.0004
	0.3805	0.3876	0.3711	0.3798	0.3589	0.3774	0.0058	0.0028
	0.2082	0.2143	0.2759	0.2817	0.3040	0.3086	0.0203	0.0125
	0.0720	0.0747	0.1284	0.1269	0.1321	0.1274	0.0362	0.0356
	0.0199	0.0198	0.0437	0.0411	0.0393	0.0368	0.0870	0.0812
	0.0061	0.0040	0.0141	0.0102	0.0114	0.0084	0.1304	0.1366
	0.0026	0.0006	0.0048	0.0020	0.0038	0.0014	0.1957	0.2012
	0.0023	0.0001	0.0030	0.0003	0.0028	0.0002	0.2101	0.2191
	0.0037	0.0000	0.0053	0.0000	0.0055	0.0000	0.2065	0.2063
	0.0022	0.0000	0.0028	0.0000	0.0029	0.0000	0.1051	0.1044
asp_418	0.4472	0.4420	0.4806	0.4698	0.4517	0.4520	0.0718	0.0662
	0.3500	0.3497	0.3117	0.3189	0.3474	0.3450	0.1523	0.1643
	0.1421	0.1454	0.1463	0.1485	0.1408	0.1423	0.3438	0.3546
	0.0492	0.0506	0.0484	0.0497	0.0486	0.0490	0.2942	0.2849
	0.0116	0.0122	0.0130	0.0131	0.0116	0.0117	0.1378	0.1300
asp_390	0.4579	0.4577	0.4944	0.4934	0.4608	0.4662	0.0788	0.0768
	0.3550	0.3521	0.3145	0.3157	0.3526	0.3470	0.1725	0.1872
	0.1394	0.1417	0.1445	0.1443	0.1392	0.1395	0.5048	0.4934
	0.0477	0.0485	0.0465	0.0466	0.0474	0.0473	0.2439	0.2425
glu_432	0.3129	0.3147	0.3636	0.3634	0.3229	0.3254	0.0193	0.0085
	0.3756	0.3768	0.3411	0.3431	0.3703	0.3749	0.0459	0.0377
	0.2060	0.2032	0.1907	0.1897	0.2029	0.1982	0.1317	0.1446
	0.0772	0.0770	0.0749	0.0750	0.0759	0.0745	0.2283	0.2546

	0.0230	0.0229	0.0236	0.0231	0.0227	0.0219	0.3892	0.3764
	0.0053	0.0053	0.0060	0.0058	0.0053	0.0050	0.1856	0.1782
arg_442	0.3151	0.3071	0.3592	0.3549	0.3230	0.3189	0.0188	0.0074
	0.3720	0.3762	0.3395	0.3446	0.3673	0.3749	0.0449	0.0333
	0.2038	0.2074	0.1910	0.1928	0.2018	0.2016	0.1245	0.1281
	0.0763	0.0792	0.0750	0.0768	0.0753	0.0761	0.2061	0.2316
	0.0237	0.0237	0.0245	0.0238	0.0233	0.0224	0.3308	0.3452
	0.0068	0.0054	0.0078	0.0059	0.0068	0.0051	0.1923	0.1826
	0.0023	0.0011	0.0030	0.0012	0.0025	0.0010	0.0826	0.0717
glcn_319	0.5670	0.5713	0.2451	0.2660	0.2428	0.2595	0.0557	0.0358
	0.2630	0.2746	0.4863	0.4852	0.5017	0.4993	0.0954	0.0951
	0.1243	0.1146	0.1760	0.1651	0.1633	0.1592	0.1634	0.2042
	0.0361	0.0323	0.0746	0.0689	0.0759	0.0682	0.1853	0.1829
	0.0096	0.0072	0.0179	0.0148	0.0163	0.0138	0.5002	0.4820
glc_319	0.6018	0.5854	0.2187	0.2309	0.2133	0.2253	0.0671	0.0316
	0.2544	0.2646	0.5107	0.5117	0.5261	0.5239	0.1004	0.0839
	0.1095	0.1125	0.1760	0.1692	0.1700	0.1641	0.1639	0.1802
	0.0280	0.0306	0.0774	0.0728	0.0756	0.0721	0.1537	0.1619
	0.0063	0.0069	0.0172	0.0154	0.0150	0.0145	0.5150	0.5424
rib_307	0.5422	0.5352	0.5685	0.5571	0.3762	0.3836	0.0902	0.0686
	0.3041	0.3078	0.2765	0.2812	0.4278	0.4211	0.1648	0.1599
	0.1177	0.1201	0.1203	0.1256	0.1411	0.1409	0.3304	0.3396
	0.0360	0.0370	0.0347	0.0361	0.0548	0.0545	0.4146	0.4319

Table S7: Primers used to construct a phosphoketolase double deletion mutant *Δxfp1/Dxfp2* and a single gene deletion mutant *Δgap2* from wild type. In addition, the corresponding annealing temperature (AT) is given.

Primer	Sequence	AT [°C]
XFP1 slr0453 FWD	CTATAGGGCGAATTGGGTACAACTCAACATCAGTGAAGAG	49
XFP1 slr0453 REV	CACCGGATCCCCGGGAATTCAAGTTATTTTGGCGTTAACC	49
XFP2 slr0453 FWD	CACCGGATCCCCGGGAATTCCCTTAACTAAAATCCCTGACAT	50
XFP2 slr0453 REV	AGGGAACAAAAGCTGGAGCTGAGTGGGGAAGAAACCCAAG	54
xfp1	AACTCAACATCAGTGAAGAGGAATTGG	57
xfp2	GAGTGGGGAAGAAACCCAAGAATTAG	57
XFP2_FOR_seq_2	TTTAAAGTCCCCCAAACTTGCCGGAGCTTTAGTGAGGAGATTT	68
XFP2_REV_seq_2	GCAGTTCGGAACAATGGATGGCGGAATATTGGAAAAAGTTACA GAGTT	68
Kan-seq_FOR	AAGCCACGTTGTGTCTCAAAATCTCTGATGTTACATTGC	65
Kan-seq_REV	CTGAGGTCTGCCTCGTGAAGAAGGTGTTGC	66
Gap2out1	GTTAACGTCTACAATCACCTGCCGG	58
Gap2in1	ATCAGAGATTTTGAGACACAACGTGGGTTCGTCTTGCCCTCTC TGTTTTAGC	58
Gap2in2	CTTTCTGGCTGGATGATGGGGCGATGTGGACTTGGCTGAAATT GTGGCTA	58
Gap2out2	CCATAACCCTGAAAGCATTTCTGTG	58

Table S8: Cellular composition used for metabolic flux analysis of *Synechocystis* 6863. The data for wild type (WT) were also used for the strains *ΔpfkAB and Δxfp1/Δxfp2*. For strain Δ*eda*, the data reflect the increased glycogen content.

Biomass constituent	WT [mmol g^{-1}]	Δ*eda* mmol g^{-1}]
G6P	0.0951	0.1236
R5P	0.0480	0.0448
ACCOA	0.1581	0.1478
AKG	0.0480	0.0448
GAP	0.0218	0.0203
ALA	0.0297	0.0278
ARG	0.0176	0.0165
ASP	0.0318	0.0297
CYS	0.0035	0.0033
GLU	0.0406	0.0380
GLY	0.0258	0.0241
ILE	0.0220	0.0205
LEU	0.0399	0.0373
LYS	0.0146	0.0137
PHE	0.0140	0.0131
PRO	0.0179	0.0168
SER	0.0203	0.0190
THR	0.0192	0.0180
TRP	0.0054	0.0051
TYR	0.0102	0.0095
VAL	0.0236	0.0220
MET	0.0069	0.0064
HIS	0.0065	0.0061
C1	0.0077	0.0072

Table S9: Biochemical reaction network for ^{13}C metabolic flux analysis of *Synechocystis* 6803 including reaction stoichiometry, atom transition, and reaction directionality. F = unidirectional (forward only) reaction; FR, reversible reaction; B, biomass. The reactions R1 (v_1) to R34 (v_{34}) refer to the carbon core network of the microbe (Fig. 1). The reactions R35 to R84 represent biomass forming reactions. In Fig. 1 they lumped into the corresponding anabolic fluxes (v_x).

Number	Stoichiometry	Atom transition	Reaction directionality
R1	GLC_EX = G6P	abcdef = abcdef	F
R2	CO2_EX = CO2	a = a	FR
R3	G6P = F6P	abcdef = abcdef	FR
R4	F6P = F16BP	abcdef = abcdef	FR
R5	F16BP = DHAP + GAP	abcdef = abc + def	FR
R6	DHAP = GAP	abc = cba	FR
R7	GAP = 3PG	abc = abc	FR
R8	3PG = PEP	abc = abc	FR
R9	PEP = PYR	abc = abc	F
R10	G6P = 6PG	abcdef = abcdef	F
R11	6PG = Ru5P + CO2	abcdef = bcdef + a	F
R12	Ru5P = X5P	abcde = abcde	FR
R13	Ru5P = Ri5P	abcde = abcde	FR
R14	Ri5P + X5P = S7P + G3P	abcde + fghij = fgabcde + hij	FR
R15	S7P + G3P = E4P + F6P	abcdefg + hij = defg + abchij	FR
R16	E4P + X5P = F6P + G3P	abcd + efghi = efabcd + ghi	FR
R17	6PG = KDPG	abcdef = abcdef	F
R18	KDPG = PYR + G3P	abcdef = abc + def	F
R19	Ru5P = RuBP	abcde = abcde	F
R20	RuBP + CO2 = 3PG + 3PG	abcde + f = fba + cde	F
R21	DHAP + E4P = SBP	abc + defg = abcdefg	F
R22	SBP = S7P	abcdefg = abcdefg	F
R23	PYR = ACCOA + CO2	abc = bc + a	F
R24	ACCOA + OAA = ICIT	ab + cdef = fedbac	F
R25	ICIT = AKG + CO2	abcdef = abcde + f	F
R26	AKG = SucA + CO2	abcde = edcb + a	F
R27	SucA = SUC	abcd = abcd	F
R28	SUC = 0.5 FUM + 0.5 FUM	abcd = 0.5 abcd + 0.5 dcba	FR
R29	FUM = MAL	abcd = abcd	FR
R30	MAL = OAA	abcd = abcd	FR
R31	MAL = PYR + CO2	abcd = abc + d	F
R32	PEP + CO2 = OAA	abc + d = abcd	F
R33	X5P = ACCOA + G3P	abcde = ba + cde	F
R34	F6P = ACCOA + E4P	abcdef = ba + cdef	F

R35	PYR + PYR = VAL + CO2	abc + def = abefc + d	F
R36	PYR + OAA = ILE + CO2	abc + defg = debfgc + a	F
R37	E4P + PEP = SHKM	abcd + efg = efgabcd	F
R38	SHKM + PEP = CHRM	abcdefg + hij = abcdefghij	F
R39	CHRM = PHE + CO2	abcdefghij = hijbcdefg + a	F
R40	CHRM = TYR + CO2	abcdefghij = hijbcdefg + a	F
R41	PYR + PYR = ISV + CO2	abc + def = abefc + d	F
R42	ISV + ACCOA = LEU + CO2	abcde + fg = fgbcde + a	F
R43	CHRM = ANTHR + PYR	abcdefghij = abcdefg + hij	F
R44	ANTHR + Ri5P = CPADP5P	abcdefg + hijkl = abcdefghijkl	F
R45	CPADP5P = INDG + CO2	abcdefghijkl = abcdfghijkl + e	F
R46	INDG = IND + G3P	abcdefghijk = abcdefgh + ijk	F
R47	IND + 3PG = TRP	abcdefgh + ijk = abcdefghkji	F
R48	OAA + PYR = LYS + CO2	abcd + efg = abcdgf + e	F
R49	GLU = PRO	abcde = abcde	F
R50	GLU + CO2 = ARG	abcde + f = abcdef	F
R51	PYR = ALA	abc = abc	F
R52	OAA = ASP	abcd = abcd	F
R53	OAA = THR	abcd = abcd	F
R54	SER = GLY + C1	abc = ab + c	F
R55	SER = CYS	abc = abc	F
R56	3PG = SER	abc = abc	F
R57	AKG = GLU	abcde = abcde	F
R58	ASP + C1 = MET	abcd + e = abcde	F
R59	Ri5P + C1 = HIS	abcde + f = edcbaf	F
R60	C1 = C1X	a = a	F
R61	G6P = G6PB		B
R62	Ri5P = Ri5PB		B
R63	ACCOA = ACCOAB		B
R64	AKG = AKGB		B
R65	3PG = 3PGB		B
R66	ALA = ALAB		B
R67	ARG = ARGB		B
R68	ASP = ASPB		B
R69	CYS = CYSB		B
R70	GLU = GLUB		B
R71	GLY = GLYB		B
R72	ILE = ILEB		B
R73	LEU = LEUB		B
R74	LYS = LYSB		B
R75	PHE = PHEB		B
R76	PRO = PROB		B
R77	SER = SERB		B
R78	THR = THRB		B
R79	TRP = TRPB		B
R80	TYR = TYRB		B

R81	VAL = VALB		B
R82	MET = METB		B
R83	HIS = HISB		B
R84	C1 = C1B		B
